# An “AND” logic gate–based supramolecular therapeutic nanoplatform for combatting drug-resistant non–small cell lung cancer

**DOI:** 10.1126/sciadv.adp9071

**Published:** 2024-09-25

**Authors:** Qili Huang, Chendi Ding, Wenyan Wang, Li Yang, Yinglong Wu, Wenfeng Zeng, Zimu Li, Zhaoqing Shi, Lin Mei, Xiaowei Zeng, Yanli Zhao, Hongzhong Chen

**Affiliations:** ^1^ School of Pharmaceutical Sciences (Shenzhen), Sun Yat-sen University, Shenzhen 518107, P. R. China.; ^2^ Tianjin Key Laboratory of Biomedical Materials, Key Laboratory of Biomaterials and Nanotechnology for Cancer Immunotherapy, Institute of Biomedical Engineering, Chinese Academy of Medical Sciences & Peking Union Medical College, Tianjin 300192, P. R. China.; ^3^ School of Chemistry, Chemical Engineering, and Biotechnology, Nanyang Technological University, 21 Nanyang Link, Singapore 637371, Singapore.

## Abstract

Despite targeted therapies like epidermal growth factor receptor tyrosine kinase inhibitors (EGFR-TKIs), non–small cell lung cancer (NSCLC) remains a clinical challenge due to drug resistance hampering their efficacy. Here, we designed an “AND” logic gate–based supramolecular therapeutic platform (HA-BPY-GEF-NPs) for the treatment of EGFR-TKI resistant NSCLC. This system integrates both internal and external stimuli–responsive mechanisms that need to be activated in a preset sequence, enabling it to precisely control drug release behavior for enhancing therapeutic precision. By programming the system to respond to sequential near-infrared (NIR) irradiation and enzyme (cathepsin B) inputs, the release of gefitinib is effectively confined to the tumor region. Moreover, the NIR irradiation induces reactive oxygen species production, suppressing tumor growth and inhibiting bypass signaling pathways. The designed drug delivery system offers a highly controlled and targeted therapeutic approach, effectively inhibiting tumor growth, suppressing bypass signaling pathways, and overcoming EGFR-TKI resistance, thus offering a potential solution for maximizing therapeutic benefits.

## Teaser

An “AND” logic gate nanoplatform enhances treatment precision and overcomes drug resistance.

## INTRODUCTION

Lung cancer is a leading cause of cancer-related mortality, with non–small cell lung cancer (NSCLC) accounting for approximately 85% of all lung cancer cases (*1*, *2*). As the most common subtype of lung cancer, NSCLC is a major challenge in clinical oncology. In recent decades, targeted therapies such as epidermal growth factor receptor tyrosine kinase inhibitors (EGFR-TKIs) have been extensively studied and achieved tremendous success in clinical practice (*3*–*5*). Although EGFR-TKIs have shown considerable efficacy in improving patient prognosis and prolonging progression-free survival, the development of drug resistance during treatment remains a challenge. There are multiple mechanisms for drug resistance of EGFR-TKIs, such as EGFR T790M mutation, activation of bypass signal, and histologic transformation (*6*, *7*). Among these mechanisms, the activation of bypass signaling plays a crucial role in the acquisition of resistance to EGFR-TKIs and holds clinical implications for the management of NSCLC (*8*).

To overcome EGFR-TKIs resistance caused by bypass signal activation, new strategies have emerged, including the use of molecule inhibitors or monoclonal antibodies to block the activated bypass signaling (*9*, *10*). However, these current approaches are time and labor intensive, and it may also result in off-target toxicity by inhibiting cell proliferation–related signal pathways in normal cells. Reactive oxygen species (ROS), as important signaling molecules, has been found to play a critical role in regulating intracellular signal pathways (*11*, *12*). In a recent study, Gu *et al.* (*13*) found that increased ROS levels in photodynamic therapy (PDT) resulted in switching off the insulin growth factor-1 receptor (IGF1R) bypass signaling by down-regulating the expression of IGF1R and its downstream signal pathways [mitogen-activated protein kinase (MAPK)/extracellular signal–regulated kinase (ERK) and phosphatidylinositol 3-kinase (PI3K)/AKT)]. Therefore, the combination of EGFR-TKIs and PDT has been considered as a promising strategy for the treatment of EGFR-TKIs resistant NSCLC. PDT uses photosensitizers and light sources to generate ROS (*14*–*16*), which not only directly eliminate tumor cells but also enhance the sensitivity of drug-resistant tumors to gefitinib (GEF).

To fully exploit the potential benefits of combination therapy, it is crucial to develop drug delivery materials that can effectively accommodate and deliver multiple therapeutic agents to the tumor site. Conventional drug delivery systems often fail to achieve the desired therapeutic outcome in combination therapies, mainly due to the difficulty in precisely controlling the proportions of different drug components, insufficient stability, and ineffective release profiles. While nanomedicines have shown promise in effectively targeting tumor tissues, enhancing drug bioavailability, ensuring greater stability, minimizing side effects, and potentially extending patient survival, the journey toward fully realizing these benefits remains a formidable challenge (*17*, *18*). Therefore, there is a pressing need for the design and fabrication of advanced drug delivery materials that can address these challenges and enable precise delivery and release of multiple therapeutic components (*19*, *20*). Supramolecular chemistry provides a robust platform for improving the precision of multicomponent drug delivery systems. Especially for macrocyclic host-guest interactions, these systems offer molecular-level control over composition and enable the incorporation and targeting of drugs in a more efficient manner (*21*–*26*).

In addition to accurate drug loading, the precise control over the drug release process is also necessary for achieving effective therapeutic outcomes. Stimuli-responsive drug release systems offer a promising strategy for efficient drug delivery in clinical settings. These systems can be triggered by internal stimuli, including acidic pH (*27*, *28*), up-regulated ROS (*29*, *30*), overexpressed enzymes (*31*, *32*), high concentrations of adenosine triphosphate (ATP) (*33*, *34*), hypoxia (*35*, *36*), and elevated levels of glutathione within cancer cells (*37*), or by external physical stimuli, such as light, heat, ultrasound, and magnetic field (*38*–*40*). However, the complex tumor microenvironment poses notable challenges in achieving accurate regulation of the drug release process. The heterogeneity and dynamics of the tumor microenvironment limit the precision of drug delivery systems that rely solely on a single stimulus response. Recent studies have explored the application of multi-stimuli–responsive delivery systems for targeted drug delivery (*41*, *42*). Especially for multicomponent drug delivery systems, the application of multi-stimuli–responsive mechanisms allows for programmable release, thus enhancing the precision of drug administration. To this end, stimuli-responsive biomaterials containing logic gates have received a lot of interest in the field of biomedicine due to their ability to detect specific various stimuli and respond accordingly (*43*–*46*).

The concept of logic gates in biomaterials involves incorporating molecular switches or sensors that can interpret specific signals, perform Boolean logic operations, and trigger predetermined response (*47*–*51*). These switches can be programmed to respond to multiple inputs and generate an output based on predefined logical rules, similar to electronic logic gates used in computing. In the field of drug delivery, the concept of logic gates has gained much attention in recent years. Such drug delivery systems containing molecular logic gates have evolved from simple single input—“YES/NO” response systems to more sophisticated Boolean logic computations, such as AND, OR, XOR, and XNOR. Research on logic-gated release systems predominantly focused on OR logic, wherein the drug delivery system could respond to any stimulus and initiate drug release, ensuring sufficient drug release (*52*, *53*). However, considering the intricate nature of physiological environments, the use of OR logic gates can lead to premature drug release in certain regions with abundant stimuli. Consequently, drug delivery systems based on “AND” logic gates are favored because of their stricter release conditions (*54*).

It is critical that supramolecular prodrugs are benefited from the dynamic and reversible property of noncovalent interactions. Therefore, they exhibit the various superiorities, including ease of preparation, outstanding chemical stability, sensitive response to biological environments, mix-and-match combination, precise and flexible ratio control, and the potential to accommodate a series of various drugs without reconstruction (*55*). Furthermore, we wonder whether integrating supramolecular prodrugs with AND logic–gated nanodrug delivery systems would achieve both preferable spatiotemporal treatment accuracy and higher tumor selectivity for drug delivery.

**Fig. 1.**
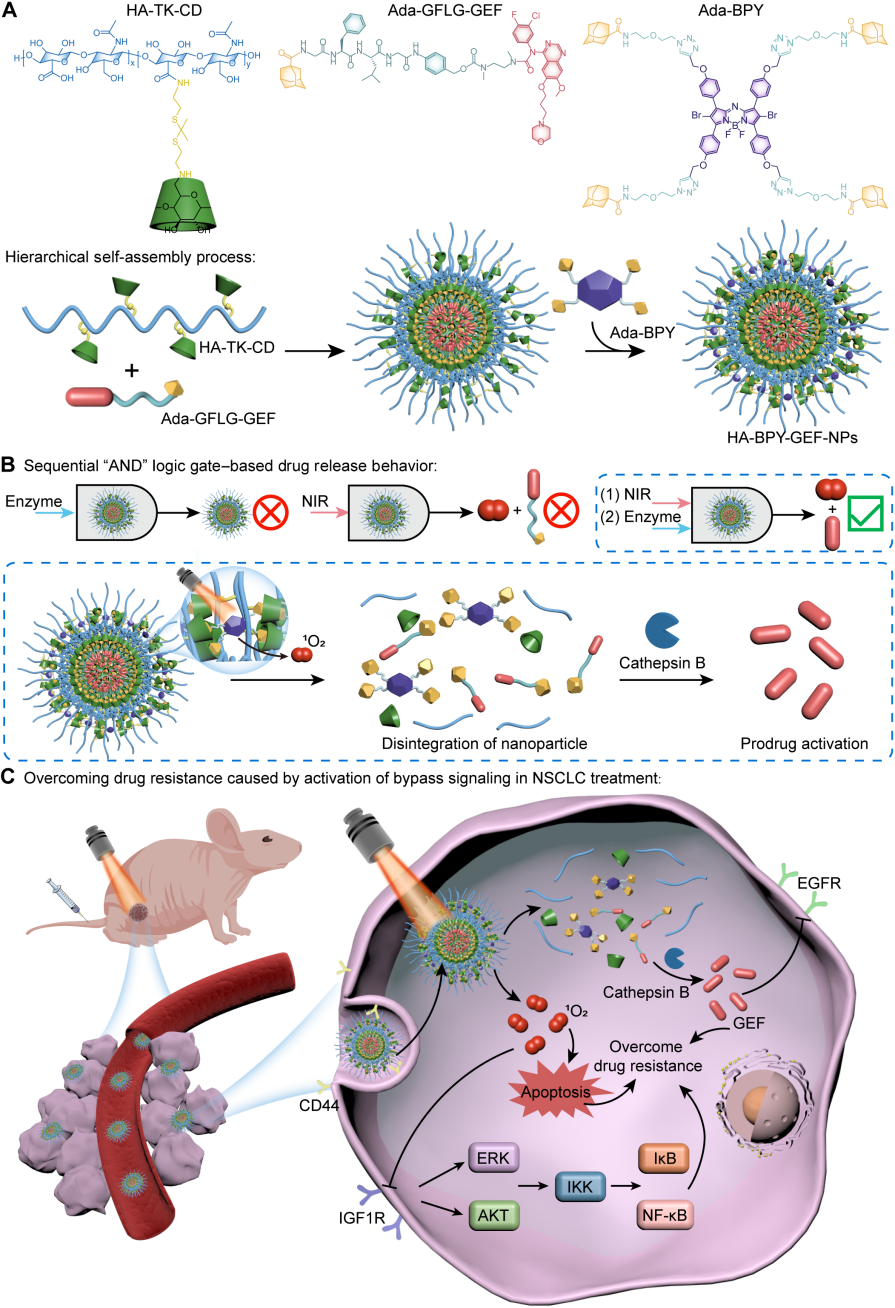
Schematic illustration for the fabrication of supramolecular therapeutic nanoplatform (HA-BPY-GEF-NPs) and its application for drug-resistant tumor therapy. (**A**) Chemical structures of the scaffold polymer carrier (HA-TK-CD), photosensitizer (Ada-BPY), GEF prodrug (Ada-GFLG-GEF), and hierarchical self-assembly process of the HA-BPY-GEF-NPs. (**B**) Sequential AND logic gate–based drug release behavior. (**C**) Mechanism of the HA-BPY-GEF-NPs to overcome drug resistance caused by activation of bypass signaling in NSCLC treatment.

In this study, we designed a kind of AND Boolean logic–based supramolecular drug delivery system (HA-BPY-GEF-NPs) for the treatment of EGFR-TKI–resistant NSCLC (Fig. 1). To fabricate HA-BPY-GEF-NPs, macrocycle molecule β-cyclodextrin (β-CD) was first conjugated to hyaluronic acid (HA) scaffold via ROS-cleavable thioketal bonds. Then, the EGFR-TKI GEF prodrug (Ada-GFLG-GEF), containing adamantane moiety and cathepsin B–degradable peptide sequence GFLG, was anchored to the β-CD sites and encapsulated within the inner layer of the nanoparticle. The adamantane modified photosensitizer Ada-BPY was subsequently loaded to β-CD sites at the outer layer to sequester the inner Ada-GFLG-GEF core region from being contacted with cathepsin B prematurely, thus improving the spatiotemporal accuracy during activation process. The activation mechanism of HA-BPY-GEF-NPs follows an AND logic gate operation, wherein the nanoparticles can only be activated if both near-infrared (NIR) and enzyme inputs are sequentially applied. Initially, the NIR irradiation triggered the generation of ROS through a photodynamic process to cleave the thioketal bonds and liberate the loaded Ada-BPY and Ada-GFLG-GEF. The released Ada-GFLG-GEF was then exposed to the up-regulated cathepsin B in the tumor cells and thus activated to the parental GEF form. The biocomputation capabilities endow HA-BPY-GEF-NPs with highly controlled drug activation properties, enhancing both drug release precision and treatment accuracy. Furthermore, the ROS produced through NIR irradiation not only directly suppresses tumor cell growth but also effectively inhibits bypass signaling pathways. Our study offers an advanced strategy to address EGFR-TKI resistant NSCLC by overcoming drug resistance while minimizing toxicity and providing targeted therapeutic benefits.

## RESULTS

### Fabrication and characterization of supramolecular HA-BPY-GEF-NPs

Herein, we developed supramolecular therapeutic nanoplatforms loaded with Ada-BPY and Ada-GFLG-GEF at different ratios. As depicted in Fig. 1, this system was composed of (i) a hydrophilic HA scaffold, conjugated with β-CDs as side chains through ROS-cleavable thioketal bonds, denoted as HA-TK-CD; (ii) a hydrophobic photosensitizer moiety of Ada-BPY based on boron dipyrromethene as the major skeleton, further modified with four adamantane moieties for supramolecular host-guest complexation; and (iii) a GEF-based prodrug (Ada-GFLG-GEF), which was fabricated through connecting adamantane and GEF through a cathepsin B–degradable GFLG peptide linker. The detailed synthetic procedures for HA-TK-CD, Ada-BPY, and Ada-GFLG-GEF are shown in the Supplementary Materials (figs. S1 to S3). The GFLG linkers could remain intact during blood circulation. Once entered into tumor tissues, they would be cleaved by the overexpressed cathepsin B, which is found in various types of solid tumors (*56*, *57*). This feature has been extensively exploited for developing enzyme-activable prodrugs (*58*, *59*). For comparison, cathepsin B nonsensitive prodrug (Ada-HE-GEF) based on a carbon chain linker was also prepared (fig. S4). All the structures of crucial intermediates and final products were characterized by nuclear magnetic resonance (NMR) and high-resolution mass spectrometry (HR-MS) (fig. S6 to S34).

**Fig. 2.**
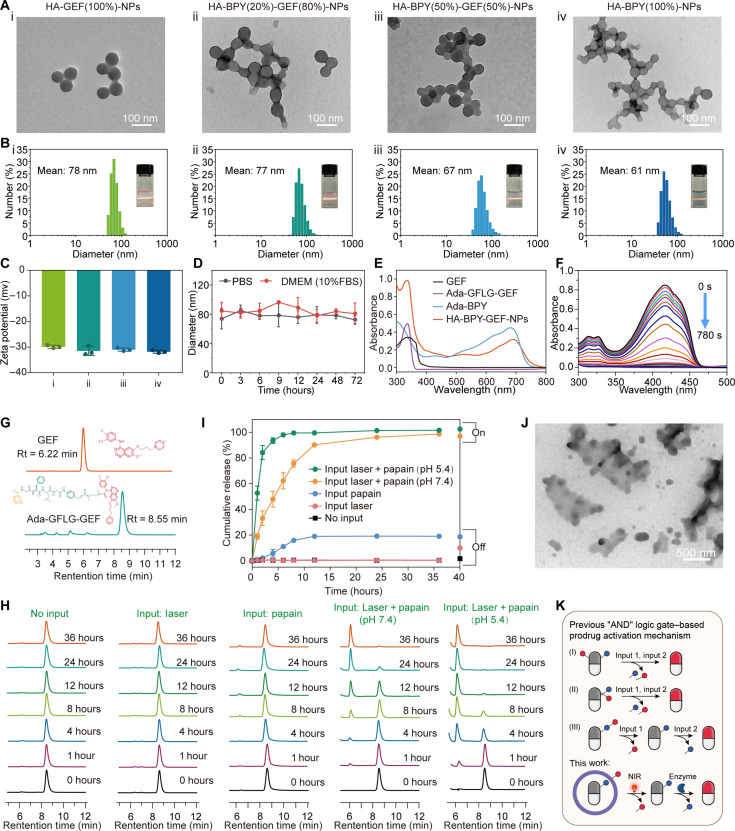
Physicochemical characterization of HA-BPY-GEF-NPs. (**A**) TEM images, (**B**) DLS profiles (Insert, Tyndall light scattering photos of the obtained nanoparticles), and (**C**) Zeta potentials of (i) HA-GEF(100%)-NPs, (ii) HA-BPY(20%)-GEF(80%)-NPs, (iii) HA-BPY(50%)-GEF(50%)-NPs, and (iv) HA-BPY(100%)-NPs. (**D**) Size stability of HA-BPY(20%)-GEF(80%)-NPs during 72 hours of incubation in PBS or DMEM (10% FBS). (**E**) UV-vis absorption spectra of GEF, Ada-GFLG-GEF, Ada-BPY, and HA-BPY-GEF-NPs. (**F**) UV-vis absorption of DPBF gradually quenched in HA-BPY-NPs solutions under laser irradiation (660 nm, 20 mW/cm^2^). (**G**) HPLC profiles of GEF and Ada-GFLG-GEF. (**H**) HPLC profiles of HA-BPY-GEF-NPs after incubation under different conditions. (**I**) GEF release curve. (**J**) TEM image of HA-BPY-GEF-NPs after NIR irradiation (660 nm, 100 mW/cm^2^) for 10 min followed by further incubation with papain (10 μg/ml) for 12 hours. (**K**) Schematic illustration of the models of AND molecular logic gate for designing activatable prodrugs.

The stable binding between β-CD on the carrier scaffold and the adamantane moieties on the guest cargos is crucial for the formation and stability of nanoparticles. Therefore, computational simulation studies were conducted to demonstrate the host-guest interaction during the assembly process and confirm that β-CD could form a stable complexation with the adamantane moieties on Ada-GFLG-GEF and Ada-BPY (fig. S44). Thereafter, a range of formulations with varying loading ratios of the Ada-GFLG-GEF and Ada-BPY were prepared through supramolecular host-guest assembly process. As shown in Fig. 1A, this therapeutic nanoplatform was prepared in two steps. First, Ada-GFLG-GEF was injected into the solution of host polymer HA-TK-CD to obtain the preliminary nanoparticles. At this time, the hydrophobic Ada-GFLG-GEF was encapsulated within as the inner core. Then, Ada-BPY was added to the complex with the outer unoccupied β-CD sites to afford HA-BPY-GEF-NPs in the final form, effectively isolating the internal Ada-GFLG-GEF from the surrounding tumor environment, preventing premature activation and unwanted leakage of GEF. By the above methods, we prepared four kinds of nanoparticles: HA-GEF(100%)-NPs, HA-BPY(20%)-GEF(80%)-NPs, HA-BPY(50%)-GEF(50%)-NPs, and HA-BPY(100%)-NPs. For HA-BPY(20%)-GEF(80%)-NPs, the amount of adamantane moieties contained in the two loaded components were the same. For HA-BPY(50%)-GEF(50%)-NPs, the molar quantities of the photosensitizer component and the prodrug component were the same. Because of the flexibility of supramolecular host-guest assembly, the Ada-GFLG-GEF and Ada-BPY components could be loaded onto the carrier scaffold in varying ratios. For HA-BPY(20%)-GEF(80%)-NPs and HA-BPY(50%)-GEF(50%)-NPs, their corresponding intermediate states were named as HA-GEF(80%)-NPs and HA-GEF(50%)-NPs, respectively. Transmission electron microscopy (TEM) images of these nanoparticles showed uniform spherical morphology with similar particle sizes (Fig. 2A and fig. S45, A and B). Dynamic light scattering (DLS) results also indicated that their similar hydrodynamic diameters were approximately 70 nm (Fig. 2B and fig. S45, C and D) and their zeta potentials were around −30 mV (Fig. 2C and fig. S45E). For all the nanoparticles, no obvious size change was observed over a period of 72 hours in phosphate-buffered saline (PBS) or in complete Dulbecco’s modified Eagle’s medium (DMEM) [containing 10% of fetal bovine serum (FBS)] (Fig. 2D and fig. S45, F to J), indicating that they have good colloidal stability.

Previous studies suggested that IGF1R plays a critical role in the bypass pathways, which are involved in the resistance to EGFR-TKI treatment (*7*). To demonstrate the association between IGF1R expression and resistance to GEF, the expression levels of IGF1R in GEF-sensitive PC-9 cell line and GEF-resistant PC9-GR cell line were assessed through Western blot. The results shown in fig. S46 indicated that the expression level of IGF1R in PC9-GR was obviously higher compared to the GEF-sensitive PC-9 cell line, suggesting that IGF1R protein may contribute to the acquired GEF resistance of NSCLC. Therefore, for subsequent investigations, the PC9-GR cell line characterized by highly expressed IGF1R was used as GEF-resistant cell model and PC-9 cell line was used as GEF-sensitive cell model. First, the in vitro biosafety of the drug carrier scaffold HA-TK-CD in both tumor cells (PC-9 and PC9-GR cells) and nontumor cells [NIH-3T3 and human umbilical vein endothelial cells (HUVECs)] was investigated by cell counting kit-8 (CCK-8) assay. The results demonstrated that after incubation with HA-TK-CD for 24 or 48 hours, both tumor and nontumor cells exhibited high viability even at a concentration as high as 250 μg ml^−1^ (fig. S47), indicating minimal cytotoxicity of HA-TK-CD for further in vivo applications. Next, PC-9 and PC9-GR cells were incubated with different formulations for 48 hours and irradiated with 660-nm laser (100 mW/cm^2^) for 10 min during this period. The cell viability results by the CCK-8 assay were shown in fig. S48, the half-maximal concentration values of free GEF were 0.85 μM for PC-9 cells and 10.45 μM for PC9-GR cells, indicating that GEF exhibited a low cytotoxicity for PC9-GR cells compared to PC-9 cells because PC9-GR is a GEF-resistant cell line. Strikingly, when the cells were incubated with our therapeutic nanoplatform (HA-BPY-GEF-NPs) in combination with 660-nm laser irradiation, the antitumor activity of GEF was enhanced in both PC-9 and PC9-GR cells, suggesting that HA-BPY-GEF-NPs combined with NIR photoirradiation could tackle the problem caused by the resistance of PC-9 cells to GEF. On the basis of the above CCK-8 assay results of PC-9 and PC9-GR cells, it was found that the best synergistic efficacy was achieved when Ada-GFLG-GEF and Ada-BPY were loaded at 4:1 ratio. Therefore, Ada-GFLG-GEF and Ada-BPY with ratios of 10:0, 4:1, and 0:10 were chosen as research subjects and named as HA-GEF-NPs, HA-BPY-GEF-NPs, and HA-BPY-NPs for further study. Moreover, the high-performance liquid chromatography (HPLC) results demonstrated that almost no GEF was released from the cathepsin B nonsensitive Ada-HE-GEF prodrug. The cell viability results showed that HA-GEF-NPs exhibited a more potent anticancer activity than HA-HE-GEF-NPs both in PC-9 and PC9-GR cells, indicating that the cathepsin B specifically cleaving GFLG linker to release active drug is necessary for the antitumor performance (fig. S49).

The spectroscopic and photodynamic properties of HA-BPY-GEF-NPs were then investigated. As shown in Fig. 2E, the ultraviolet-visible (UV-vis) absorption spectrum of HA-BPY-GEF-NPs simultaneously exhibited characteristic peaks of Ada-GFLG-GEF (340 nm) and Ada-BPY (681 nm), indicating the successful loading of these two components. To detect the singlet oxygen (^1^O_2_) generation ability of HA-BPY-NPs with 660-nm laser irradiation, 1,3-diphenylisobenzofuran (DPBF) was used as a probe. DPBF absorption peak at 423 nm was rapidly quenched in HA-BPY-NPs solution under 660-nm laser irradiation (Fig. 2F and fig. S50), indicating that the HA-BPY-NPs have superior ROS-generation capacity. The sequential AND logic gate–based drug release behavior was subsequently studied. Although both TEM and DLS (fig. S51, A and B) results revealed the collapse of nanoparticles and increased particle size of HA-BPY-GEF-NPs after 10 min of NIR irradiation, there was almost no free GEF detected during HPLC analysis (Fig. 2, G to I), which suggested the integrity of Ada-GFLG-GEF component due to the lack of papain.

Since papain and cathepsin B exhibit the same catalytic site, the enzyme papain was used in our study as the simulant of cathepsin B. Both of them are capable of specifically cleaving GFLG linker, as reported previously (*56*, *57*). However, HA-BPY-GEF-NPs could maintain the spherical morphology after incubated with papain for 12 hours, as revealed by TEM and DLS results (fig. S51, C and D). Under this circumstance, Ada-GFLG-GEF was also barely activated, which was attributed to the shielding effect of the outer Ada-BPY layer that protected the internal Ada-GFLG-GEF from being exposed to the papain in the surrounding environment so that the enzymatic cleavage ability of papain was hindered. These phenomena indicated that under the condition of only one stimulus input, either NIR irradiation or enzyme, HA-BPY-GEF-NPs cannot be efficiently activated to release GEF. In contrast, upon NIR photoirradiation for 10 min and subsequent incubation with papain for 12 hours, the HA-BPY-GEF-NPs exhibited an obvious morphology disruption since TEM results exhibited a loose and disintegrated structure (Fig. 2J) and DLS results indicated enlargement of nanoparticle size (fig. S52). Meanwhile, the characteristic GEF elution peak (t_R_ = 6.22 min) was detected by HPLC, accompanied by the gradual disappearance of the Ada-GFLG-GEF peak (t_R_ = 8.55 min) (Fig. 2H), suggesting the activation of Ada-GFLG-GEF component and GEF release. This was because the NIR illumination induced the generation of ROS, cleaving the ROS-sensitive thioketal bonds and leading to the breakdown of the nanoparticles. Consequently, the liberated Ada-GFLG-GEF components were exposed to papain and underwent a prodrug activation process. Therefore, the concept of sequential AND logic gate–based working mechanism of HA-BPY-GEF-NPs was verified that the active GEF could only be released when both NIR and enzyme stimuli were applied in the predefined order.

To further simulate the real situation of HA-BPY-GEF-NPs after entering into tumor cells, HA-BPY-GEF-NPs were incubated in mild acidic solution (pH 5.4) since the cathepsin B was localized in endosomes and lysosomes and was more active in these acidic milieus (*28*). As shown in Fig. 2I, the GEF was released more rapidly under the condition of double input of laser and papain in pH 5.4 medium, in which almost 100% of GEF was released from the HA-BPY-GEF-NPs within 6 hours. This process was faster than the GEF release speed under the pH 7.4 condition. According to previous research (*60*), the maximum activity of papain appears to be in mild acid condition, resulting in this faster drug release performance. Collectively, these results demonstrated that HA-BPY-GEF-NPs exhibited excellent AND molecular logic activation performance. When this supramolecular therapeutic nanoplatform was incubated with a single input (input laser or enzyme only), only a minimal amount of prodrug was activated, and GEF was hardly released. However, when incubated with both inputs in a predefined sequence (laser irradiation followed by papain incubation), a large amount of prodrug was activated, effectively releasing the GEF.

There are several established molecular AND logic platform models for designing activatable prodrugs, including dual functionalization (*40*), parallel activation (*61*), and sequential activation (*62*) (Fig. 2K). The dual-functionalization strategy, which relies on two distinct active sites, often results from a complex synthesis process and can lead to structural instability. To counteract this drawback, a design was crafted to activate two triggers at a single active site. While this approach simplifies the synthesis process and enhances structural stability, it presents a greater challenge in achieving heightened selectivity and precision within the intricate pathological context. In this work, we developed a kind of AND logic gate–based drug delivery system. This system integrates both internal and external stimuli–responsive conditions with a preset sequence activation that can improve the precision of controlled drug release for enhancing therapeutic precision. This system is different from the abovementioned AND logic–based systems, in which the GEF prodrug Ada-GFLG-GEF had only one activation site, which was the GFLG linker that responded to the cathepsin B. Another reactivation site was located on the HA scaffold, which was the thioketal linker that could be cleaved in response to ROS induced by PDT. Under the input of the first stimulation signal, that was, the application of external NIR illumination, Ada-BPY generated ROS through the photodynamic process, cutting off the thioketal linkers on the HA skeleton, and disintegrating the nanoparticles. Although cathepsin B was always present in the surroundings, it is only when the thioketal linkers were cleaved that the released Ada-GFLG-GEF components can contact with cathepsin B to cleave the GFLG linkers, releasing GEF. This process can be regarded as the input of the second stimulus signal. Only after the two stimulus signals were input in specified order that the AND logic gate can be effectively activated to release GEF. This combination of spatial location and double locking strategy ensures precise and controllable drug release to improve the spatiotemporal specificity of treatment, thus enhancing therapeutic efficacy and reducing side effects.

### Cellular uptake of HA-BPY-GEF-NPs

**Fig. 3.**
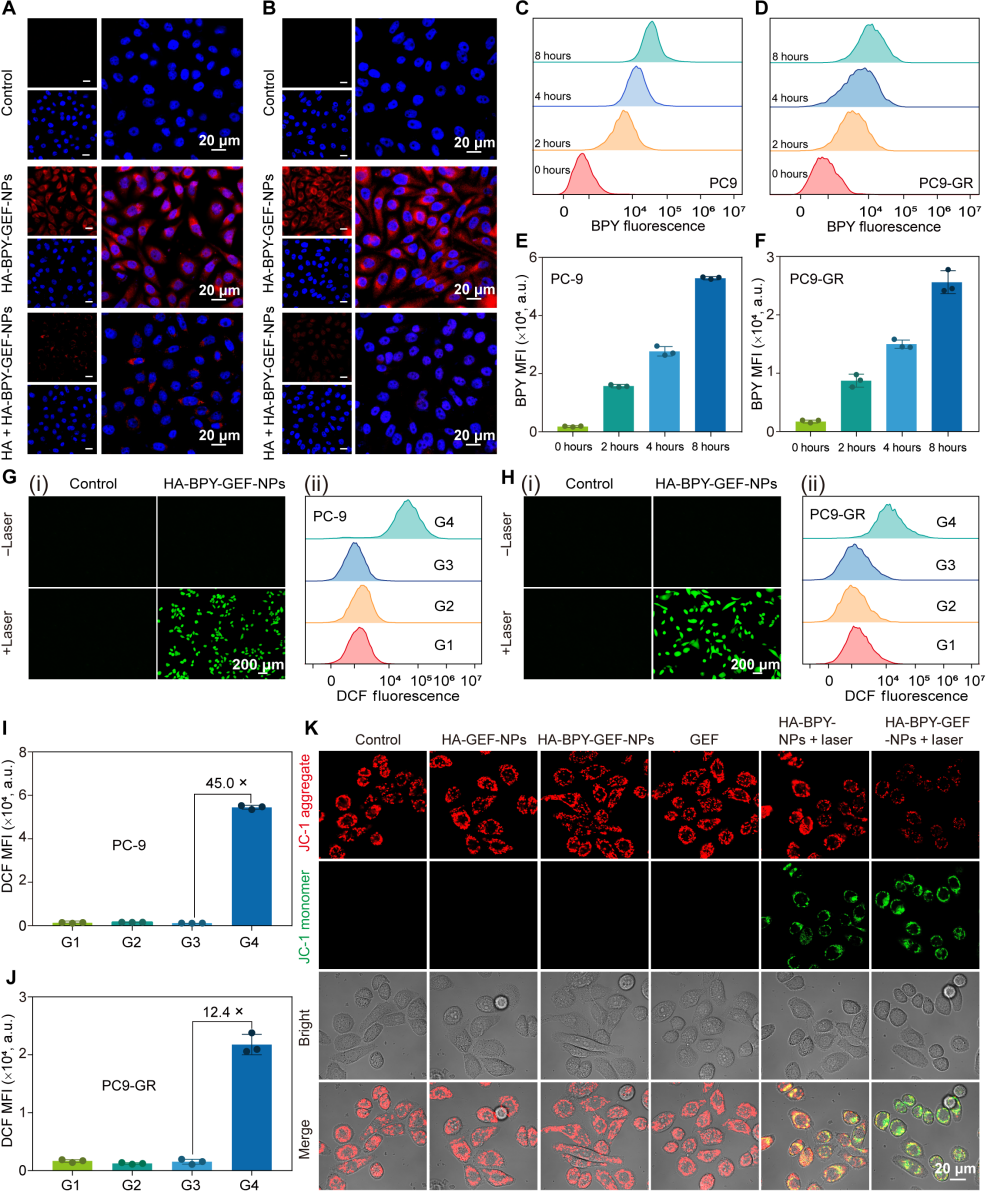
Cellular uptake, ROS generation, and mitochondrial membrane potential evaluation. CLSM images of (**A**) PC-9 and (**B**) PC9-GR cells incubated with HA-BPY-GEF-NPs for 4 hours with or without free HA pretreatment. FCM assessment of (**C**) PC-9 and (**D**) PC9-GR cell uptake behaviors of HA-BPY-GEF-NPs in a time-dependent manner. MFI of cellular uptake studies incubated with HA-BPY-GEF-NPs for different time on (**E**) PC9 and (**F**) PC9-GR cells. Data are shown as means ± SD (*n* = 3). Intracellular ROS detection in (**G**) PC-9 cells and (**H**) PC9-GR cells: (**I**) representative fluorescence images of intracellular ROS generation detected by a DCFH-DA probe and (ii) FCM analysis of intracellular DCF fluorescence. MFI of intracellular DCF fluorescence in (i) PC-9 and (**J**) PC9-GR cells. G1 to G4 refer to control, control + laser, HA-BPY-GEF-NPs, and HA-BPY-GEF-NPs + laser, respectively. Data are shown as means ± SD (*n* = 3). (**K**) CLSM images of changes in mitochondrial membrane potential of PC9-GR cells after treatment with different conditions. a.u., arbitrary units.

HA has been widely used as a drug delivery vehicle because it could specifically recognize overexpressed CD44 receptors on surface of tumor cells (*63*). Therefore, CD44 receptor–mediated endocytosis of the nanoparticles was first investigated by confocal laser scanning microscope (CLSM). As shown in Fig. 3 (A and B), after incubating with HA-BPY-GEF-NPs for 4 hours, obvious strong red fluorescence was observed both in PC-9 and PC9-GR cells, suggesting successful cellular uptake of the nanoparticles. In contrast, when PC-9 and PC9-GR cells were pretreated with extra free HA for 2 hours, only weak red fluorescence was observed in CLSM images (Fig. 3, A and B), indicating insufficient nanoparticle uptake due to the occupation of CD44 receptors by free HA, which hindered the CD44 receptor–mediated endocytosis of HA-BPY-GEF-NPs. Furthermore, time-dependent cellular uptake profiles of HA-BPY-GEF-NPs in PC-9 and PC9-GR cells were evaluated by flow cytometry (FCM) and CLSM. As displayed in Fig. 3 (C to F), the fluorescence intensity signals of Ada-BPY in HA-BPY-GEF-NPs, monitored and quantified through FCM, exhibited a gradually increasing trend with extended incubation time (from 0 to 8 hours), which indicated that HA-BPY-GEF-NPs could be effectively internalized by PC-9 and PC9-GR cells. Meanwhile, the CLSM images also showed a gradual enhancement of red fluorescence signals in the cytoplasm with prolonged treatment time (fig. S53), which was in accordance with the FCM results described above. These collective findings proved an efficient cellular uptake of HA-BPY-GEF-NPs.

### ROS generation efficacy and mitochondrial membrane potential disruption performance of HA-BPY-GEF-NPs

According to our design, the ROS generated from the photodynamic process played three crucial roles in suppressing GEF-resistant tumors. First, ROS as a cytotoxic agent could damage cell components such as membrane lipid and DNA, directly resulting in cell apoptosis. Second, ROS induces the cleavage of thioketal bonds that causes nanoparticle disassembly and facilitates subsequent prodrug activation. Last, the enhanced ROS level in cells inhibits the bypass signaling pathway activation to overcome GEF resistance. After confirming that HA-BPY-NPs could produce ROS under 660-nm light irradiation in solution (Fig. 2F and fig. S50), we proceeded to validate whether the internalized HA-BPY-GEF-NPs could generate ROS through photodynamic process in vitro. The fluorescent probe 2′,7′-dicholorofluorescin diacetate (DCFH-DA) was used to detect the intracellular ROS levels. As shown in Fig. 3 (G and H), in both PC-9 and PC9-GR cells, the intracellular green fluorescence was not visible for the blank group with and without light irradiation and the nonirradiated HA-BPY-GEF-NPs group, suggesting minimal generation of ROS. In comparison, strong green fluorescence was observed in the HA-BPY-GEF-NPs + laser group, validating the ROS generation ability of HA-BPY-GEF-NPs at the cellular level. The FCM results were consistent with the fluorescent imaging results (Fig. 3, G and H). Quantitatively, the mean fluorescence intensity (MFI) of DCF in PC-9 and PC9-GR cells treated with HA-BPY-GEF-NPs increased approximately 45.0- and 12.4-fold, respectively, when exposed to light irradiation compared to the nonirradiated group (Fig. 3, I and J). The ROS produced by photodynamic process could cause damage to mitochondrial membrane (*64*). As ROS levels were elevated by HA-BPY-GEF-NPs + laser treatment, the mitochondrial membrane potential changes were assessed by JC-1 assay. As shown in Fig. 3K, for the HA-BPY-NPs + laser group and the HA-BPY-GEF-NPs + laser group, green fluorescence was observed, accompanied by a decreased red fluorescence intensity, suggesting mitochondria depolarization during PDT. On the contrary, the nonirradiated groups did not exhibit such phenomenon.

### Design of fluorescence nanoprobe HA-BPY-NAP-NPs for visualizing AND logic gate–based controlled release behavior

Because of the lack of fluorescence signal of Ada-GFLG-GEF, the controllable release behavior of HA-BPY-GEF-NPs in solution based on sequential AND logic gate could only be verified by HPLC. To further investigate the AND logic gate–based working mechanism of HA-BPY-GEF-NPs at the cellular level and visualize the intracellular drug release, we designed a naphthalimide-based fluorescent nanoprobe, named as HA-BPY-NAP-NPs. Naphthalimide derivatives are usually used as cellular imaging agents due to its excellent photophysical characteristics (*65*). The HA-BPY-NAP-NPs nanoprobe shares a similar composition with HA-BPY-GEF-NPs, with the exception of GEF component that was replaced by naphthalimide. The synthesis details are provided in the Supplementary Materials (fig. S5). The key intermediates and the final product Ada-GFLG-NAP were fully characterized by NMR and HR-MS (figs. S35 to S43). The preparation process of HA-BPY-NAP-NPs was similar to that of HA-BPY-GEF-NPs. First, HA-TK-CD and Ada-GFLG-NAP were assembled through supramolecular host-guest interaction to obtain the primary nanoparticle structure. Subsequently, Ada-BPY was introduced and bound to the outer β-CD sites.

**Fig. 4.**
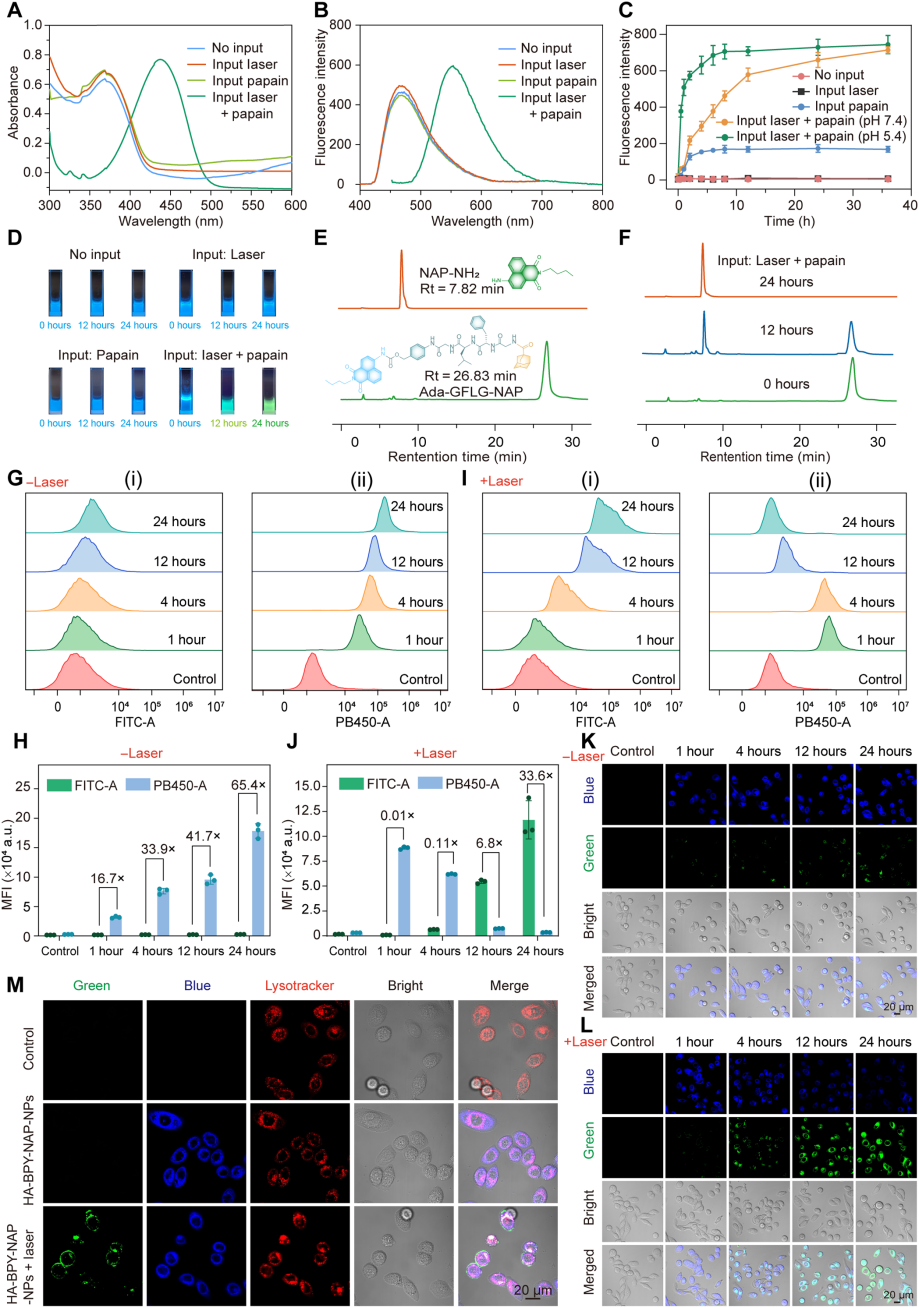
Design and characterization of fluorescence nanoprobe HA-BPY-NAP-NPs for visualizing AND logic gate mechanism. (**A**) UV-vis absorption spectra and (**B**) fluorescence spectra of HA-BPY-NAP-NPs nanoprobe under different conditions after incubation for 24 hours. (**C**) Fluorescence intensity variations of HA-BPY-NAP-NPs nanoprobe at 435 nm under different conditions (*n* = 3). (**D**) Photographs of HA-BPY-NAP-NPs nanoprobe under 365-nm UV lamp after different treatments at 0, 12, and 24 hours, respectively. (**E**) HPLC profiles of Ada-GFLG-NAP and NAP-NH_2_. (**F**) HPLC profiles of HA-BPY-NAP-NPs nanoprobe after laser irradiation (660 nm, 100 mW/cm^2^, 10 min) followed by further incubation with papain (10 μg/ml) for 0,12, and 24 hours, respectively. (**G**) FCM analysis results of PC9-GR cells treated with HA-BPY-NAP-NPs nanoprobe (10 μM) at different time in (i) FITC-A (green fluorescence, λ_ex_ = 488 nm) channel and (ii) PB450-A (blue fluorescence, λ_ex_ = 405 nm) channel. (**H**) Corresponding FCM quantification analysis results of MFI in (G). (**I**) FCM analysis results of PC9-GR cells treated with HA-BPY-NAP-NPs nanoprobe (10 μM) and light irradiation (660 nm, 100 mW/cm^2^, 10 min) at different time in (i) FITC-A (green fluorescence, λ_ex_ = 488 nm) channel and (ii) PB450-A (blue fluorescence, λ_ex_ = 405 nm) channel. (**J**) Corresponding FCM quantification analysis results of MFI in (I). Data are shown as means ± SD (*n* = 3). (**K** and **L**) CLSM images of PC9-GR cells treated with HA-BPY-NAP-NPs nanoprobe (10 μM) for different time, with or without NIR photoirradiation (660 nm, 100 mW/cm^2^) for 10 min. Green channel, λ_ex_ = 488 nm and blue channel, λ_ex_ = 405 nm. (**M**) CLSM images of PC9-GR cells after the coincubation of Lysotracker and HA-BPY-NAP-NPs nanoprobe (10 μM). Green channel, λ_ex_ = 488 nm; red channel, λ_ex_ = 561 nm; and blue channel, λ_ex_ = 405 nm.

For HA-BPY-NAP-NPs nanoprobe, the UV-vis absorption spectrum exhibited a characteristic peak at 375 nm and the fluorescence spectrum showed a characteristic emission peak at 480 nm (λ_ex_ = 375 nm), which were similar to Ada-GFLG-NAP (figs. S54A and S55A). As shown in Fig. 4A and fig. S54 (B to D), the absorption peaks of HA-BPY-NAP-NPs in the UV-vis absorption spectrum remained unchanged both after exposure to 660 nm of light or after incubation with papain for 24 hours. In contrast, in the presence of the sequential dual input (laser + papain), the absorption peak distinctly red-shifted from 375 to 435 nm in the absorption spectrum of HA-BPY-NAP-NPs (Fig. 4A and fig. S54E). In the fluorescence spectrum of HA-BPY-NAP-NPs, an emission peak at 480 nm was detected with the excitation wavelength at 375 nm (fig. S54A). When HA-BPY-NAP-NPs were excited by light of wavelength 435 nm, only a weak fluorescence emission was observed (fig. S55B). Under the treatment of a single stimulus (either 660-nm laser irradiation or papain coincubation), the fluorescence emission intensity of HA-BPY-NAP-NPs when excited at 435 nm was also very weak (fig. S55, C and D). After the input of dual stimuli that complied with the AND logic gate, HA-BPY-NAP-NPs exhibited a fluorescence emission signal at 550 nm (Fig. 4B and fig. S55E). When the experimental conditions were further adjusted to weakly acidic conditions (pH 5.4), the rate of increase in fluorescence emission intensity at 550 nm became faster (Fig. 4C and fig. S55F).

The fluorescence spectrum of HA-BPY-NAP-NPs after sequential input of laser and papain dual stimuli was consistent with the characteristic fluorescence of NAP-NH_2_ (fig. S55a), which indicated that the naphthalimide probes from HA-BPY-NAP-NPs were released. This release behavior could also be directly visualized under a 365-nm UV lamp (Fig. 4D). In the presence of sequential laser + papain stimuli, the HA-BPY-NAP-NPs solution exhibited a gradual transition from blue to green fluorescence within 24 hours. However, for the control group without any input and the single input groups, the fluorescence of HA-BPY-NAP-NPs solution remained blue throughout the entire duration of the experiment. The AND logic–based activation profiles and the release of NAP-NH_2_ from the disassembly of HA-BPY-NAP-NPs was also investigated by HPLC. As shown in Fig. 4 (E and F), the HPLC results showed a new elution peak (t_R_ = 7.82 min) along with the disappearance of Ada-GFLG-NAP in HA-BPY-NAP-NPs nanoprobe (t_R_ = 26.83 min) within 24 hours under the condition of sequential input of laser and papain. These observations demonstrate the successful release of NAP-NH_2_, while the other three groups exhibited no obvious change (fig. S56). The above results collectively confirmed that the fluorescent naphthalimide probe NAP-NH_2_ could be effectively released under sequential input of laser and papain, and the release rate became faster under weakly acidic conditions.

Considering the complex characteristics of tumor microenvironment, such as acidity, high redox levels, and overexpressed ATP. The selectivity of HA-BPY-NAP-NPs nanoprobe was subsequently investigated. As shown in fig. S57, without input or only one stimulus input (NIR irradiation alone or enzyme alone), no noticeable fluorescence change was observed in the case of any of these potential interferants. By contrast, in the presence of the sequential dual input (laser + papain), HA-BPY-NAP-NPs exhibited an obvious fluorescence emission signal at 550 nm (fig. S57C). Collectively, these results confirmed that the nanoprobe has a superior specificity.

### The responsiveness and intracellular distribution of the nanoprobe HA-BPY-NAP-NPs

To investigate the cellular imaging capacity and intracellular distribution of HA-BPY-NAP-NPs, PC9-GR cells were chosen as the cell model. In the absence of 660-nm laser irradiation, the FCM results (Fig. 4G) of HA-BPY-NAP-NP–treated PC9-GR cells exhibited a weak green fluorescence signal, while the blue fluorescence increased gradually. According to the quantitative analysis results, after 24 hours of coincubation, the MFI in the blue PB 450 channel was 65.4-fold higher than MFI in green fluorescein isothiocyanate (FITC) channel (Fig. 4H). These results indicate an efficient cellular uptake of HA-BPY-NAP-NPs by PC9-GR cells; however, they were not activated. On the contrary, as shown in Fig. 4I, upon light irradiation, the blue fluorescence gradually decreased along with an increase in green fluorescence as the incubation time progressed. After 24 hours, the MFI in FITC channel (green fluorescence) was 33.6-fold higher than the MFI in PB 450 channel (blue fluorescence) (Fig. 4J). The CLSM images also reflected this trend. Without light irradiation, HA-BPY-NAP-NPs emitted only a very weak green fluorescence in PC9-GR cells (Fig. 4K). Upon exposure to 660-nm light irradiation, HA-BPY-NAP-NPs showed a pronounced green fluorescence in PC9-GR cells, coincident with a reduction in the original blue fluorescence (Fig. 4L), suggesting that the HA-BPY-NAP-NPs nanoprobe was successfully activated in PC9-GR cells. The subcellular localization of HA-BPY-NAP-NPs was further confirmed by commercial lysosomal probe (Lyso-Tracker). By incubating PC9-GR cells with the HA-BPY-NAP-NPs and Lyso-Tracker, the green and blue fluorescence of HA-BPY-NAP-NPs after laser irradiation overlapped well with the red fluorescence of Lyso-Tracker (Fig. 4M), indicating that the HA-BPY-NAP-NPs were distributed in lysosomes after internalization and could be effectively activated within the lysosomes.

### In vitro therapeutic effect evaluation of HA-BPY-GEF-NPs

**Fig. 5.**
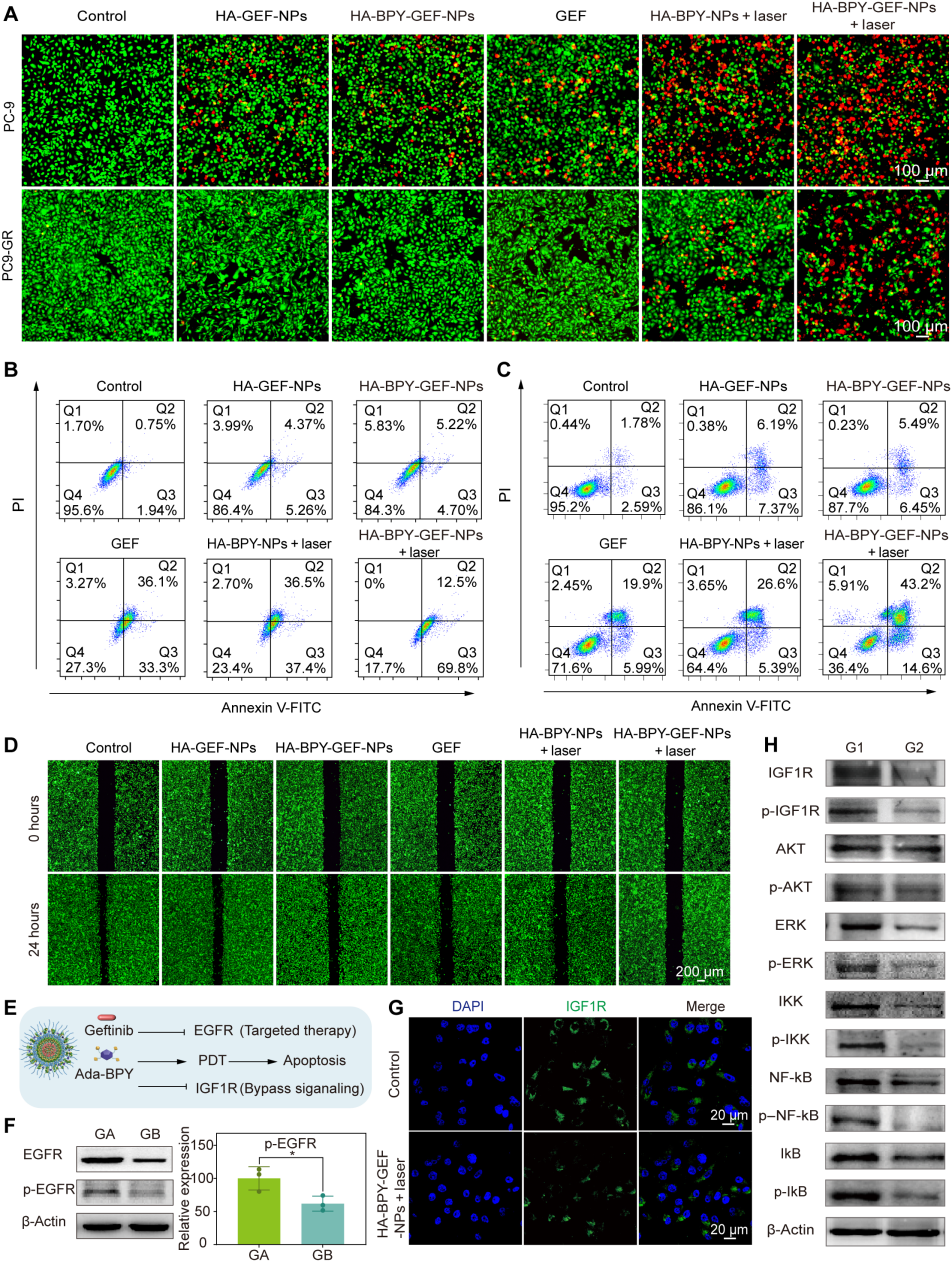
In vitro antitumor activities and GEF-resistance overcoming mechanisms of HA-BPY-GEF-NPs. (**A**) Fluorescence images of Calcein-AM/PI double-stained PC9 and PC9-GR cells after different treatments. Apoptosis analysis of (**B**) PC-9 and (**C**) PC9-GR cells after different treatments by FCM. (**D**) Fluorescence images of wound healing assay of PC9-GR cells incubated with different formulations. (**E**) Schematic illustration of the combination therapy using HA-BPY-GEF-NPs for overcoming GEF resistance. (**F**) Western blot images of EGFR and p-EGFR protein expression in PC9-GR cells after different treatments. Quantitative analysis of relative expression levels of p-EGFR. GA, HA-BPY-GEF-NPs; GB, HA-BPY-GEF-NPs + laser. Data are shown as means ± SD (*n* = 3), *P < 0.05. (**G**) Representative immunofluorescence images of IGF1R in PC9-GR cells. (**H**) Representative Western blot images of IGF1R, p-IGF1R, AKT, p-AKT, ERK, p-ERK, IKK, p-IKK, NF-κB, p–NF-κB, IκB, and p-IκB protein expression in PC9-GR cells after different treatments. G1, Control; G2, HA-BPY-GEF-NPs + laser (the complete results of the three replicate experiments are displayed in fig. S60).

According to the Calcein-AM/propidium iodide (PI) double-staining results (Fig. 5A), for HA-GEF-NPs and HA-BPY-GEF-NPs groups in PC-9 and PC9-GR cells, strong green fluorescence (indicative of live cells) and negligible red fluorescence (indicative of dead cells) were observed, suggesting the reduction of cytotoxicity via blocking the active site of GEF before activation. By contrast, HA-BPY-GEF-NPs + laser group exhibited strongest fluorescence of PI (indicative of dead cells) followed by HA-BPY-NPs + laser and free GEF both in PC-9 and PC9-GR cells. Furthermore, the apoptosis of the tumor cells after different treatments was investigated by FCM. As shown in Fig. 5 (B and C), the HA-BPY-GEF-NPs + laser groups exhibited the highest apoptosis ratios (82.3% for PC-9 and 57.8% for PC9-GR), which were 8.29-fold and 4.8-fold higher than those of HA-BPY-GEF-NPs in PC-9 and PC9-GR, respectively. These results further indicate that HA-BPY-GEF-NPs combined with laser irradiation could overcome the problem of GEF resistance. As shown in Fig. 5D and the quantitative statistical analysis in fig. S58, the wound healing assay results also showed that HA-BPY-GEF-NPs + laser treatment exhibited the lowest cell motility rate at 24 hours after administration compared to the other groups, indicating that HA-BPY-GEF-NPs with laser irradiation showed most effective antimetastasis ability.

### Research on overcoming GEF resistance mechanisms

The above studies have confirmed that HA-BPY-GEF-NPs combined with light irradiation exhibited the strongest cytotoxicity toward the GEF-resistant PC9-GR cells. According to our design, upon activation by the AND logic gate, HA-BPY-GEF-NPs releases GEF, which inhibits EGFR expression and blocks related signaling pathways, thereby suppressing the growth and metastasis of tumor cells. Simultaneously, the Ada-BPY component generated cytotoxic ROS through photodynamic process, directly inhibiting tumor cell growth and disrupting IGF1R bypass signal activation (Fig. 5E). To investigate how HA-BPY-GEF-NPs overcome the GEF resistance mechanism in PC9-GR cells, the EGFR expression level and its phosphorylated form (p-EGFR) were first evaluated via Western blot analysis. According to the results shown in Fig. 5F, it could be observed that EGFR and p-EGFR were suppressed in the HA-BPY-GEF-NPs + laser group, compared to the group without laser irradiation (fig. S59). This result indicated that the ROS generated from HA-BPY-GEF-NPs + laser treatment effectively inhibited the EGFR signaling pathway, making it potentially useful in treating EGFR-TKI–resistant NSCLC.

Previous studies have demonstrated the crucial role of IGF1R bypass signaling in modulating EGFR-TKI resistance (*66*). Fortunately, elevating ROS levels could inhibit IGF1R bypass signaling and ERK/AKT/nuclear factor κB (NF-κB) signal pathways in the downstream, leading to high efficacy in inhibiting cell proliferation and overcoming bypass signal activation–related GEF resistance in NSCLC treatment (*13*). Therefore, we investigated the expression of IGF1R after administering HA-BPY-GEF-NPs in combination with laser irradiation. Immunofluorescent imaging results (Fig. 5G) confirmed the reduced IGF1R expression level in HA-BPY-GEF-NPs + laser–treated PC9-GR cells compared to the control group, indicating down-regulation of IGF1R activity upon HA-BPY-GEF-NPs + laser treatment. Moreover, Western blot results further confirmed notably lower levels of IGF1R and phosphorylated IGF1R in PC9-GR cells after administering HA-BPY-GEF-NPs combined with laser irradiation (Fig. 5H and figs. S60 and S61).

Subsequently, we evaluated the downstream signals of IGF1R after HA-BPY-GEF-NPs + laser treatment. The PI3K/AKT and MAPK/ERK pathways are two major signaling pathways involved in the downstream activation of IGF1R, with AKT and ERK serving as crucial proteins for the PI3K/AKT and MAPK/ERK pathways, respectively. AKT and ERK in phosphorylated forms indicate the activation of the signal pathways, respectively. Notably, after HA-BPY-GEF-NPs + laser treatment, the expression levels of phosphorylated AKT and ERK decreased compared to the control group. This result suggested that HA-BPY-GEF-NPs + laser treatment down-regulated PI3K/AKT and MAPK/ERK signaling pathways in PC9-GR cells. Furthermore, the NF-κB pathway, another downstream signaling pathway of PI3K/AKT and MAPK/ERK, was also involved in IGF1R bypass signaling activation. IKK, IκB, and NF-κB represented the three core proteins within NF-κB pathway. As shown in Fig. 5H and figs. S60 and S61, compared to the control group, the expression levels of the phosphorylated forms, including p-IKK, p-IκB, and p–NF-κB, were remarkably reduced after HA-BPY-GEF-NPs + laser treatment, suggesting the inactivation of NF-κB signaling pathway. Collectively, the active GEF drug released from HA-BPY-GEF-NPs + laser group inhibited the EGFR signaling pathway, thus potentially enhancing the responsiveness of drug-resistant cells to TKIs. In addition, the ROS induced by HA-BPY-GEF-NPs + laser treatment not only facilitated PDT but also down-regulated IGF1R expression and subsequently inactivated the MAPK/ERK, PI3K/AKT, and NF-κB signaling pathways. These results confirmed the excellent efficacy of NIR laser–irradiated HA-BPY-GEF-NPs in overcoming GEF resistance in NSCLC cell line.

### Biodistribution and in vivo antitumor performance of HA-BPY-GEF-NPs in PC-9 tumor–bearing mouse models

**Fig. 6.**
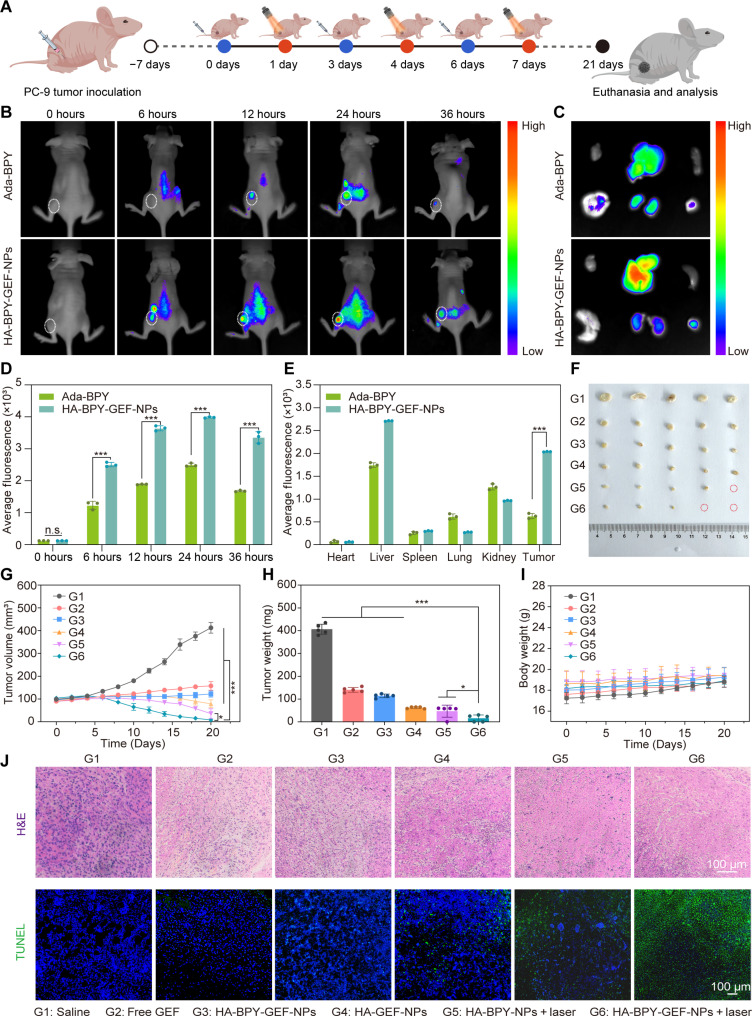
In vivo biodistribution and antitumor performance of HA-BPY-GEF-NPs in the mouse models bearing GEF-sensitive PC-9 tumors. (**A**) Schematic illustration of treatment schedules. (**B**) Time-dependent in vivo fluorescence imaging results of PC-9 tumor–bearing mice intravenously injected with Ada-BPY or HA-BPY-GEF-NPs. Dashed circles indicated the locations of the tumors. (**C**) Ex vivo fluorescence images of major organs and tumor tissues taken at 36 hours after intravenous injection of Ada-BPY or HA-BPY-GEF-NPs. (**D**) MFI quantitative analysis of tumor tissues after intravenous injection with Ada-BPY or HA-BPY-GEF-NPs at different time points (*n* = 3). (**E**) Quantification of MFI in tumor tissues and major normal organs, which were collected at 36 hours after injection (*n* = 3). (**F**) Photograph of tumor tissues collected at the end of the experiment from different groups. G1, Saline; G2, free GEF; G3, HA-BPY-GEF-NPs; G4, HA-GEF-NPs; G5, HA-BPY-NPs + laser; and G6, HA-BPY-GEF-NPs + laser. (**G**) Tumor growth profiles of mice in different groups (*n* = 5). (**H**) Body weight changes of mice in different groups with different treatments over a period of 20 days (*n* = 5). (**I**) Weights of tumor tissues in different groups at the end of the experiment (*n* = 5). (**J**) H&E and TUNEL staining of dissected tumor tissues in different groups. Data are shown as means ± SD. *P < 0.05 and ***P < 0.001. n.s., not significant.

Inspired by the remarkable in vitro antitumor performance of HA-BPY-GEF-NPs, we further investigated the in vivo tumor inhibitory capabilities of HA-BPY-GEF-NPs in PC-9 tumor–bearing mice (Fig. 6A). The effective accumulation of nano-drug formulations at the tumor site is a prerequisite for achieving effective therapeutic outcomes. Therefore, the biodistribution of HA-BPY-GEF-NPs was monitored via in vivo imaging system after intravenous administration in xenograft models. As shown in Fig. 6B, for HA-BPY-GEF-NPs group, the fluorescence intensity in the tumor region gradually increased after intravenous injection. In contrast, the Ada-BPY group exhibited negligible fluorescence signal at the tumor site within 36 hours after injection. These results suggested that compared to free Ada-BPY, HA-BPY-GEF-NPs in nanoformulation form exhibited a greater tendency to accumulate at the tumor site. In addition, ex vivo fluorescence imaging also indicated that the fluorescence signal in tumor tissue was considerably stronger in the HA-BPY-GEF-NPs group compared to the free Ada-BPY group (Fig. 6C). The fluorescence signal in HA-BPY-GEF-NPs group reached a peak at 24 hours after injection, indicating that this time point was the optimal time for applying light stimulation to induce PDT (Fig. 6D). According to quantitative analysis, the fluorescence intensity at the tumor region in the HA-BPY-GEF-NPs group was 3.25-fold higher than that in the free Ada-BPY group (Fig. 6E). This enhanced accumulation performance may be attributed to the synergistic effects of the optimal nanoparticle size, which facilitated the enhanced permeability and retention (EPR) effect, along with the active internalization of HA-based nanoparticles through CD44 receptors on tumor cells.

Subsequently, the therapeutic effectiveness against PC-9 tumor growth was evaluated, following the treatment schedule depicted in Fig. 6A. When the tumor volume reached approximately 100 mm^3^, the mice were randomly divided into six groups to receive different treatments. All groups were administered with intravenous injections of various formulations every 3 days at an equivalent dose of 3.2 mg of GEF per kilogram of the mice body weight. In addition, for laser-irradiated groups, the tumors were further subjected to 660-nm NIR laser irradiation (200 mW/cm^2^, 10 min) at 24 hours after administration, when drug accumulation in tumors reached its highest levels. As shown in Fig. 6 (F and G), the tumor volume grew rapidly in the saline group, while for other experimental groups, the tumor growth profiles were inhibited to different degrees. Compared with the GEF monotherapy group and the PDT monotherapy group, the combined treatment (HA-BPY-GEF-NPs + laser) exhibited the best tumor inhibitory effect, with the smallest average tumor size at the end of the experiment. The tumor growth inhibition rate in mice treated with HA-BPY-GEF-NPs + laser was measured to be 98.03% on day 20 (fig. S62).

During the entire treatment period, no noticeable body weight loss was observed in all the groups (Fig. 6H), suggesting good biosafety of the therapeutic nanoplatform. The tumors in each group were collected and weighed (Fig. 6I). It was found that the average weight of tumors in the mice treated with HA-BPY-GEF-NPs + laser was also the smallest compared to all the other groups. The average tumor weight was 15.8 ± 14.96 mg, which was only 4% of the weight observed in mice treated with saline. Moreover, the serum biochemistry analysis results revealed no obvious abnormalities in the HA-BPY-GEF-NPs + laser group (fig. S63). Hematoxylin and eosin (H&E) staining results of major organs also showed no discernible damage (fig. S64). The in vivo antitumor efficiency was further investigated through H&E staining and terminal deoxynucleotidyl transferase–mediated deoxyuridine triphosphate nick end labeling (TUNEL) staining (Fig. 6J). The HA-BPY-GEF-NPs + laser group exhibited the most prominent nuclear shrinkage and fragmentation. Similar trends were observed in the TUNEL staining images where the highest degree of cellular apoptosis was observed in the HA-BPY-GEF-NPs + laser group, which also had the most effective tumor inhibition performance. This outcome could be attributed to the PDT along with cascaded prodrug activation. These results clearly demonstrate that the HA-BPY-GEF-NPs + laser treatment exhibited superior antitumor therapeutic efficacy compared to other treatments. Furthermore, this nanoplatform also exhibited exceptional biocompatibility and safety profiles.

### Biodistribution and in vivo antitumor activity in PC9-GR tumor–bearing models

**Fig. 7.**
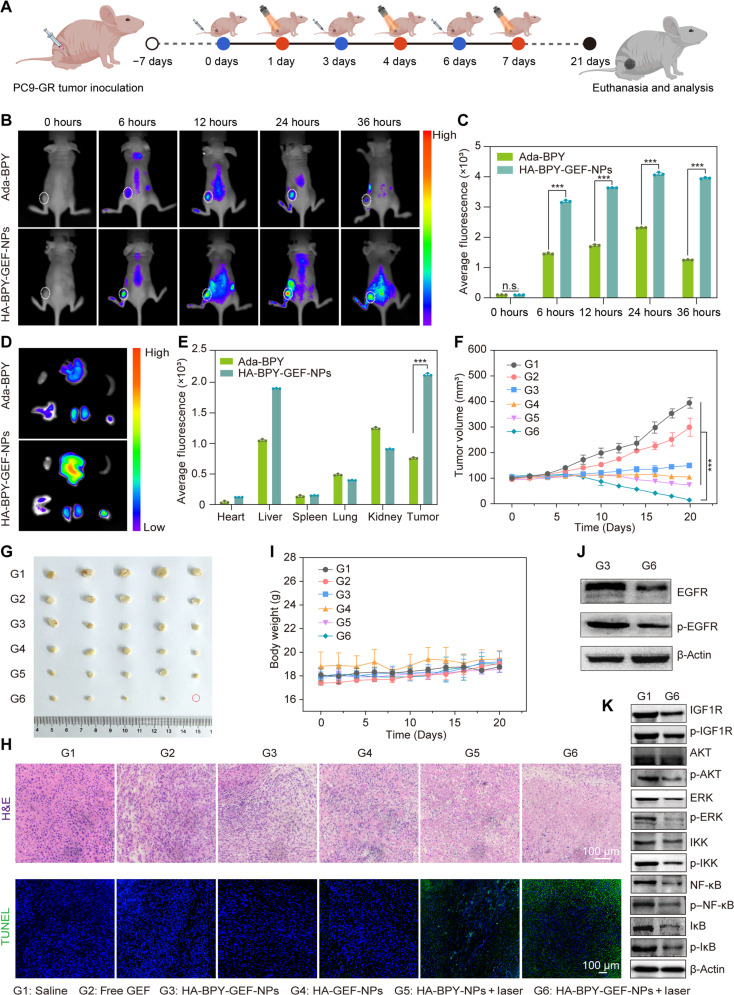
Biodistribution, antitumor therapeutic effect and antidrug resistance mechanisms of HA-BPY-GEF-NPs in the PC9-GR tumor–bearing mouse models. (**A**) Schematic illustration of treatment schedules. (**B**) Representative in vivo fluorescence images of PC9-GR tumor–bearing mice after intravenous injection of Ada-BPY or HA-BPY-GEF-NPs. Dashed circles indicated the locations of the tumors. (**C**) MFI quantitative analysis of tumor tissues after intravenous injection with Ada-BPY or HA-BPY-GEF-NPs at different time points (*n* = 3). (**D**) Ex vivo fluorescence images of major organs and tumor tissues taken at 36 hours after intravenous injection of Ada-BPY or HA-BPY-GEF-NPs. (**E**) MFI quantitative analysis of tumor tissues and major organs collected at 36 hours after administration (*n* = 3). (**F**) Tumor growth profiles of mice in different groups (*n* = 5). (**G**) Photograph of tumor tissues collected at the end of the experiment from different groups. (**H**) H&E and TUNEL staining of dissected tumor tissues in different groups. (**I**) Body weight changes of mice in different groups with different treatments over a period of 20 days (*n* = 5). G1, Saline; G2, free GEF; G3, HA-BPY-GEF-NPs; G4, HA-GEF-NPs; G5, HA-BPY-NPs + laser; and G6, HA-BPY-GEF-NPs + laser. (**J**) Western blot images of EGFR, p-EGFR, and β-actin in tumor tissues after treatment with HA-BPY-GEF-NPs (G3) and HA-BPY-GEF-NPs + laser (G6). (**K**) Western blot images of IGF1R, p-IGF1R, AKT, p-AKT, ERK, p-ERK, IKK, p-IKK, NF-κB, p–NF-κB, IκB, and p-IκB protein expression in tumor tissues after treatment with Saline (G1) and HA-BPY-GEF-NPs + laser (G6). Data are shown as means ± SD. ***P < 0.001.

Encouraged by the superior therapeutic effects of HA-BPY-GEF-NPs + laser treatment in PC-9 tumor–bearing nude mice, we further evaluated the in vivo antitumor effects of HA-BPY-GEF-NPs in GEF-resistant PC9-GR tumor–bearing mice (Fig. 7A). The tumor accumulation capability of HA-BPY-GEF-NPs was first evaluated via a small animal fluorescence imaging system. As shown in Fig. 7 (B and C), the fluorescence intensity of HA-BPY-GEF-NPs in the tumor region gradually increased following intravenous injection and reached its maximum at 24 hours after injection, similar to the mice bearing PC-9 tumors. Furthermore, for the HA-BPY-GEF-NP–treated mice, the fluorescence intensity in the tumor region remained at a high level even after 36 hours of injection. In comparison, only negligible fluorescence signal was detected in tumors from the group treated with free Ada-BPY, which suggested that HA-BPY-GEF-NPs exhibited higher accumulation levels in the tumor site compared to free Ada-BPY. The mice were euthanized at 36 hours after intravenous injection of Ada-BPY or HA-BPY-GEF-NPs. Major organs and tumor tissues were collected for ex vivo fluorescence imaging (Fig. 7D). The results also indicated that the HA-BPY-GEF-NPs group displayed markedly higher fluorescence signal intensity in the tumor region compared to the Ada-BPY group. On the basis of the semiquantitative analysis, it was found that the fluorescence intensity of HA-BPY-GEF-NPs at the tumor site was 2.8-fold higher than that of free Ada-BPY (Fig. 7E). These results collectively demonstrate that HA-BPY-GEF-NPs exhibited superior tumor targeting and accumulation performance than Ada-BPY, possibly attributed to the CD44 receptor–mediated active targeting ability and the EPR effect of the nanoformulations.

Next, we evaluated the therapeutic effect of different nanoformulations in PC9-GR tumor xenografted mice (Fig. 7A). When the tumor volumes reached about 100 mm^3^, the mice were treated with saline, free GEF, HA-BPY-GEF-NPs, HA-GEF-NPs, HA-BPY-NPs + laser, and HA-BPY-GEF-NPs + laser at an equivalent dose of 3.2 mg of GEF or 3.6 mg of Ada-BPY for every kilogram of body weight. Nanoformulations were administered three times at a time interval of 3 days, and light-irradiated groups were further treated with 660-nm NIR laser irradiation at days 1, 4, and 7, respectively. As shown in Fig. 7F, the free GEF treatment only resulted in a slight reduction in tumor growth, with an inhibition rate of 24.14% (fig. S65), suggesting strong GEF resistance of the PC9-GR tumors. In contrast, the tumor inhibition efficacy showed a modest improvement in the HA-BPY-GEF-NPs, HA-GEF-NPs, and HA-BPY-NPs + laser–treated groups. However, in these groups the GEF-resistant tumors still could not be effectively eliminated. The HA-BPY-GEF-NPs + laser group resulted in high tumor growth inhibition effect in PC9-GR tumors. Similar to the in vivo antitumor experiments with GEF-sensitive tumors, HA-BPY-GEF-NPs + laser treatment exhibited the highest tumor growth inhibition rate of 96.35% (fig. S65), indicating the enhanced antitumor efficacy compared to the other groups.

The mice in each group were euthanized after 20 days of treatment, and the tumors were excised. The images (Fig. 7G) and the weight (fig. S66) of the excised tumors further confirmed that HA-BPY-GEF-NPs + laser group exhibited the best therapeutic outcome. In addition, the histological analysis of tumor sections through H&E and TUNEL staining (Fig. 7H) also revealed that HA-BPY-GEF-NPs + laser treatment induced extensive tumor damage, as characterized by a high proportion of apoptotic and necrotic cells. Last, there were no distinct body weight changes (Fig. 7I) and no obvious pathological damage of the major organs in all the groups (fig. S67). At the same time, the normal values of biochemistry assays between HA-BPY-GEF-NPs + laser and saline group also indicated that HA-BPY-GEF-NPs presented well tolerant and excellent biocompatibility (fig. S68), which were favorable for future clinical application.

To explore the molecular mechanisms of the combination therapy on overcoming GEF resistance in vivo, PC9-GR tumor–bearing mice were treated with saline, HA-BPY-GEF-NPs, and HA-BPY-GEF-NPs + laser, respectively. Compared to the HA-BPY-GEF-NPs group, lower EGFR and p-EGFR expression levels in tumor tissues after HA-BPY-GEF-NPs + laser treatment were observed (Fig. 7J and fig. S69). Subsequently, we examined the expression levels of crucial signaling proteins associated with the IGF1R bypass signaling pathway, including IGF1R, ERK, AKT, IKK, NF-κB, IκB. As shown in Fig. 7K, as well as in figs. S70 and S71, HA-BPY-GEF-NPs combined with laser irradiation reduced the protein expression of their corresponding phosphorylated form, indicating down-regulation of IGF1R and subsequent inactivation of the MAPK/ERK, PI3K/AKT, and NF-κB signaling pathways. Collectively, these results along with the in vivo anti–PC9-GR tumor performance confirmed that HA-BPY-GEF-NPs + laser treatment could overcome GEF resistance by not only suppressing the EGFR signaling pathway but also down-regulating IGF1R and its downstream signal pathways through the generation of ROS.

## DISCUSSION

In this study, we adopted an AND Boolean logic gate strategy to develop a supramolecular therapeutic platform, HA-BPY-GEF-NPs. This platform can sequentially respond to NIR and overexpressed cathepsin B stimuli within tumor regions, enhancing spatiotemporally precise activation. The HA-BPY-GEF-NPs was constructed by stepwise assembly of HA-TK-CD, Ada-GFLG-GEF, and Ada-BPY. The GEF prodrug Ada-GFLG-GEF was encapsulated in the inner core of the nanoparticles, protected by an outer layer of Ada-BPY, and isolated from the external environment. After entering the tumor cells, HA-BPY-GEF-NPs remained in an active state within cathepsin B–abundant lysosomes. Upon external NIR light irradiation, HA-BPY-GEF-NPs generated ROS through a photodynamic process, which led to the disruption of thioketal bonds within the nanoparticles and further resulted in the disintegration of HA-BPY-GEF-NPs. At this point, Ada-GFLG-GEF interacted with cathepsin B and was subsequently activated to release the active form of GEF.

GEF could only be released after sequential input of NIR and cathepsin B stimuli, and the ROS produced during PDT promoted the apoptosis of tumor cells and inhibited the activation of the bypass IGF1R signaling pathway. HA-BPY-GEF-NPs demonstrated highly controlled drug activation properties, enhancing both drug release precision and treatment accuracy. In addition, a fluorescent probe HA-BPY-NAP-NPs was also synthesized to validate the activation mechanism of AND logic gate at cellular level. As a result, HA-BPY-GEF-NPs exhibited the best antitumor performance among all the experimental groups in both GEF-sensitive PC-9 tumor model and GEF-resistant PC9-GR tumor model. Overall, the innovative integration of PDT with EGFR-TKI resistance overcoming, through an AND logic gate mechanism for drug release, makes it a promising system for cancer treatment. We plan to subsequently construct realistic NSCLC animal models to further assess the therapeutic effects of HA-BPY-GEF-NPs and are in active discussions regarding their potential use in human therapy. Despite the limitations in tissue penetration by external light sources, the clinical application of PDT for cancer treatment has become increasingly sophisticated. Recognizing the limitations of light penetration in PDT, we are encouraged by advancements in optical fiber materials that can effectively deliver laser light to treatment areas, offering an advantage for the application of our nanoparticles, especially in minimally invasive endoscopic or interventional procedures.

## MATERIALS AND METHODS

### Preparation of HA-BPY-GEF-NPs and control samples

Briefly, host molecule (HA-TK-CD) was dissolved in ultrapure water to prepare a stock solution with the concentration of β-CD (1 mM). GEF prodrug (Ada-GFLG-GEF) and photosensitizer (Ada-BPY) as guest molecules were dissolved in dimethyl sulfoxide (DMSO) to obtain stock solutions (2 mM), respectively. The host polymer stock solution was diluted 20 folds to a concentration of 100 μM. Ada-GFLG-GEF was first injected into the host polymer solution slowly, followed by injection of Ada-BPY with different ratios under sonication over 5 min to obtain a colloidal solution. Then, the resulting solution was left to stand at room temperature overnight for sufficient assembly, followed by dialysis against water for 2 hours to remove DMSO trace. The prepared solution was stored at 4°C. The other nanoparticles were prepared by the same method. To construct the fluorescent nanoprobe HA-BPY-NAP-NPs, Ada-GFLG-GEF was substituted with Ada-GFLG-NAP, and then HA-BPY-NAP-NPs was prepared following the same methods as mentioned above for further study.

### Cell culture

GEF-sensitive PC-9 cells and GEF-resistant PC9-GR cells were cultured in DMEM complete medium, containing 10% FBS and 1% penicillin and streptomycin, and the cells were incubated at 37°C with an atmosphere containing 5% CO_2_.

### In vitro drug release

HPLC was applied to investigate the drug release behavior. Briefly, the nanoparticle solution was prepared in PBS with the concentration of 100 μM. Then, the samples were incubated with different conditions: PBS (no input), laser, papain, laser + papain (pH 7.4), and laser + papain (pH 5.4), respectively. All of the mixtures were incubated at 37°C. At different time points, the solution (100 μl) was drawn out and diluted with methanol to the concentration of 50 μM. Subsequently, the solution was examined by HPLC (mobile phase: A, CH_3_OH; B, H_2_O with 0.1% TEA; flow rate, 1.0 ml/min; column temperature, 25°C; UV, 254 nm; elute, VA:VB = 80:20).

### In vitro safety assay of HA-TK-CD

The safety of drug carrier was evaluated using CCK-8 kit. The tumor cells (PC-9 and PC9-GR cells) and nontumor cells (NIH-3T3 and HUVECs) were seeded in a 96-well plate with a density of 5000 cells per well and incubated for 24 hours. Then, the HA-TK-CD was added into the culture medium at different concentrations from 0 to 250 μg ml^−1^. After treatment for 24 and 48 hours, the culture medium was removed and a fresh one with CCK-8 (10%) was added. After being incubated for another 30 min, the absorbance at 450 nm was detected by a microplate reader (SpectraMax i3x, Molecular Devices, USA).

### Cellular uptake study and intracellular ROS generation study

To investigate CD44 receptor–mediated endocytosis of the nanoparticles, PC-9 and PC9-GR cells were seeded in six-well plates (1 × 10^5^ cells per well) and incubated at 37°C for 24 hours. Then, the cells were treated with free HA (10 mg/ml) for 2 hours or not. Next, cells were treated with HA-BPY-GEF-NPs (10 μM). After incubating for 4 hours, the culture medium was removed, washed with PBS for three times, fixed by 4% paraformaldehyde at room temperature for 15 min, and then stained with 4′,6-diamidino-2-phenylindole (DAPI) for CLSM imaging.

To further investigate the nanoparticle internalization into tumor cells at different times. PC-9 and PC9-GR cells were seeded in six-well plates (1 × 10^5^ cells per well) for 24 hours, and the cells were incubated with HA-BPY-GEF-NPs (10 μM) for 0, 2, 4, and 8 hours, respectively. After culturing, the cells were washed by PBS and trypsinized for FCM analysis. To observe the cellular uptake, the cells were incubated with HA-BPY-GEF-NPs for different times and then washed with PBS for three times, fixed by 4% paraformaldehyde at room temperature for 15 min, and stained with DAPI for CLSM imaging.

The intracellular ROS generation was evaluated via FCM and fluorescence microscopy. For FCM analysis, PC-9 and PC9-GR cells were seeded in six-well plates (1 × 10^6^ cells per well). After incubating for 24 hours, the cells were treated with HA-BPY-GEF-NPs (10 μM) for another 12 hours, the cell medium was removed, and the fresh medium containing DCFH-DA (10 μM) was added. After incubating for 30 min, the laser-treated group was irradiated with a 660-nm laser (100 mW/cm^2^) for 10 min, and then the cells were washed with PBS and harvested for FCM analysis. For fluorescence imaging, after incubation with DCFH-DA for 30 min, the laser-treated group was irradiated with a 660-nm laser (100 mW/cm^2^) for 10 min, and then the cell medium was removed and PBS was added. Subsequently, the intracellular green fluorescence signal in different groups was observed under fluorescence microscopy.

### Mitochondrial membrane potential detection

The mitochondrial membrane potential of PC9-GR cells was detected by JC-1 assay kit. PC9-GR cells were seeded in confocal dishes (1 × 10^5^ cells per dish). After incubating for 24 hours, the cells were treated with different formulations for another 24 hours. The laser-treated group was irradiated with a 660-nm laser (100 mW/cm^2^) for 10 min and then incubated with JC-1 probe at 37°C for 20 min. Subsequently, the intracellular fluorescence signal in different groups was observed under CLSM.

### Responsiveness and lysosome localization of the nanoprobes in cells

For FCM detection, PC9-GR cells were seeded in six-well plates (1 × 10^5^ cells per well) for 24 hours. Then, the cells were treated with HA-BPY-NAP-NPs (10 μM) nanoprobe for 4 hours, and the laser-treated group was irradiated with a 660-nm laser (100 mW/cm^2^) for 10 min. Next, the cells were further incubated for different time (1, 4, 12, and 24 hours) and then were harvested and collected for FCM detection. PB 450-A channel, λ_ex_ = 405 nm; FITC channel, λ_ex_ = 488 nm.

For confocal detection, PC9-GR cells were seeded in confocal dishes (1 × 10^5^ cells per dish). After incubation for 24 hours, the cells were treated with HA-BPY-NAP-NPs (10 μM) nanoprobe for 4 hours, and then the laser-treated group was irradiated with a 660-nm laser (100 mW/cm^2^) for 10 min. Next, the cells were further incubated for different times (1, 4, 12, and 24 hours), and the intracellular fluorescence signal in different groups was observed under CLSM. Green channel, λ_ex_ = 488 nm and blue channel, λ_ex_ = 405 nm.

For lysosome localization evaluation, PC9-GR cells were seeded in confocal dishes (1 × 10^5^ cells per dish). After being incubated for 24 hours, the cells were treated with HA-BPY-NAP-NPs (10 μM) nanoprobe for 4 hours, and then they were irradiated with a 660-nm laser (100 mW/cm^2^) for 10 min or not. Next, the cells were further incubated for another 4 hours and then incubated with Lysotracker probe at 37°C for 10 min. Subsequently, the intracellular fluorescence signal in different groups was observed under CLSM. Green channel, λ_ex_ = 488 nm, red channel, λ_ex_ = 561 nm, and blue channel, λ_ex_ = 405 nm.

### In vitro cytotoxicity and apoptosis assay

To evaluate cell toxicity, PC-9 and PC9-GR cells were seeded in 96-well plates (5 × 10^3^ cells per well) for 24 hours, and then the cells were incubated with different drug formulations for another 48 hours. For the laser-treated group, the cells were irradiated with a 660-nm laser (100 mW/cm^2^) for 10 min at 12 hours after administration. Cell viability of different groups was determined by a CCK-8 kits. Moreover, Calcein-AM/PI staining kit was used to observe live and dead cells, and then the fluorescence images were recorded by a fluorescence microscope.

To evaluate cell apoptosis, PC-9 and PC9-GR cells were seeded in 12-well plates (1 × 10^5^ cells per well) for 24 hours, and the cells were cultured with different drug formulations. For the laser-treated group, the cells were irradiated with a 660-nm laser (100 mW/cm^2^) for 10 min at 12 hours after administration. After a total culture time of 24 hours, the cells were harvested and collected for annexin V-FITC and PI staining according to the manufacturer’s instructions. The stained cells were resuspended in PBS for FCM detection.

### Immunofluorescent staining

PC9-GR cells were seeded in confocal dishes (1 × 10^5^ cells per dish) and cultured overnight. The cells were treated with HA-BPY-GEF-NPs (10 μM) for another 24 hours and during this period were irradiated by 660-nm laser (100 mW/cm^2^) for 10 min. Meanwhile, the untreated cells served as a control. Next, the cell medium was removed and washed with PBS for three times, the cells were fixed with 4% paraformaldehyde for 15 min, washed with PBS for three times, and permeabilized with 0.2% Triton X-100 for 20 min. Then, the cells were blocked with 1% bovine serum albumin (BSA) at room temperature for 30 min. Subsequently, the cells were stained with IGF1R antibody overnight at 4°C and then stained with FITC-conjugated secondary antibody at room temperature for 1 hour. The nucleus was stained by DAPI for 5 min. Last, the cell images were acquired by CLSM.

### Western blotting analysis

For in vitro evaluation, PC9-GR cells were seeded in six-well plates (3 × 10^5^ cells per well) and cultured overnight. The cells were treated with different formulations for another 24 hours, and during the period, the laser-treated group was irradiated by 660-nm laser (100 mW/cm^2^) for 10 min. In the meanwhile, the untreated cells served as a control. Next, cells were collected and lysed in radioimmunoprecipitation assay buffer (Beyotime, Shanghai, China) supplemented with protease inhibitor (Beyotime, Shanghai, China). The total protein concentration was determined by the bicinchoninic acid (BCA) assay kit (Beyotime, Shanghai, China). Protein extracts were separated by 8% SDS–polyacrylamide gel electrophoresis gels, transferred to polyvinylidene difluoride, and then blocked with 2% BSA. The transferred membrane was performed with indicated primary antibodies, followed by incubation with HRP-conjugated secondary antibody. Last, Western blotting images of proteins were acquired by a multifunctional molecular imaging system (UVP ChemStudio 815, Analytik Jena, Germany). β-Actin served as an internal reference, and quantitative analysis of relative protein expression was conducted by ImageJ. For in vivo detection, when the tumor size reached about 100 mm^3^, the nude mice were randomly divided into three groups: saline group, HA-BPY-GEF-NPs group, and HA-BPY-GEF-NPs + laser group. The laser-treated groups were intravenously injected with HA-BPY-GEF-NPs. After 24 hours of circulation, the tumors were irradiated by 660-nm laser for 10 min. The tumors were harvested for protein extraction after treatments. Subsequently, the concentration of total protein was determined by BCA kit and used for further Western blotting assay similarly performed as described above.

### Cell migration assay

PC9-GR cells were seeded in 12-well plates at a density of 80% and cultured overnight. The cells were divided into six groups with different treatments: Control, HA-GEF-NPs, GEF, HA-BPY-GEF-NPs, HA-BPY-NPs + laser, and HA-BPY-GEF-NPs + laser, followed by scratching the cells using a 10-μl pipette tip and washed with PBS for three times. Then, Calcein-AM kit was used to stain live cells, and the scratch of each group cells was recorded by a fluorescence microscope (designated as the 0 hours). Subsequently, the cell culture medium was replaced with serum-free medium containing different formulations incubated for another 24 hours, and the laser-treated groups were irradiated with a 660-nm laser (100 mW/cm^2^, 10 min). After that, the medium was removed, and the cells were washed with PBS for three times and stained with Calcein-AM kit. The scratch of each group cells was recorded using a fluorescence microscope, and quantitative analysis of cell migration was conducted by ImageJ.

### Animal tumor models

All animal experiments were approved by the Institutional Animal Care and Use Committee (IACUC) of Sun Yat-sen University, with protocol number of SYSU-IACUC-2022-000891. BALB/c nude female mice at 4 to 6 weeks old were purchased from the Sun Yat-sen University Laboratory Animal Center. PC-9 tumor model was established by subcutaneous inoculation of PC-9 cells (5 × 10^6^ cells per mouse) in the flank of nude mice. PC9-GR tumor model was established by subcutaneous inoculation of PC9-GR cells (1 × 10^7^ cells per mouse) suspended in 100 μl of Matrigel/PBS mixture (1:1 v/v). The length (L) and width (W) of each tumor were measured using a caliper, and the tumor volume was calculated according to the formula: V = (L × W^2^)/2.

### In vivo biodistribution analysis

When the tumor volume of nude mice reached around 100 mm^3^, PC-9 or PC9-GR tumor–bearing mice were randomly divided into two groups (*n* = 3). The mice were intravenously injected with free Ada-BPY and HA-BPY-GEF-NPs, respectively. The excitation wavelength for the photosensitizer dye is 630 nm, and the emission wavelength is 680 nm. At different time points after injection (0, 6, 12, 24, and 36 hours), fluorescence images were acquired by an in vivo imaging system (Night OWL II LB 983, Bertold, Germany). The mice were euthanized after 36 hours. The tumors and main organs, including heart, liver, spleen, lung, and kidneys, were harvested for ex vivo imaging.

### In vivo antitumor efficacy and safety evaluation

Female balb/c nude mice were subcutaneously inoculated with PC9 or PC9-GR cells 7 days before treatment. When the tumor volume reached about 100 mm^3^ (designated as the 0th day), PC-9 or PC9-GR tumor–bearing mice were randomly divided into six groups (*n* = 5). The mice were performed with the following different treatments: G1, saline; G2, free GEF; G3, HA-BPY-GEF-NPs; G4, HA-GEF-NPs; G5, HA-BPY-NPs + laser; and G6, HA-BPY-GEF-NPs + laser at an equivalent dose of GEF 3.2 mg/kg or Ada-BPY dose of 3.6 mg/kg for three times at a time interval of 3 day. The laser groups were treated with 660-nm NIR laser irradiation (200 mW/cm^2^) at 24 hours after injection for 10 min. The tumor size and body weight of all mice were measured every 2 days and monitored for 20 days. At day 21, all the mice were euthanized, and the tumors were collected and weighed as well as photographed. Furthermore, the tumors were stained by H&E and TUNEL to further evaluate the therapeutic effect.

To investigate in vivo toxicity, after completion of the treatment experiments, the blood samples from the represented mice were collected and blood biochemical parameters including AST, ALT, ALB, UREA, and CREA were determined. Moreover, the major organs (heart, liver, spleen, lung, and kidney) of represented mice were harvested for H&E staining for toxicity analysis after the different treatments.

### Statistical analysis

The experiments in this work were performed at least with triplicates, and all data are shown as means ± SD. Statistical comparisons between groups were evaluated by Student’s *t* test. (*P < 0.05, **P < 0.01, ***P < 0.001).

## Supplementary Materials

### This PDF file includes:

Supplementary Text Figs. S1 to S71 References
