# Supplementary material for: An “AND” logic gate–based supramolecular therapeutic nanoplatform for combatting drug-resistant non–small cell lung cancer
Source: Sci Adv. 2024 Sep 25;10(39):eadp9071. doi: 10.1126/sciadv.adp9071 (PMC11423878; doi:10.1126/sciadv.adp9071)
Supplement: Supplementary file 1 — Supplementary Text Figs. S1 to S71 References [file sciadv.adp9071_sm.pdf]

Science Advances
AAAS

Supplementary Materials for

An “AND” logic gate–based supramolecular therapeutic nanoplatform for combatting drug-resistant non–small cell lung cancer

Qili Huang *et al.*

Corresponding author: Xiaowei Zeng, zengxw23@mail.sysu.edu.cn; Yanli Zhao, zhaoyanli@ntu.edu.sg; Hongzhong Chen, chenhzh58@mail.sysu.edu.cn

Sci. Adv. **10**, eadp9071 (2024)
DOI: 10.1126/sciadv.adp9071

This PDF file includes:

Supplementary Text
Figs. S1 to S71
References

Supplementary Text

1. Materials

All commercially available reagents were used as purchased without further purification. 1-Adamantanecarbonyl chloride, 4-aminobenzylalcohol, 2-aminoethanethiol hydrochloride, 2-(2-azidoethoxy)ethanamine·HCl, N-butylamine, N,N-diisopropylethylamine (DIPEA), N,N'-dimethylethylenediamine, 4-dimethylaminopyridine (DMAP), di-tert-butyl dicarbonate (Boc_2O), 2-ethoxy-1-ethoxycarbonyl-1,2-dihydroquinoline (EEDQ), 1-ethyl-3-(3-dimethyl aminopropyl) carbodiimide (EDC), 4-nitrophenyl chloroformate, sodium hyaluronate, trifluoroacetic acid (TFA), and triphosgene were obtained from Aladdin (Shanghai, China). Gefitinib and 4-nitronaphthalene-1,8-dicarboxylic anhydride were purchased from Bide Pharmatech Ltd. (Shanghai, China). N-Hydroxysulfosuccinimide sodium salt (NHSS) and triethylamine (TEA) were obtained from Macklin (Shanghai, China). Ammonium acetate, butanol, boron (tri) fluoride etherate ($BF_3 \cdot Et_2O$), and nitromethane were purchased from Sigma Aldrich. Anhydrous solvents were purchased from Energy Chemical (Shanghai, China). Chloroform-d ($CDCl_3$), dimethyl sulfoxide-d_6 (DMSO-d_6), and deuterium oxide (D_2O) were obtained from Aladdin (Shanghai, China). The other solvents were purchased from General-reagent (Shanghai, China) and directly used without further purification. The ultrapure water (18.2 MΩ/cm) was acquired from a Millipore Milli-Q Ultrapure water system.

Papain (lyophilized powder, ≥10 units/mg protein) was obtained from Sigma Aldrich. Annexin V-FITC apoptosis detection kit, Calcein/PI cell viability/cytotoxicity assay kit, DAPI, and reactive oxygen species assay kit were purchased from Beyotime Biotechnology. JC-1 mitochondrial membrane potential assay kit was purchased from Solarbio. CCK-8 kit was purchased from ApexBio. The following monoclonal antibodies were purchased from Affinity (β-actin, AKT, EGFR, ERK, IKK, IκB, NF-κB, phospho-EGFR, phospho-ERK, phospho-AKT, phospho-IKK, phospho-NF-κB, and phospho-IκB). IGF1R and phospho-IGF1R were purchased from Cell Signaling Technology. Horseradish peroxidase (HRP)-conjugated secondary antibody and FITC-conjugated secondary antibody were purchased from Affinity. Gefitinib-sensitive PC-9 cells and Gefitinib-resistant PC9-GR cells were kindly provided by Prof. GuoHui Wan and XiaoLei Zhang from School of Pharmaceutical Sciences, Sun Yat-Sen University. NIH-3T3 and HUVEC cells were obtained from ATCC.

2. Characterization

^1H and ^{13}C NMR spectra were measured on a Bruker AvanceIII 400 MHz and 600 MHz spectrometer. High-resolution mass spectrometry (HRMS) was performed on a mass spectrometer (MAT95XP, ThermoFisher Scientific, USA). The drug release behavior was determined by high performance liquid chromatography (HPLC) system (LC-20 AT, Shimadzu, Japan) with an Agilent C18 reversed-phase column (250 mm × 4.6 mm, 5 μm). Size and zeta potential of nanoparticles were measured by dynamic light scattering (DLS) using a Malvern Zetasizer Nano Instruments (ZS90, Malvern, UK). Transmission electron microscope (HT7800, Hitachi, Japan)

was used to characterize the morphology and size of nanoparticles. UV-vis spectra and fluorescence spectra were recorded from spectrophotometer (T-U7S, Yoke, China) and fluorescence spectrometer (LS55, Perkin Elmer, USA) respectively. Cell imaging was performed by a laser confocal fluorescence microscope (CLSM) (LSM 880, Zeiss, Germany) and a fluorescence microscope (Eclipse Ti2, Nikon, Japan). The photodynamic performance of nanoparticles was characterized with a 660 nm laser (VCL-660nmM1-2W, Beijing Honglan Photoelectric Technology Co., Ltd., China). Cell apoptosis analysis was performed *via* flow cytometry (CytoFLEX, Beckman Coulter, USA). Fluorescence imaging of the tumor and main organs were performed by an in vivo imaging system (Night OWL II LB 983, Bertold, Germany). Images of PC9 and PC9-GR tumor-bearing mice for major organs and tumor pathological sections were obtained by fluorescence microscope (Eclipse Ti2, Nikon, Japan). Western blotting images of proteins were acquired by a multi-functional molecular imaging system (UVP ChemStudio 815, Analytik Jena, Germany).

3. Synthesis and characterization of compounds

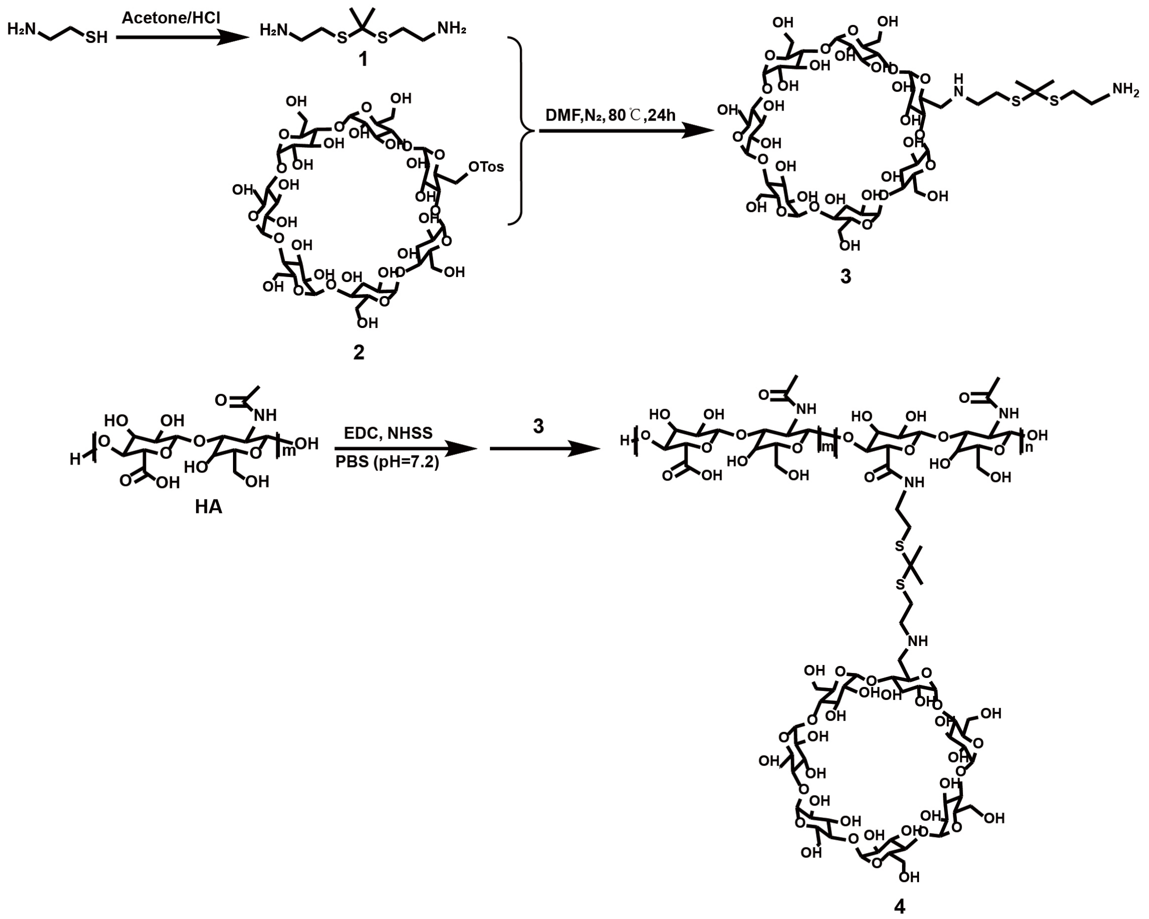

Fig. S1. Synthesis route of HA-TK-CD.

Compound 1

Compound **1** was synthesized according to the method reported previously (*67*). 2-Aminoethanethiol hydrochloride (1 eq, 11.36 g, 0.1 mol) and anhydrous acetone (2 eq, 11.62 g, 0.2 mol) were added into a 250 mL flask, followed by saturation with hydrogen chloride that was dried over concentrated sulfuric acid. After stirring for 1 h at room temperature, the flask was placed in a cooled ice-salt bath for product crystallization. The crystals were filtered, followed by washing for several times with cold ethanol. After drying in a vacuum oven overnight at room temperature, a snowy crystalline product was obtained. To obtain the dehydrochloride, acetone-[bis-(2-amino-ethyl)-dithioacetal]dihydrochloride was treated with cold NaOH aqueous solution (50 mL, 2 equivalents), followed by extraction with dichloromethane (4 × 50 mL) and drying with Na_2SO_4. After the solvent was removed, the final product as a transparent viscous liquid was obtained (6.9 g, 71%). ^{1}H NMR (400 MHz, Chloroform-d) δ 2.90 (t, 4H), 2.73 (t, 4H), 1.60 (s, 6H), 1.55 (s, 4H) (**Fig. S6**).

Compound 3

Compound **1** (20 eq, 3.01 g, 15.5 mmol) and 6-OTos-β-CD **2** (1 eq, 1 g, 0.776 mmol) were dissolved in DMF (25 mL). The solution was stirred and heated at 80 °C under N_2 atmosphere for 24 h. Then the crude product was precipitated in cold acetone and filtered, followed by washing with cold diethyl ether. After drying in vacuum overnight, the final light-yellow powder was obtained (717 mg, 70.5 %).^{1}H NMR (400 MHz, DMSO-d_6) δ 5.72 (s, 14H), 4.83 (s, 7H), 4.46 (s, 6H), 3.64 (s, 28H), 2.89 (d, J = 59.2Hz, 8H), 1.58 (s, 6H) (**Fig. S7**). HRMS (ES)$^+$: m/z calculated for $[C_{49}H_{88}N_2O_{34}S_2]^{2+}$: 656.2324 $[M+2H]^{2+}$; Found: 656.2334 (**Fig. S8**).

Compound 4 (HA-TK-CD)

EDC·HCl (100.1 mg, 0.522 mmol) and NHSS (113.4 mg, 0.522 mmol) were added to a solution of sodium hyaluronate (Mw=340000) (60 mg, 0.158 mmol) in phosphate buffer solution (PBS, 0.1 M, pH 7.2) (20 mL), and the mixture was stirred at 25 °C for 30 min. Then, the compound **3** (186.3 mg, 0.142 mmol) in PBS was added, and the mixture was stirred at room temperature for 24 h. The resulting solution was dialyzed against an excess amount of deionized water for 5 days. After being freeze-dried, the β-CD-modified HA **4** (HA-TK-CD) was obtained as white powder. The degree of substitution of the β-CD units was calculated to be about 17% by comparing the integrated peak area of the H1 proton of β-CD versus that of the N-acetyl protons of HA at 2.06 ppm. If all the carboxyl groups on the HA scaffold were fully substituted by β-CD, then theoretically, the peak area integral ratio of N-acetyl protons to H1 should be 3:7. However, the actual peak area integral ratio is 3:1.19. Consequently, the degree of substitution of CD can be calculated as 1.19/7 × 100% = 17%. ^{1}H NMR (400 MHz, D_2O) δ 5.13−5.17 (m, 1.19H, H of C-1 of β-CD), 4.56−4.62 (m, 2H, H of HA), 2.06 (s, 3H, H of methyl group of HA) (**Fig. S9**).

Compounds 5-9

Compounds **5-9** were synthesized based on the method reported by previous literature (*68*). Briefly, compound **5** (chalcone) was prepared by an aldol/dehydration reaction of the corresponding benzaldehyde and acetophenone derivatives. With sodium hydrosulfite as base, Michael addition of nitromethane to the chalcone to obtain the compound **6**. Next, condensation of **6** with ammonium acetate in n-Butanol under reflux for 24 h gave a moderate yield of compound **7**. Compound **7** was dissolved in anhydrous DCM, followed by adding TEA and BF_3•Et_2O to give compound **8** in high yields. Then compound **8** was dissolved in anhydrous DCM, NBS was added, and the mixture reaction was stirred at room temperature in the dark for overnight. Finally, the residue was purified by silica gel chromatography to afford **9** as a brown metallic solid in high yields.

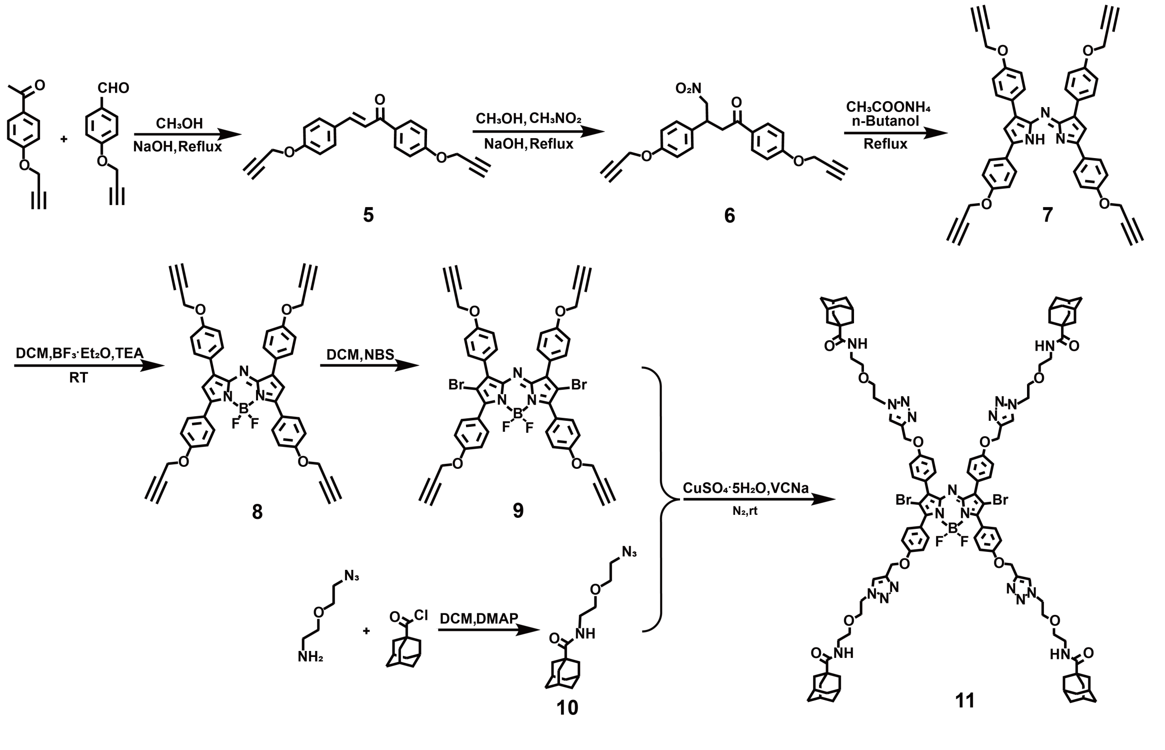

Fig. S2. Synthesis route of Ada-BPY.

Compound 10

2-(2-Azidoethoxy) ethanamin·HCl (1 eq, 130.15 mg, 1 mmol) and DMAP (1 eq, 122.17 mg, 1 mmol) were dissolved in anhydrous dichloromethane (20 mL) and cooled to 0 °C in an ice-water bath. A solution of 1-adamantanecarbonyl chloride (1.2 eq, 156.18 mg, 1.2 mmol) in anhydrous dichloromethane (10 mL) was added dropwise. The reaction mixture was allowed to warm to room temperature and the reaction was stirred for 24 h. The solvent was removed under vacuum. The residue was extracted with EtOAc and washed with brine, dried over anhydrous Na_2SO_4. The crude product was purified by column chromatography on silica gel to afford **10** as a white solid (210

mg, 71.9%). ^{1}H NMR (400 MHz, Chloroform-*d*) δ 6.05 (s, 1H), 3.66 (s, 2H), 3.56 (s, 2H), 3.47 (s, 2H), 3.38 (s, 2H), 2.03 (s, 3H), 1.86 (s, 6H), 1.71 (s, 6H) (**Fig. S10**).^{13}C NMR (100 MHz, Chloroform-*d*) δ 178.64, 70.00, 53.97, 42.02, 39.25, 39.02, 36.42, 31.11 (**Fig. S11**). HRMS (ES)$^+$: m/z calculated for $[C_{15}H_{24}N_4O_2Na]^+$: 315.1791 [M+Na]$^+$; Found: 315.1794 (**Fig. S12**).

Compound 11 (Ada-BPY)

Compound **9** (1 eq, 200 mg, 0.23 mmol) and compound **10** (1.2 eq, 80 mg, 0.27 mmol) were dissolved in THF/H_2O, catalytic amount of $CuSO_4{\cdot}5H_2O$ was added, then purged with N_2 for 15 min to remove oxygen. L-Ascorbic acid sodium salt (1.75 eq, 80 mg, 0.40 mmol) was added under N_2 atmosphere. The reaction mixture was stirred at room temperature for 24 h. After completion of the reaction, the solvent was removed under vacuum. The crude product was purified by column chromatography on silica gel using dichloromethane/methanol (v/v, from 20:1 to 40:3) as eluent to afford **11** as a blue metallic solid (398 mg, 85.0%). ^{1}H NMR (400 MHz, Chloroform-*d*) δ 7.81 (d, *J* = 53.6 Hz, 2H), 7.06 (s, 2H), 6.02 (s, 1H), 5.34 (s, 2H), 4.48 (s, 2H), 3.85 (s, 2H), 3.38 (s, 4H), 1.99 (s, 3H), 1.79 (s, 6H), 1.66 (s, 6H) (**Fig. S13**). ^{13}C NMR (100 MHz, Chloroform-*d*) δ 178.20, 160.26, 159.53, 143.67, 141.94, 132.43, 123.94, 122.91, 114.81, 70.68, 68.98, 62.90, 51.63, 40.60, 39.19, 38.14, 36.46, 28.10 (**Fig. S14**). HRMS (ES)$^+$: m/z calculated for $[C_{104}H_{124}N_{19}O_{12}BF_2Br_2]^{2+}$: 1018.9055 [M]$^{2+}$; Found: 1018.9089 (**Fig. S15**).

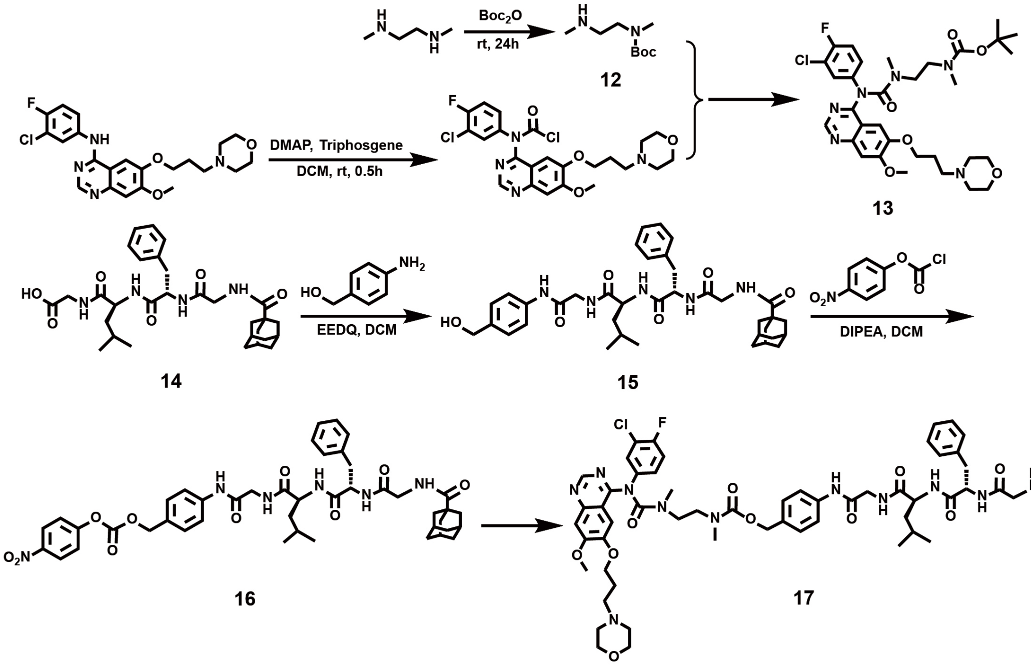

Fig. S3. Synthesis route of Ada-GFLG-GEF.

Compound 12

Compound **12** was synthesized according to the method reported in previous literature (*69*). N,N'-Dimethylethylenediamine (3.3 eq, 12.03 g, 136.2 mmol) was dissolved in anhydrous

dichloromethane (100 mL) under a N_2 atmosphere and cooled to 0 °C using ice-water bath. A solution of Boc_2O (1 eq, 9.0 g, 41.27 mmol) in anhydrous dichloromethane (30 mL) was added dropwise. The reaction mixture was allowed to warm to room temperature and the reaction was stirred overnight. Then, the solvent was removed under vacuum. The residue was extracted with EtOAc and washed with brine, dried over anhydrous Na_2SO_4 and evaporated under vacuum to afford **12** as light-yellow oil (4.85 g, 63%). ^{1}H NMR (400 MHz, Chloroform-d) δ 3.32 (s, 2H), 2.87 (s, 3H), 2.72 (s, 2H), 2.44 (s, 3H), 1.45 (s, 9H) (**Fig. S16**).

Compound 13

Gefitinib (1 eq, 1340.7 mg, 3 mmol) and DMAP (3.1 eq, 1136.2 mg, 9.3 mmol) were dissolved in anhydrous dichloromethane (20 mL). A solution of triphosgene (0.35 eq, 311.6 mg, 1.05 mmol) in anhydrous dichloromethane (10 mL) was added under N_2 atmosphere. The mixture was stirred at room temperature for 0.5 h. After that, the product **12** (2.3 eq, 1299.1 mg, 6.9 mmol) in anhydrous dichloromethane (10 mL) was added under N_2 atmosphere. The resultant mixture was stirred at room temperature for another 24 h. After completion of the reaction, the mixture was diluted with ethyl acetate, washed with brine, and the organic layer was dried over anhydrous Na_2SO_4. Then the solvent was removed under vacuum and the crude product was purified by column chromatography on silica gel eluted with dichloromethane/methanol (v/v, 100:1) to afford compound **13** as a white solid (390 mg, 19.6%). ^{1}H NMR (400 MHz, Chloroform-d) δ 8.88 (s, 1H), 7.31 (s, 1H), 7.14 (d, J = 7.2 Hz, 1H), 7.11 (s, 1H), 6.95 (s, 1H), 6.73 (s, 1H), 4.00 (s, 3H), 3.83 (s, 2H), 3.70 (s, 4H), 3.48 (s, 4H), 2.88 (s, 3H), 2.80 (s, 3H), 2.45 (s, 2H), 2.40 (s, 4H), 1.93 (s, 2H), 1.45 (s, 9H) (**Fig. S17**). ^{13}C NMR (150 MHz, Chloroform-d) δ 159.43, 158.63, 157.10, 155.74, 155.45, 153.58, 148.72, 140.46, 128.01, 125.73, 121.32, 108.28, 103.38, 66.35, 64.11, 56.35, 54.89, 52.81, 48.44, 45.98, 38.73, 35.94, 34.95, 28.45, 23.93 (**Fig. S18**). HRMS (ES)$^+$: m/z calculated for $[C_{32}H_{43}N_6O_6FCl]^+$: 661.2911 [M+H]$^+$; Found: 661.2866 (**Fig. S19**).

Compound 14

Compound **14** (Ada-GFLG-COOH) was prepared by solid phase peptide synthesis (SPPS). ^{1}H NMR (400 MHz, DMSO-d_6) δ 8.11 (s, 1H), 8.07 (s, 1H), 7.71 (s, 1H), 7.64 (s, 1H), 7.20 (s, 5H), 4.52 (s, 1H), 4.34 (s, 1H), 3.75 (s, 4H), 3.00 (s, 1H), 2.82 (s, 1H), 1.95 (s, 3H), 1.73 (s, 6H), 1.63 (s, 6H), 1.59-1.54 (m, 1H), 1.49 (s, 2H), 0.85 (s, 6H) (**Fig. S20**). ^{13}C NMR (100 MHz, DMSO-d_6) δ 172.66, 171.43, 170.85, 169.54, 129.80, 128.49, 126.68, 53.97, 51.27, 42.80, 41.39, 41.07, 39.00, 37.95, 23.50, 22.06 (**Fig. S21**). HRMS (ES)$^+$: m/z calculated for $[C_{30}H_{43}N_4O_6]^+$: 555.3177 [M+H]$^+$; Found: 555.3196 (**Fig. S22**).

Compound 15

Compound **14** (1 eq, 450 mg, 0.811 mmol), 4-aminobenzylalcohol (4 eq, 400.3 mg, 3.25 mmol) and EEDQ (3 eq, 600.9 mg, 2.43 mmol) were dissolved in anhydrous dichloromethane, and the mixture was stirred at room temperature for 24 h. After completion of the reaction, the mixture was extracted with DCM, washed with brine, and the organic layer was dried with anhydrous

Na_2SO_4.Then the solvent was removed under vacuum and the crude product was purified by column chromatography on silica gel eluted with dichloromethane/methanol (v/v, 20:1) to afford **15** as a white solid (475 mg, 89.0%). ^{1}H NMR (600 MHz, DMSO-d$_6$) δ 9.78 (s, 1H), 8.20 (s, 2H), 7.82 (s, 1H), 7.67 (s, 1H), 7.56 (s, 2H), 7.22 (d, J = 22.8 Hz, 7H), 5.11 (s, 1H), 4.52 (s, 1H), 4.42 (s, 2H), 4.30 (s, 1H), 3.86 (s, 2H), 3.66 (s, 1H), 3.52 (s, 1H), 3.01 (s, 1H), 2.80 (s, 1H), 1.93 (s, 3H), 1.72 (s, 6H), 1.63 (d, J = 20.4 Hz, 6H), 1.57 (s, 1H), 1.53 (s, 2H), 0.89 (s, 6H) (**Fig. S23**). ^{13}C NMR (150 MHz, DMSO-d$_6$) δ 177.95, 172.74, 171.40, 169.78, 167.78, 140.62, 131.13, 128.52, 127.43, 126.09, 120.15, 64.27, 53.30, 51.23, 42.83, 42.11, 41.02, 39.01, 37.81, 36.57, 33.32, 28.05, 24.55, 23.47, 22.10 (**Fig. S24**). HRMS (ES)$^+$: m/z calculated for $[C_{37}H_{50}N_5O_6]^+$: 660.3756 [M+H]$^+$; Found: 660.3748 (**Fig. S25**).

Compound 16

Compound **15** (1 eq, 300 mg, 0.455 mmol) was dissolved in anhydrous dichloromethane (10 mL) and cooled to 0 ºC under N_2 atmosphere. Afterwards, DIPEA (3.5 eq, 206.7 mg, 1.6 mmol) was introduced, and then a solution containing 4-nitrophenyl chloroformate (3 eq, 274 mg, 1.36 mmol) in anhydrous dichloromethane (10 mL) was added. The reaction was stirred at 0 ºC for 10 min and then stirred at room temperature for 24 h. After completion of the reaction, the solvent was removed and the crude was dissolved in dichloromethane and washed with 1 M aqueous $KHSO_4$ (2 ×). The organic layer was dried with Na_2SO_4, concentrated and purified by column chromatography on silica gel eluted with dichloromethane/methanol (v/v, 100:1) to afford compound **16** as a white solid (260 mg, 69.3%). ^{1}H NMR (400 MHz, DMSO-d$_6$) δ 9.92 (s, 1H), 8.33 (s, 1H), 8.18 (s, 2H), 7.82 (s, 1H), 7.66 (s, 3H), 7.58 (s, 1H), 7.44 (s, 2H), 7.21 (s, 5H), 5.26 (s, 1H), 4.54 (s, 1H), 4.32 (s, 1H), 3.89 (s, 2H), 3.71 (s, 1H), 3.54 (s, 1H), 3.05 (s, 1H), 2.83 (s, 1H), 1.94 (s, 3H), 1.73 (s, 6H), 1.66 (s, 6H), 1.60-1.58 (m, 1H), 1.55 (s, 2H), 0.87 (s, 6H) (**Fig. S26**). ^{13}C NMR (150 MHz, $CDCl_3$) δ 181.43, 174.80, 172.58, 170.96, 168.45, 157.56, 153.25, 146.61, 139.57, 136.36, 129.59, 129.20, 129.15, 127.70, 125.29, 121.82, 120.30, 70.82, 56.06, 53.01, 45.29, 43.96, 40.50, 37.89, 33.99, 28.81, 25.08, 23.30, 20.07 (**Fig. S27**). HRMS (ES)$^+$: m/z calculated for $[C_{44}H_{53}N_6O_{10}]^+$: 825.3818 [M+H]$^+$; Found: 825.3786; $[C_{44}H_{52}N_6O_{10}Na]^+$: 847.3637[M+Na]$^+$; Found: 847.3627 (**Fig. S28**).

Compound 17 (Ada-GFLG-GEF)

Compound **17** was synthesized according to the method reported in a previous literature (*70*). TFA (200 μL) was added to a solution of compound **13** (1.5 eq, 240.4 mg, 0.364 mmol) in anhydrous dichloromethane and the reaction was stirred at room temperature for 2 h. The solvent was removed under vacuum to afford the amine TFA salt, and the crude was used without further purification. The resulting TFA salt was dissolved in DMF and cooled to 0 ºC under N_2 atmosphere. DIPEA (12 eq, 564.5 mg, 4.37 mmol) and compound **16** (1 eq, 300 mg, 0.455 mmol) were added and the mixture was allowed to warm to room temperature and stirred overnight. The solvent was removed in vacuum and the residue was diluted with EtOAc and washed with brine, dried over anhydrous Na_2SO_4. The crude product was concentrated and purified by column chromatography

on silica gel using dichloromethane/methanol (v/v, 100:1 to 30:1) as eluent to afford **17** as a white solid (109 mg, 38.0%). ^{1}H NMR (400 MHz, DMSO-d$_6$) δ 9.84 (s, 1H), 8.80 (s, 1H), 8.16 (s, 2H), 7.81 (s, 1H), 7.65 (s, 1H), 7.56 (d, J = 16.3 Hz, 2H), 7.36 (s, 3H), 7.28 (s, 1H), 7.19 (s, 5H), 6.99 (s, 1H), 6.76 (s, 1H), 4.98 (d, J = 29.5 Hz, 2H), 4.49 (s, 1H), 4.29 (s, 1H), 3.96 (s, 3H), 3.86 (s, 3H), 3.71 (s, 2H), 3.52 (s, 4H), 3.41 (s, 3H), 3.13 (s, 1H), 3.02 (d, J = 13.4 Hz, 1H), 2.83 (s, 3H), 2.79 – 2.65 (m, 3H), 2.29 (s, 4H), 1.93 (s, 3H), 1.71 (s, 6H), 1.61 (s, 6H), 1.54 (s, 3H), 0.89 (s, 6H) (**Fig. S29**). ^{13}C NMR (100 MHz, DMSO-d$_6$) δ 177.04, 172.29, 171.82, 170.29, 167.44, 160.05, 158.89, 155.94, 153.27, 150.45, 149.12, 139.36, 137.86, 129.75, 128.82, 127.91, 126.71, 119.40, 113.36, 108.07, 101.69, 75.83, 72.88, 66.98, 57.15, 55.02, 54.11, 53.70, 51.45, 43.12, 37.81, 36.99, 28.03, 24.91, 23.47, 22.53, 19.23, 18.51, 16.31 (**Fig. S30**). HRMS (ES)$^+$: m/z calculated for $[C_{65}H_{83}N_{11}O_{11}FCl]^{2+}$: 623.7957 $[M+2H]^{2+}$; Found: 623.7968 (**Fig. S31**).

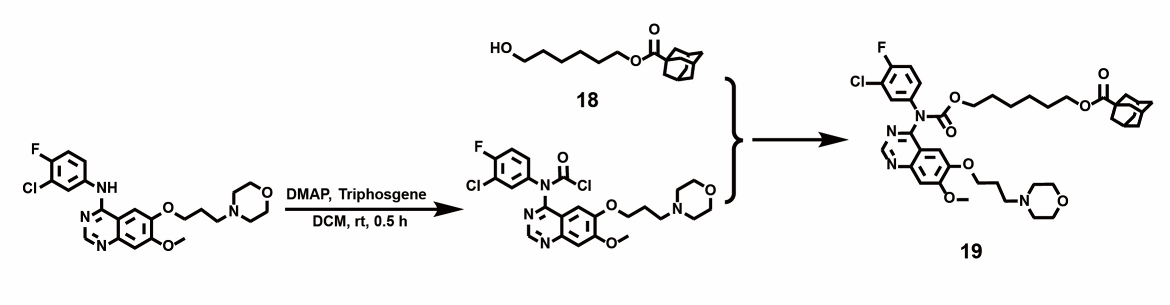

Fig. S4. Synthesis route of Ada-HE-GEF.

Compound 19 (Ada-HE-GEF)

Gefitinib (1 eq, 200 mg, 3 mmol) and DMAP (3.1 eq, 171 mg,1.4 mmol) were dissolved in anhydrous dichloromethane (10 mL). A solution of triphosgene (0.35 eq, 47.5 mg, 0.16 mmol) in anhydrous dichloromethane (5 mL) was added under N_2 atmosphere. The mixture was stirred at room temperature for 0.5 h. After that, the product **18** (1.5 eq, 189.3 mg, 0.675 mmol) in anhydrous dichloromethane (5 mL) was added under N_2 atmosphere. The resultant mixture was stirred at room temperature for another 24 h. After completion of the reaction, the mixture was diluted with ethyl acetate, washed with brine, and the organic layer was dried over anhydrous Na_2SO_4. Then, the solvent was removed under vacuum and the crude product was purified by column chromatography on silica gel eluted with dichloromethane/methanol (v/v, 80:1) to afford compound **19** (60 mg, 17.7%). ^{1}H NMR (500 MHz, Chloroform-d) δ 9.04 (s, 1H), 7.45 (dd, J = 6.4, 2.7 Hz, 1H), 7.37 (s, 1H), 7.21 (s, 1H), 7.11 (s, 1H), 7.06 (s, 1H), 4.17 (s, 2H), 4.11 (s, 2H), 4.05 (s, 3H), 3.96 (s, 2H), 3.71 (s, 4H), 2.56 (s, 2H), 2.48 (s, 4H), 2.07 (s, 2H), 2.00 (s, 3H), 1.87 (s, 6H), 1.72 (s, 6H), 1.67 (s, 4H), 1.51 (s, 4H). (**Fig. S32**). ^{13}C NMR (125 MHz, Chloroform-d) δ 156.87, 154.19, 153.61, 151.02, 139.29, 124.38, 116.72, 107.05, 67.47, 63.78, 55.27, 53.73, 36.52, 31.51, 29.67, 27.96, 25.39. (**Fig. S33**). HRMS: m/z calculated for $[C_{40}H_{51}N_4O_7FCl]^+$: 753.34248 $[M+H]^+$; Found: 753.34214 (**Fig. S34**).

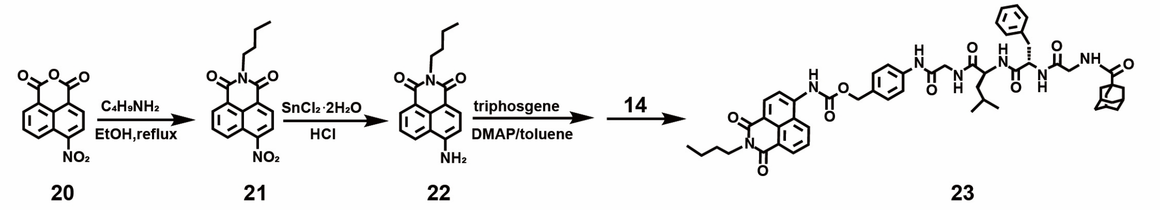

Fig. S5. Synthesis route of Ada-GFLG-NAP.

Compound 21

4-Nitronaphthalene-1,8-dicarboxylic anhydride **20** (1 eq, 300 mg, 1.30 mmol) was dissolved in ethanol (20 mL). N-Butylamine (2 eq, 258 μL, 2.60 mmol) was subsequently added to the reaction mixture and the solution was heated to reflux for 6 h. After cooling down to room temperature, the solvent was removed under vacuum, and the crude product was purified by column chromatography on silica gel eluted with dichloromethane/petroleum ether (v/v, 1:3 to 1:2) to afford **21** as a white solid (142.5 mg, 36.7%). ^{1}H NMR (400 MHz, DMSO-d$_6$) δ 8.71 (s, 1H), 8.59 (s, 2H), 8.55 (s, 1H), 8.09 (s, 1H), 4.04 (s, 2H), 1.63 (s, 2H), 1.37 (s, 2H), 0.93 (s, 3H) (**Fig. S35**). ^{13}C NMR (100 MHz, Chloroform-d) δ 163.32, 162.50, 132.42, 129.94, 129.78, 129.27, 127.06, 123.91, 123.69, 123.08, 40.67, 30.37, 20.34, 13.80 (**Fig. S36**). HRMS (ES)$^+$: m/z calculated for $[C_{16}H_{14}N_2O_4]^+$: 298.0948 $[M]^+$; Found: 298.0497 (**Fig. S37**).

Compound 22 (NAP-NH$_2$)

Compound **21** (1 eq, 120 mg, 0.4 mmol) was dissolved in ethanol (10 mL) and then the solution of $SnCl_2·2H_2O$ (6 eq, 543.8 mg, 2.41 mmol) in concentrated hydrochloric acid (1 mL) was added dropwise at room temperature. After refluxing for 8 h, the reaction was allowed to cool to room temperature and quenched with aqueous solution of Na_2CO_3 (10%). The precipitate was collected by filtration, and washed with water (3 × 10 mL). Then the crude product was purified by column chromatography on silica gel using dichloromethane/petroleum ether (v/v, 2:1) as eluent to afford **22** as a brown yellow solid (57.0 mg, 53.1%). ^{1}H NMR (400 MHz, DMSO-d$_6$) δ 8.61 (s, 1H), 8.43 (s, 1H), 8.20 (s, 1H), 7.65 (s, 1H), 7.42 (s, 2H), 6.85 (s, 1H), 4.00 (s, 2H), 1.58 (s, 2H), 1.33 (s, 2H), 0.91 (s, 3H) (**Fig. S38**). ^{13}C NMR (100 MHz, DMSO-d$_6$) δ 164.23, 163.37, 153.14, 134.40, 131.44, 130.14, 129.73, 124.44, 122.27, 119.83, 108.62, 108.04, 30.31, 20.31, 14.22 (**Fig. S39**). HRMS (ES)$^+$: m/z calculated for $[C_{16}H_{17}N_2O_6]^+$:269.1285 $[M+H]^+$; Found: 269.1288 **Fig. S40**).

Compound 23 (Ada-GFLG-NAP)

Compound **23** was synthesized according to literature report (*71*). **22** (1 eq, 30 mg, 0.11 mmol) and DMAP (5.5 eq, 74 mg, 0.6 mmol) were dissolved in toluene (10 mL). Then triphosgene (3.6 eq, 120 mg, 0.4 mmol) in toluene (10 mL) was added to the above solution. The reaction mixture was heated to reflux for 6 h. After cooling to room temperature, the solvent was removed under vacuum. The residue was redissolved in anhydrous dichloromethane (10 mL). Peptide **14** (0.6 eq,

40 mg, 0.06 mmol) and TEA (5.5 eq, 60 μL, 0.6 mmol) were added to the reaction mixture under ice bath and the mixture was stirred overnight under room temperature. After reaction, the crude product was concentrated and purified by column chromatography on silica gel using ethyl acetate/petroleum ether (v/v, 1:10 to 1:1) as eluent to afford **23** as a light-yellow solid (36.0 mg, 63.8%). ^{1}H NMR (400 MHz, DMSO-d_6) δ 9.78 (s, 1H), 8.20 (s, 2H), 7.82 (s, 1H), 7.67 (s, 1H), 7.56 (s, 2H), 7.22 (d, J = 22.8 Hz, 7H), 5.11 (s, 1H), 4.52 (s, 1H), 4.42 (s, 2H), 4.30 (s, 1H), 3.86 (s, 2H), 3.66 (s, 1H), 3.52 (s, 1H), 3.01 (s, 1H), 2.80 (s, 1H), 1.93 (s, 3H), 1.72 (s, 6H), 1.63 (d, J = 20.4 Hz, 6H), 1.57 (s, 1H), 1.53 (s, 2H), 0.89 (s, 6H) (**Fig. S41**). ^{13}C NMR (100 MHz, DMSO-d_6) δ 177.94, 171.41, 169.79, 168.05, 163.40, 139.33, 137.87, 131.38, 129.76, 128.52, 126.84, 124.31, 119.44, 118.63, 117.53, 66.88, 36.55, 31.62, 30.29, 30.15, 29.48, 28.04, 24.55, 23.47, 22.10, 20.27, 14.20 (**Fig. S42**). HRMS (ES)$^+$: m/z calculated for $[C_{54}H_{63}N_7O_9Na]^+$:976.4579 $[M+Na]^+$; Found: 976.4546 (**Fig. S43**).

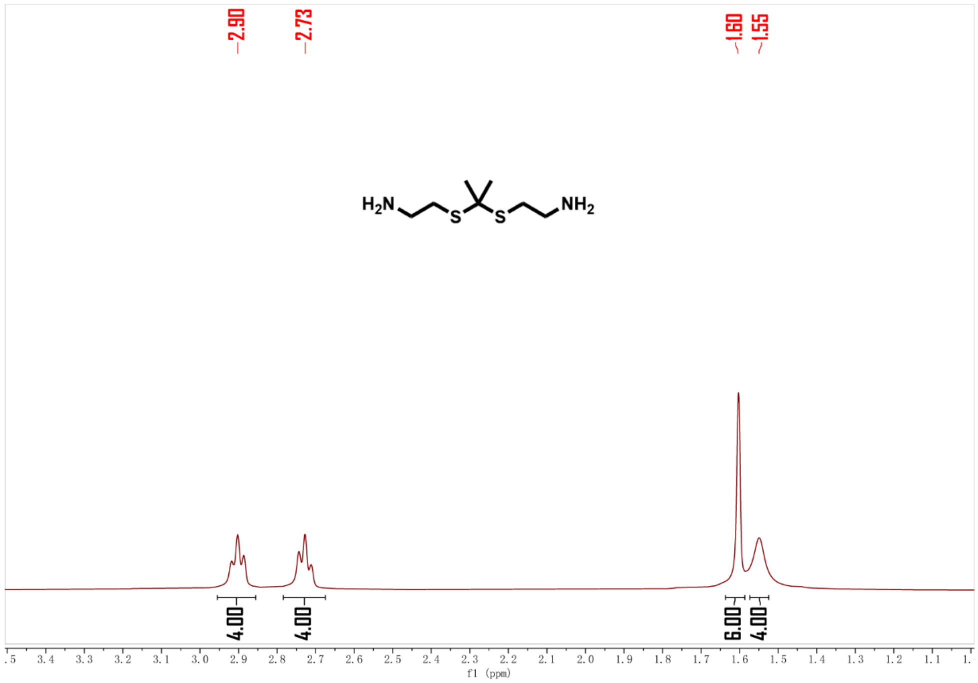

Fig. S6. ^{1}H NMR spectrum of compound 1 in $CDCl_3$.

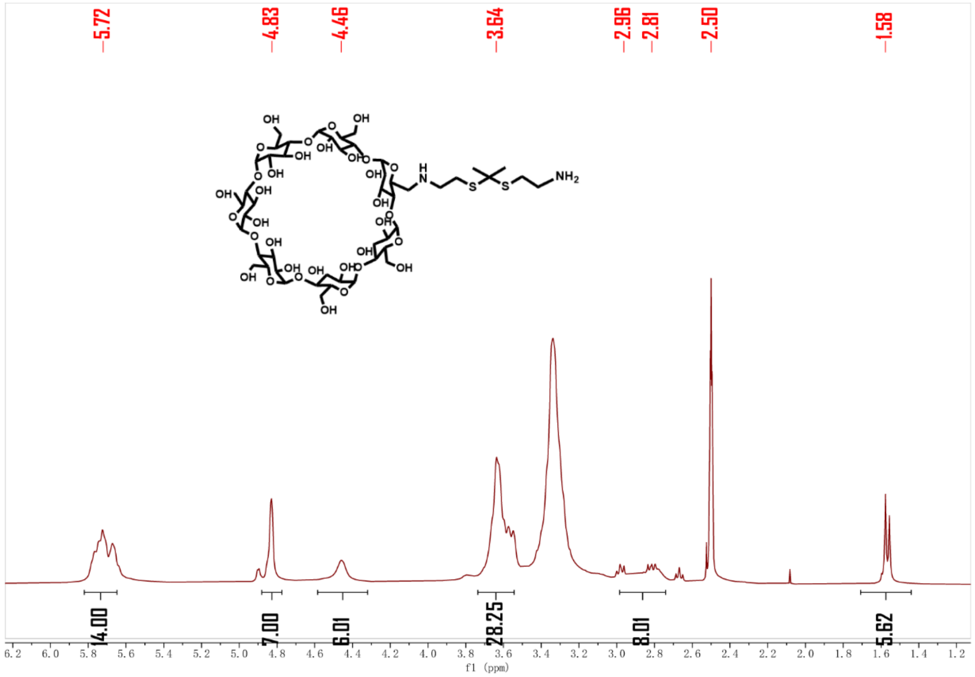

Fig. S7. ^{1}H NMR spectrum of compound 3 in DMSO-d_6.

Elmt	Val.	Min	Max	Elmt	Val.	Min	Max	Use Adduct
H	1	86	86	F	1	0	0	H
C	4	49	49	S	2	2	2	Na
N	3	2	2	Cl	1	0	0	K
O	2	34	34	Br	1	0	0	NH4

Error Margin (ppm):	20	DBE Range:	not fixed	Electron Ions:	both
HC Ratio:	unlimited	Apply N Rule:	yes	Use MSn Info:	no
Max Isotopes:	all	Isotope RI (%):	1.00	Isotope Res:	10000
MSn Iso RI (%):	75.00	MSn Logic Mode:	AND	Max Results:	1000

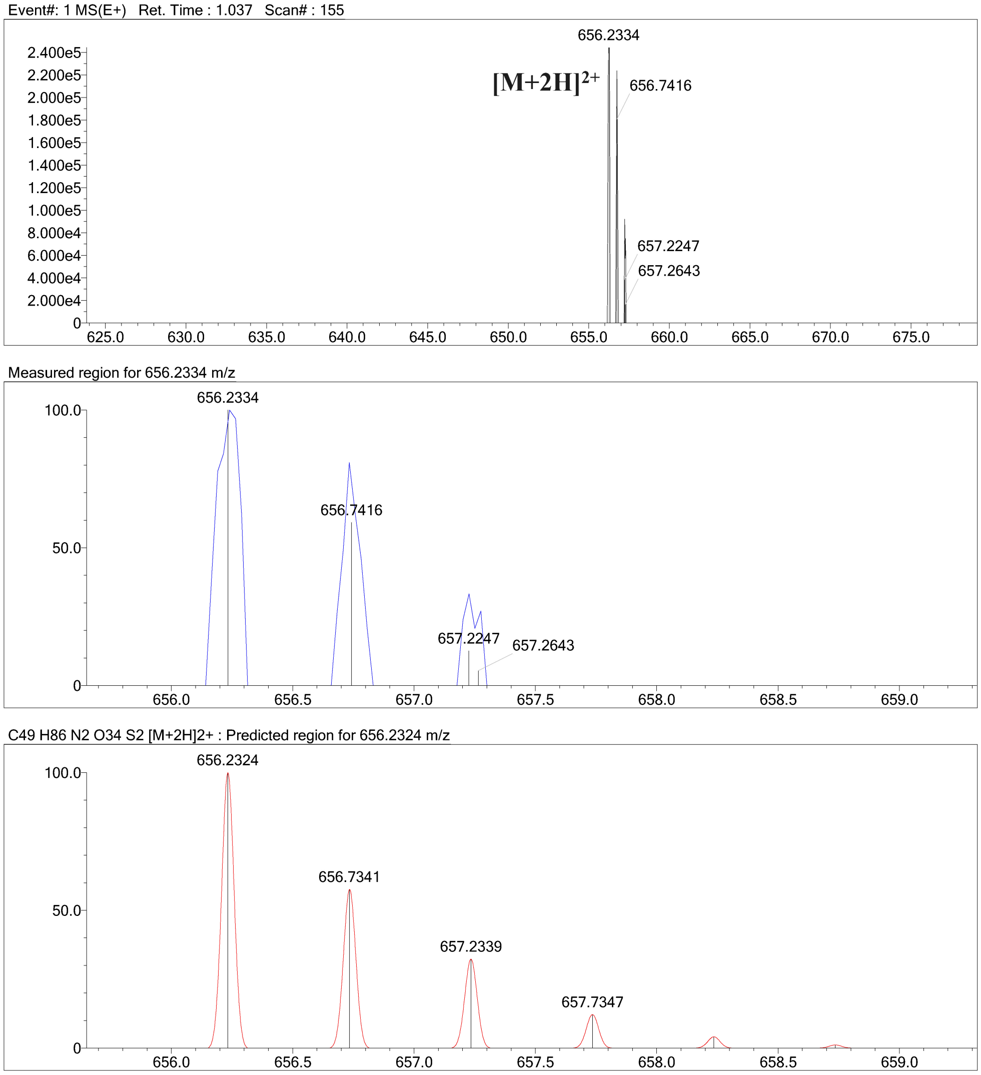

Rank	Score	Formula (M)	Ion	Meas. m/z	Pred. m/z	Df. (mDa)	Df. (ppm)	Iso	DBE
1	30.04	C49 H86 N2 O34 S2	[M+2H]2+	656.2334	656.2324	1.0	1.52	30.44	8.0

Fig. S8. HRMS spectrum of compound 3.

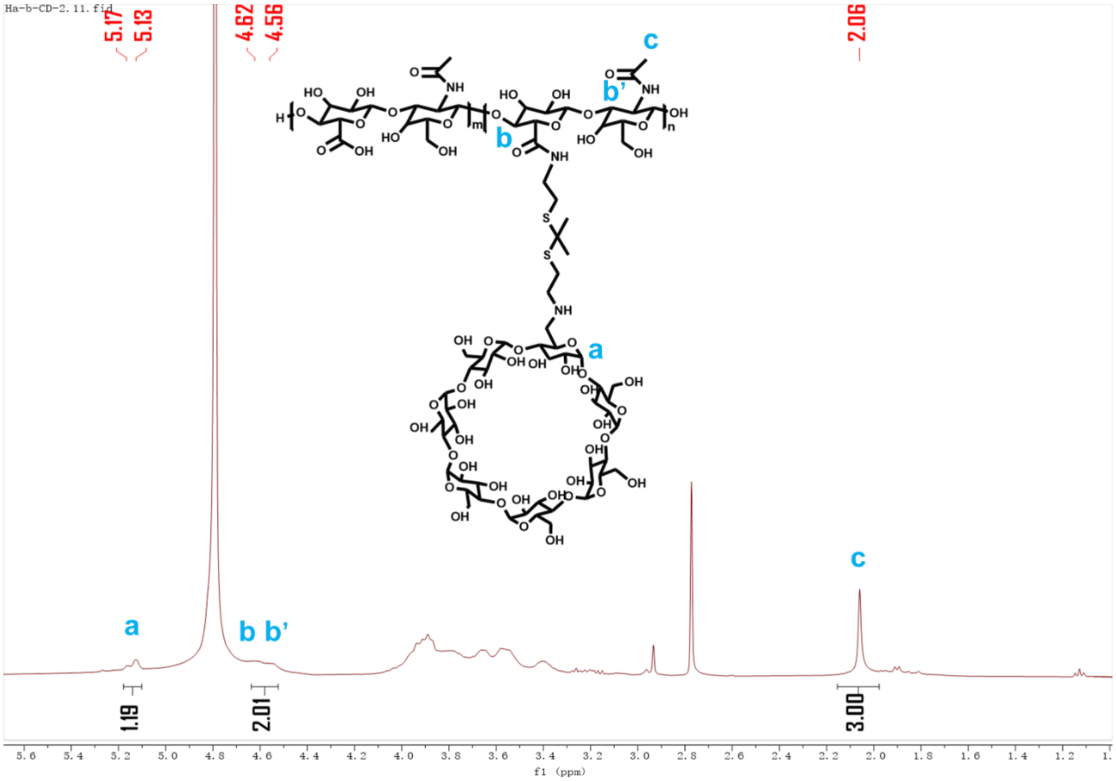

Fig. S9. ^{1}H NMR spectrum of compound HA-TK-CD in D_2O.

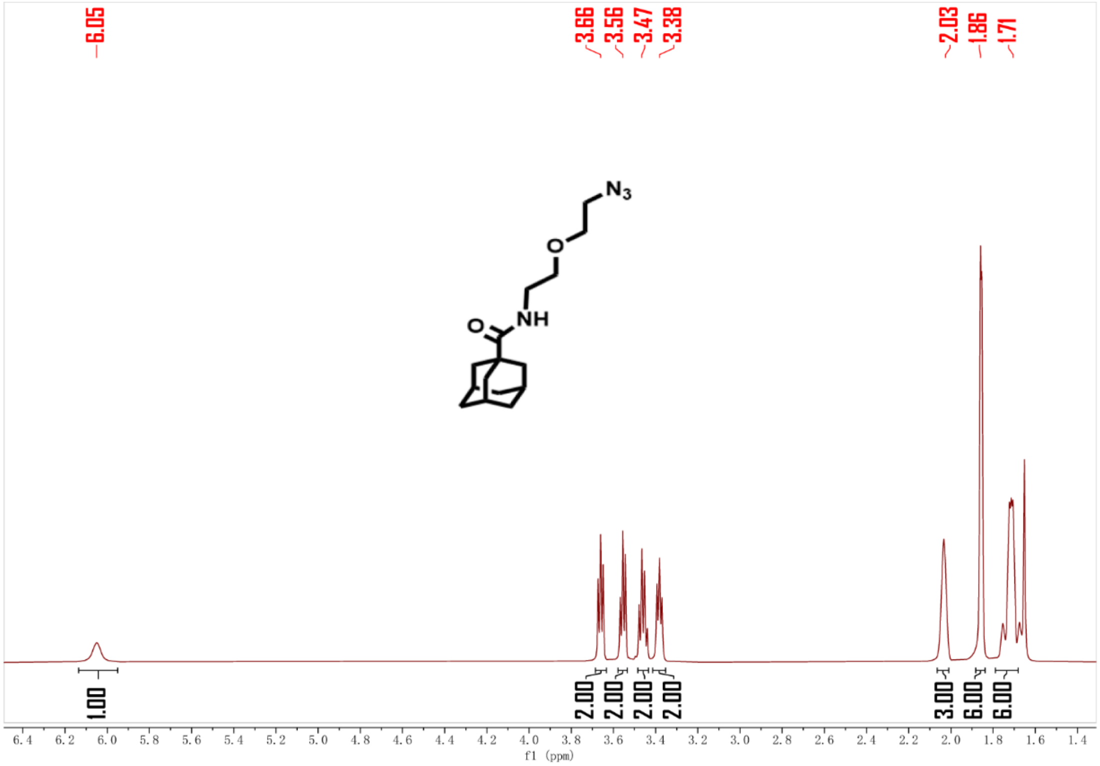

Fig. S10. ^{1}H NMR spectrum of compound 10 in $CDCl_3$.

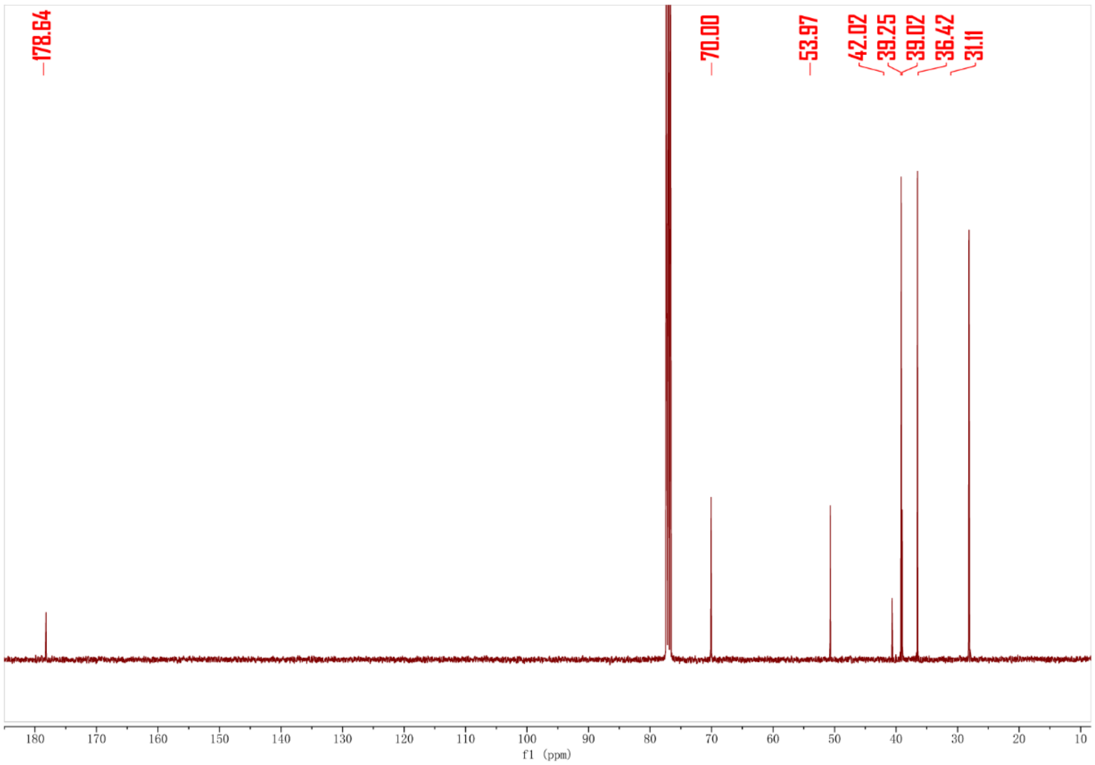

Fig. S11. ^{13}C NMR spectrum of compound 10 in $CDCl_3$.

Elmt	Val.	Min	Max	Elmt	Val.	Min	Max	Use Adduct
H	1	24	24	F	1	0	0	H
C	4	15	15					Na
N	3	4	4					K
O	2	2	2					NH4

Error Margin (mDa):	10.0	DBE Range:	not fixed	Electron Ions:	both
HC Ratio:	0.0 - 100.0	Apply N Rule:	yes	Use MSn Info:	no
Max Isotopes:	all	Isotope RI (%):	1.00	Isotope Res:	10000
MSn Iso RI (%):	75.00	MSn Logic Mode:	AND	Max Results:	1000

Event#: 1 MS(E+) Ret. Time : 3.530 Scan# : 529

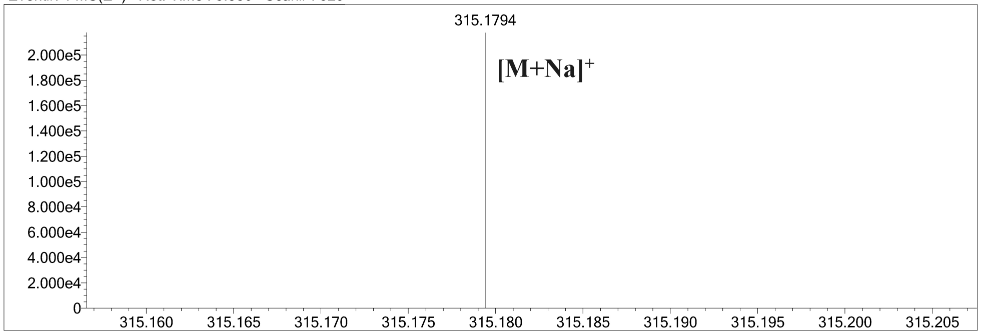

Measured region for 315.1794 m/z

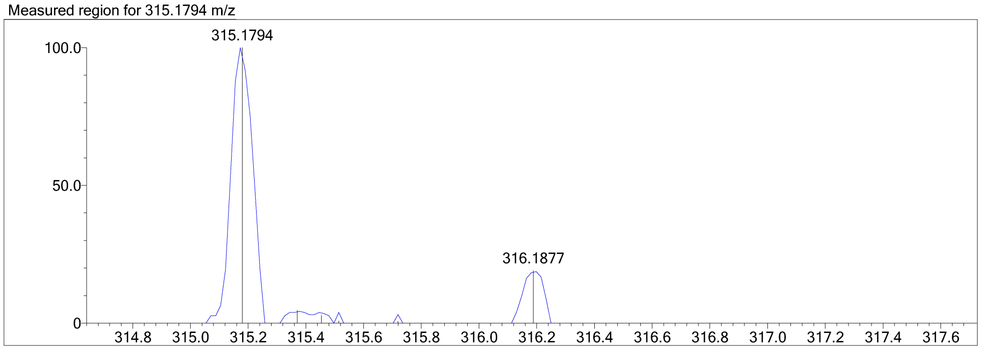

C15 H24 N4 O2 [M+Na]+ : Predicted region for 315.1791 m/z

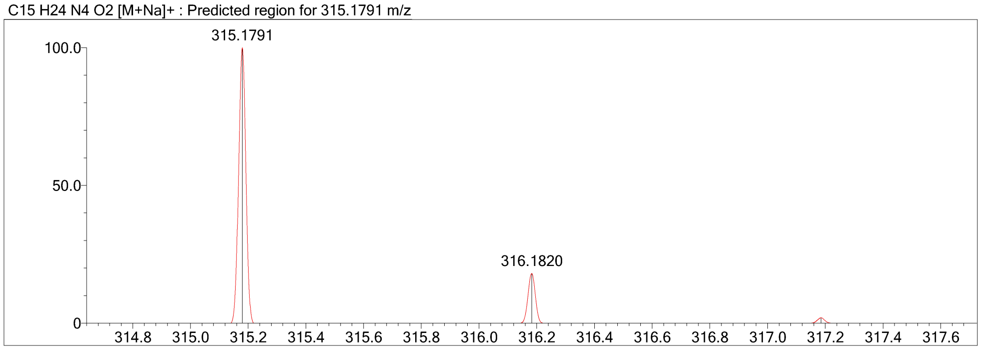

Rank	Score	Formula (M)	Ion	Meas. m/z	Pred. m/z	Df. (mDa)	Df. (ppm)	Iso	DBE
1	71.00	C15 H24 N4 O2	[M+Na]+	315.1794	315.1791	0.3	0.95	71.00	6.0

Fig. S12. HRMS spectrum of compound 10.

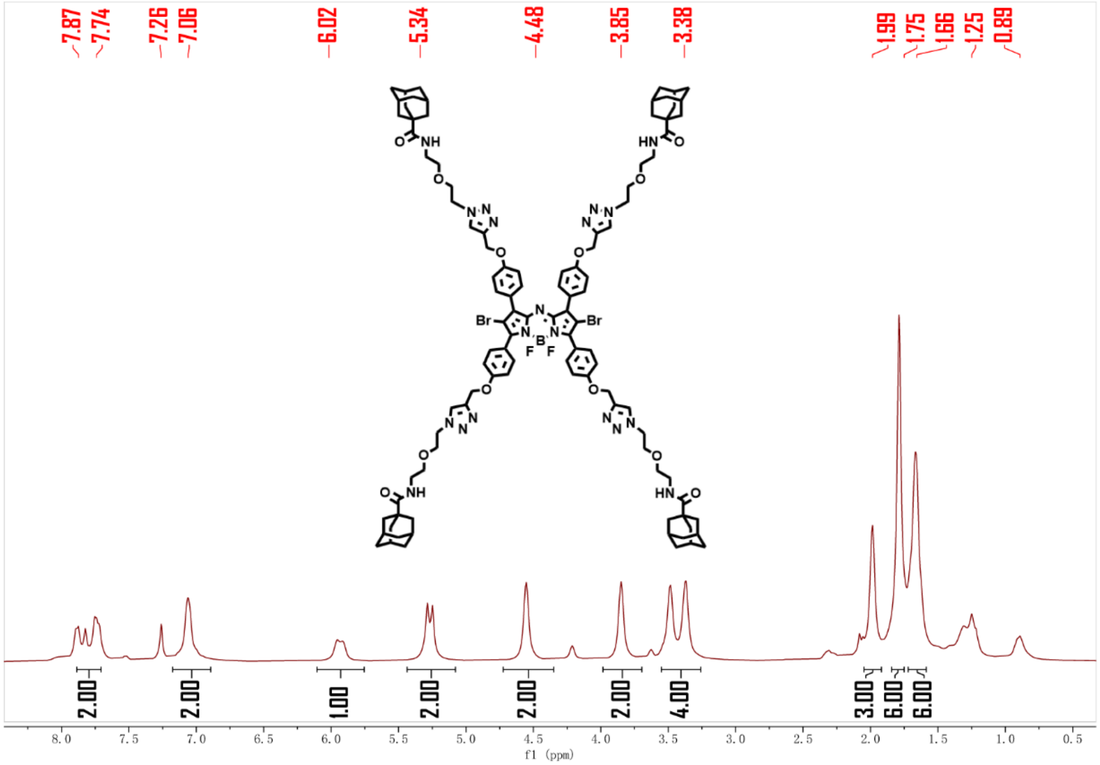

Fig. S13. ^{1}H NMR spectrum of Ada-BPY in $CDCl_3$.

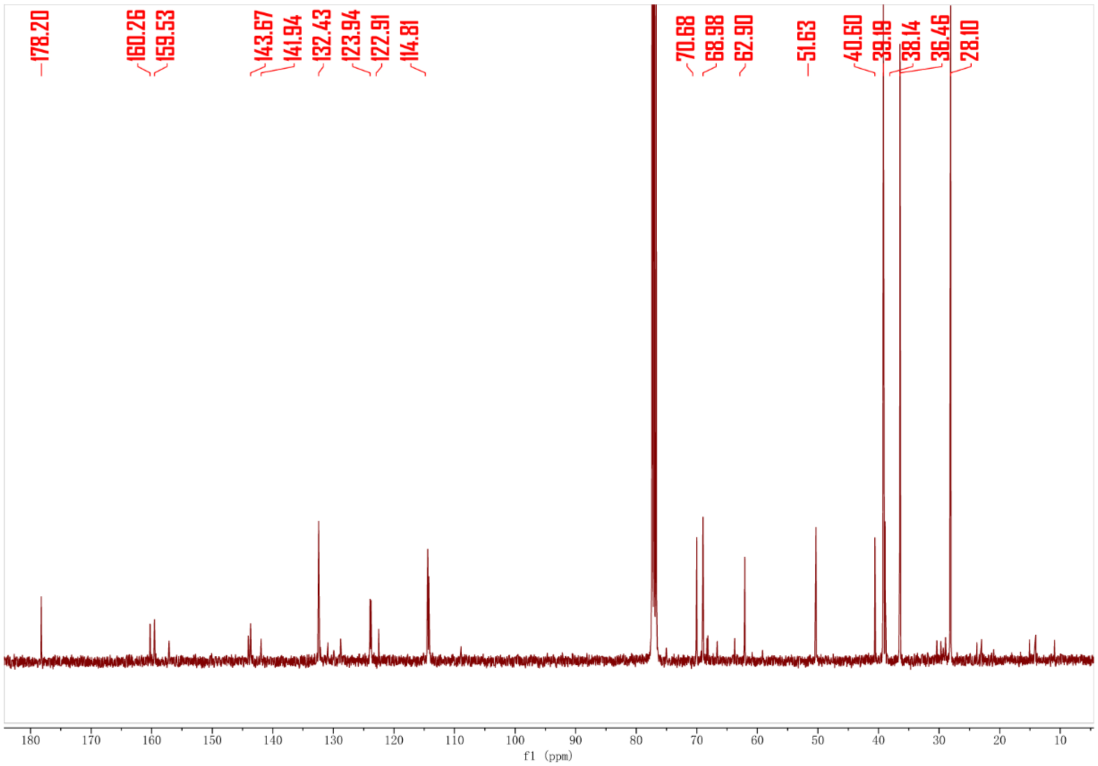

Fig. S14. ^{13}C NMR spectrum of Ada-BPY in $CDCl_3$.

Elmt	Val.	Min	Max	Elmt	Val.	Min	Max	Elmt	Val.	Min	Max	Use Adduct
H	1	120	124	O	2	10	12	Br	1	2	2	H
B	3	1	1	F	1	0	2					Na
C	4	104	104	S	2	0	0					K
N	3	19	19	Cl	1	0	0					NH4

Error Margin (mDa):	100.0	DBE Range:	not fixed	Electron Ions:	both
HC Ratio:	0.0 - 100.0	Apply N Rule:	yes	Use MSn Info:	no
Max Isotopes:	all	Isotope RI (%):	1.00	Isotope Res:	10000
MSn Iso RI (%):	75.00	MSn Logic Mode:	AND	Max Results:	1000

Event#: 1 MS(E+) Ret. Time : 1.610 Scan# : 241

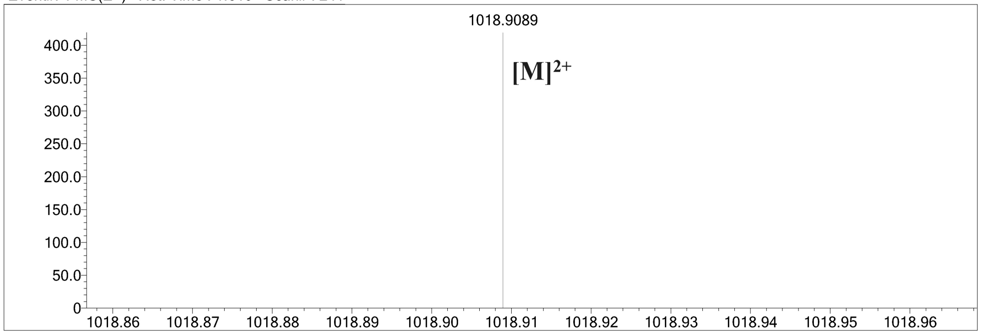

Measured region for 1018.9089 m/z

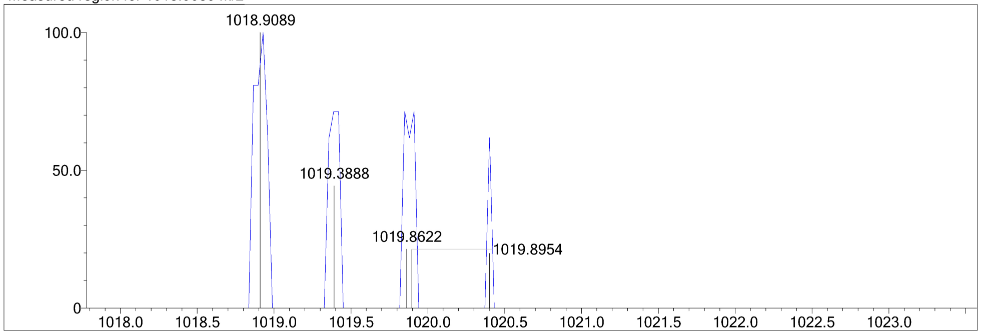

C104 H124 N19 O12 B F2 Br2 [M]2+ : Predicted region for 1018.9055 m/z

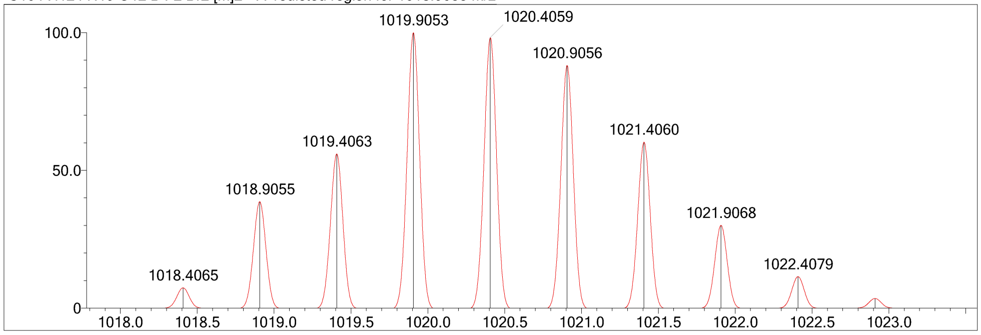

Rank	Score	Formula (M)	Ion	Meas. m/z	Pred. m/z	Df. (mDa)	Df. (ppm)	Iso	DBE
2	0.00	C104 H124 N19 O12 B F2 Br2	[M]2+	1018.9089	1018.9055	3.4	3.34	0.00	51.0

Fig. S15. HRMS spectrum of Ada-BPY.

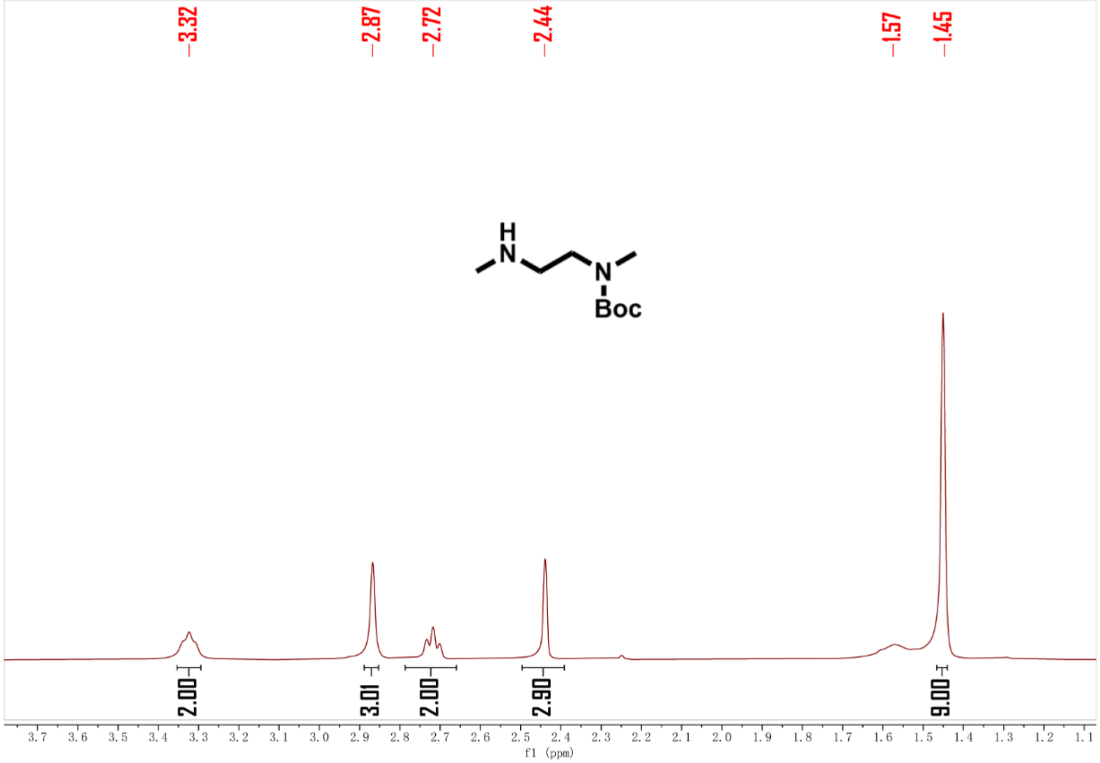

Fig. S16. ^{1}H NMR spectrum of compound 12 in $CDCl_3$.

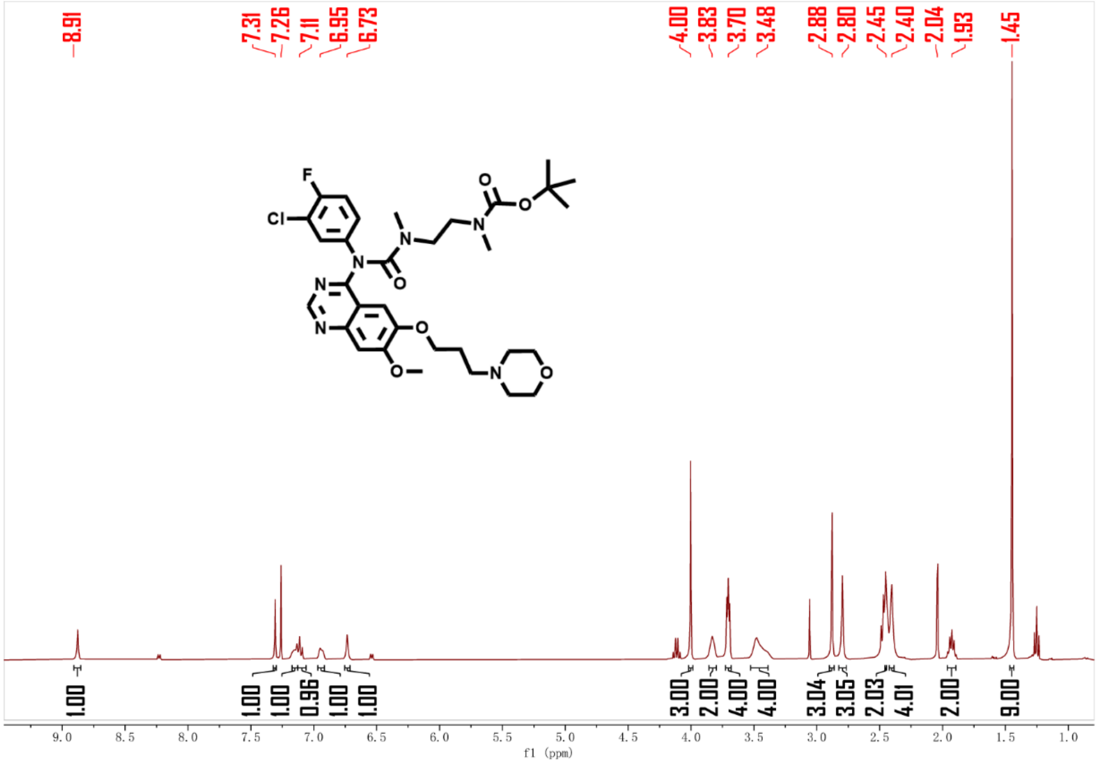

Fig. S17. ^{1}H NMR spectrum of compound 13 in $CDCl_3$.

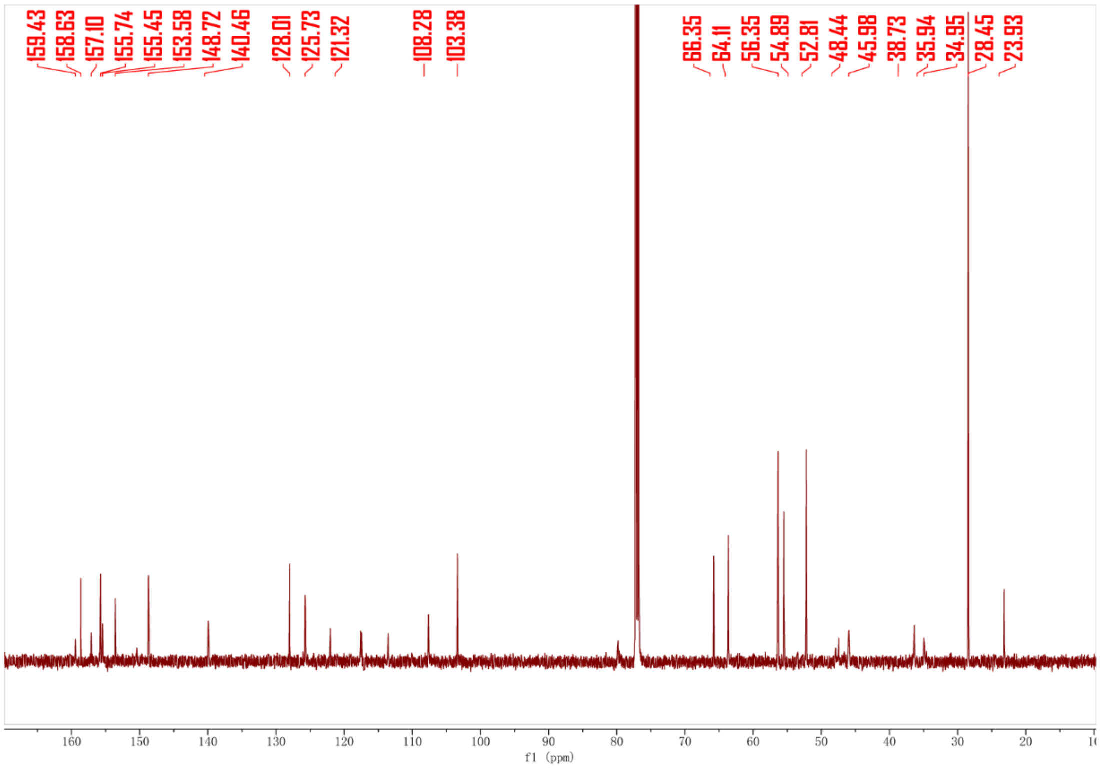

Fig. S18. ^{13}C NMR spectrum of compound 13 in $CDCl_3$.

Elmt	Val.	Min	Max
H	1	42	42
C	4	32	32
N	3	6	6
O	2	6	6

Elmt	Val.	Min	Max
F	1	1	1
Cl	1	1	1

Use Adduct
H
Na
K
NH4

Error Margin (ppm):	20	DBE Range:	-100.0 - 200.0	Electron Ions:	odd
HC Ratio:	0.0 - 100.0	Apply N Rule:	yes	Use MSn Info:	no
Max Isotopes:	all	Isotope RI (%):	1.00	Isotope Res:	10000
MSn Iso RI (%):	75.00	MSn Logic Mode:	OR	Max Results:	1000

Event#: 1 MS(E+) Ret. Time : 0.690 Scan# : 103

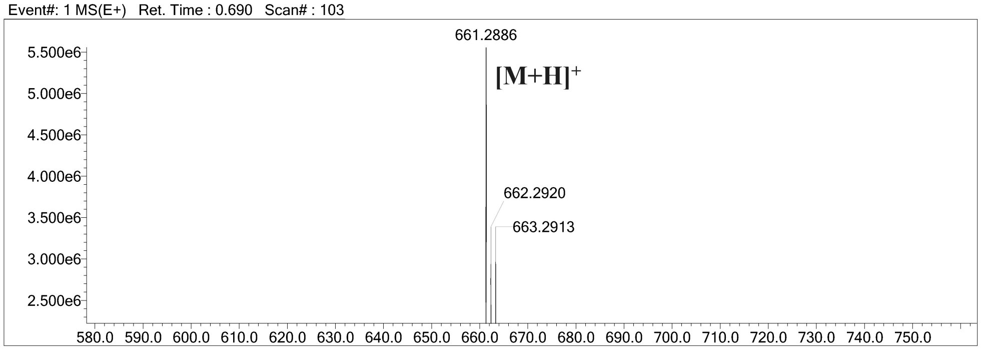

Measured region for 661.2886 m/z

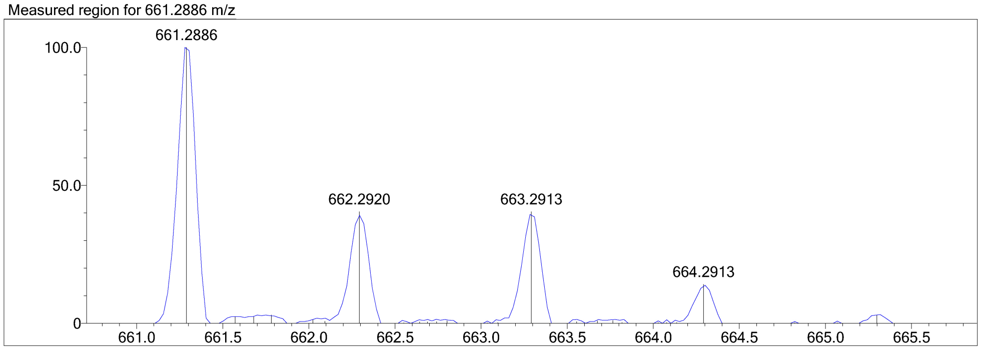

C32 H42 N6 O6 F Cl [M+H]+ : Predicted region for 661.2911 m/z

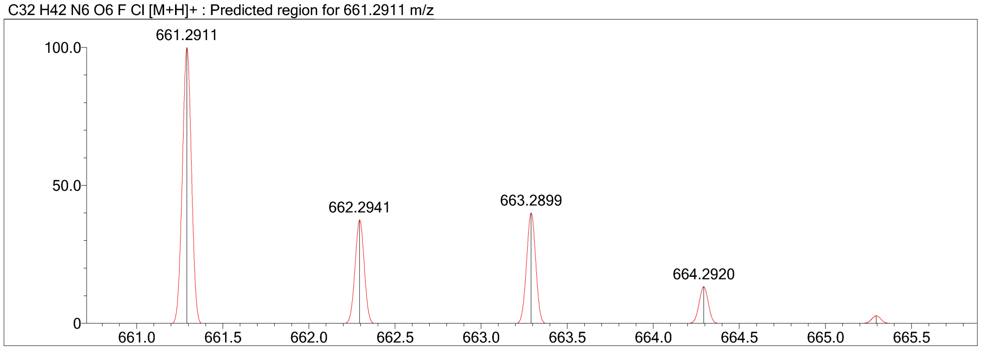

Rank	Score	Formula (M)	Ion	Meas. m/z	Pred. m/z	Df. (mDa)	Df. (ppm)	Iso	DBE
1	91.46	C32 H42 N6 O6 F Cl	[M+H]+	661.2886	661.2911	-2.5	-3.78	98.29	14.0

Fig. S19. HRMS spectrum of compound 13.

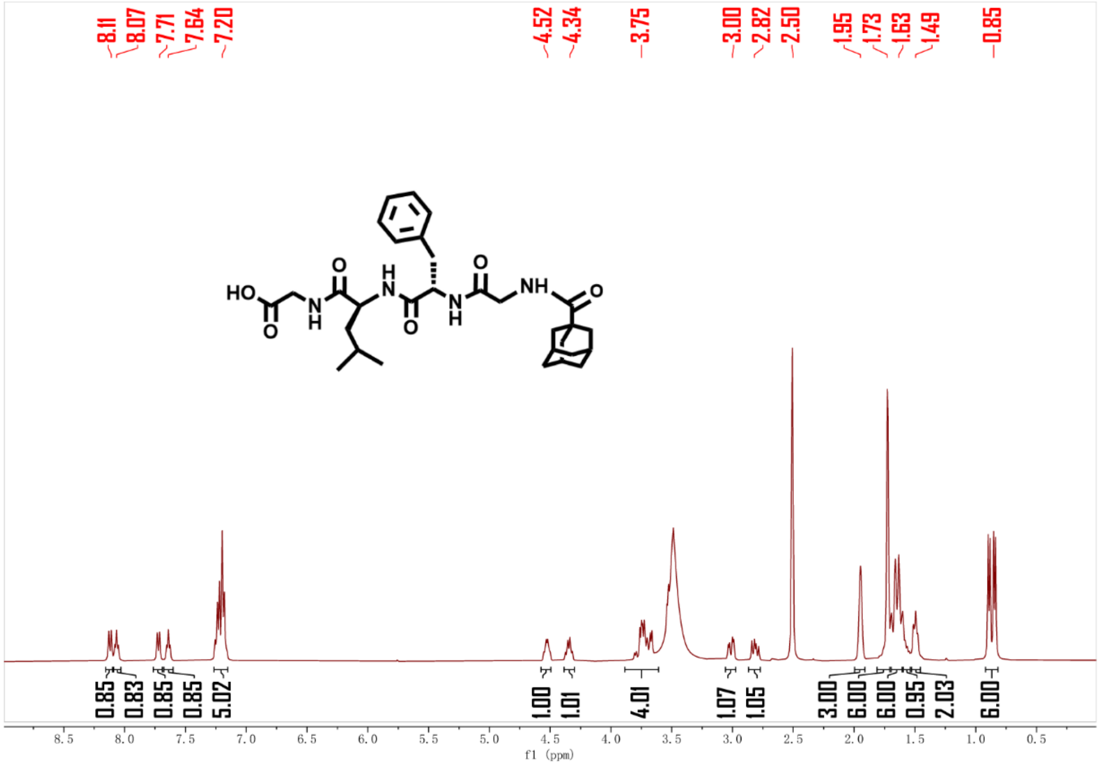

Fig. S20. ^{1}H NMR spectrum of compound 14 in DMSO-d_6.

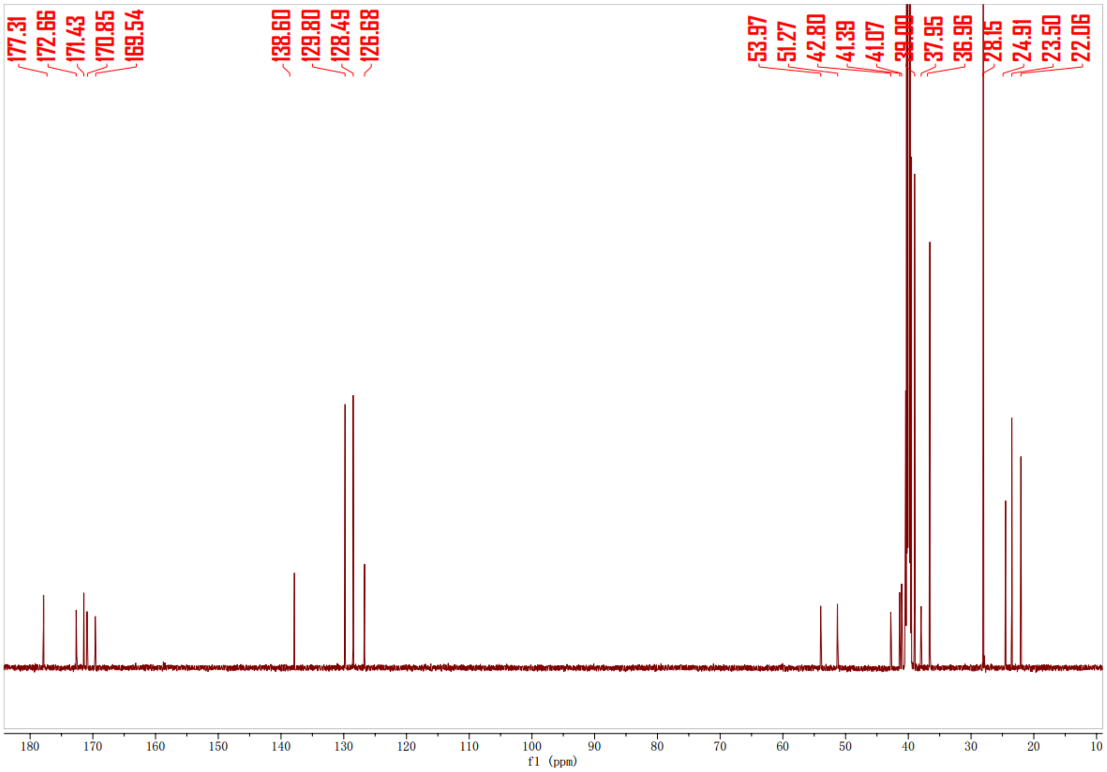

Fig. S21. ^{13}C NMR spectrum of compound 14 in DMSO-d_6.

Elmt	Val.	Min	Max
H	1	42	42
C	4	30	30
N	3	4	4
O	2	6	6

Use Adduct
H
Na
K
NH4

Error Margin (ppm):	20	DBE Range:	-100.0 - 200.0	Electron Ions:	odd
HC Ratio:	0.0 - 100.0	Apply N Rule:	yes	Use MSn Info:	no
Max Isotopes:	all	Isotope RI (%):	1.00	Isotope Res:	10000
MSn Iso RI (%):	75.00	MSn Logic Mode:	OR	Max Results:	1000

Event#: 1 MS(E+) Ret. Time : 0.863 Scan# : 129

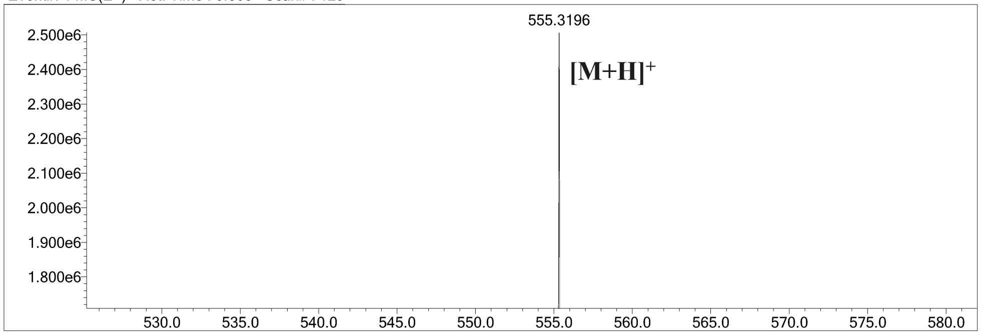

Measured region for 555.3196 m/z

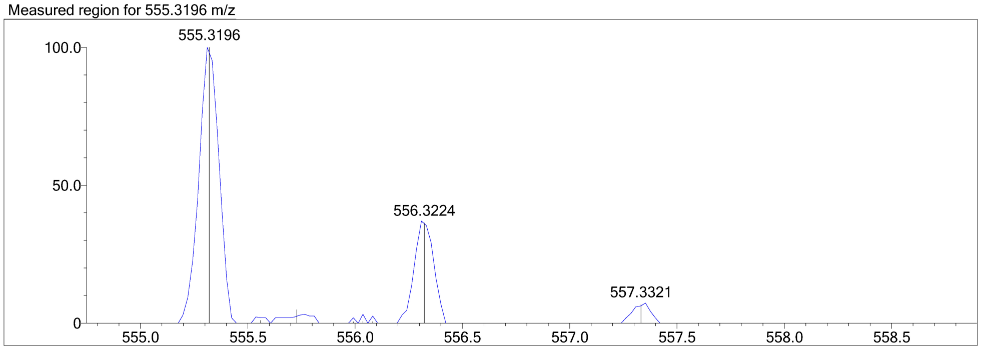

C30 H42 N4 O6 [M+H]+ : Predicted region for 555.3177 m/z

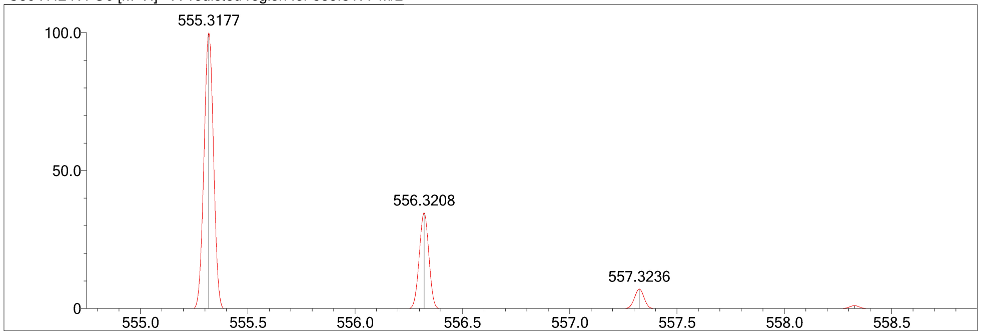

Rank	Score	Formula (M)	Ion	Meas. m/z	Pred. m/z	Df. (mDa)	Df. (ppm)	Iso	DBE
1	78.49	C30 H42 N4 O6	[M+H]+	555.3196	555.3177	1.9	3.42	83.55	12.0

Fig. S22. HRMS spectrum of compound 14.

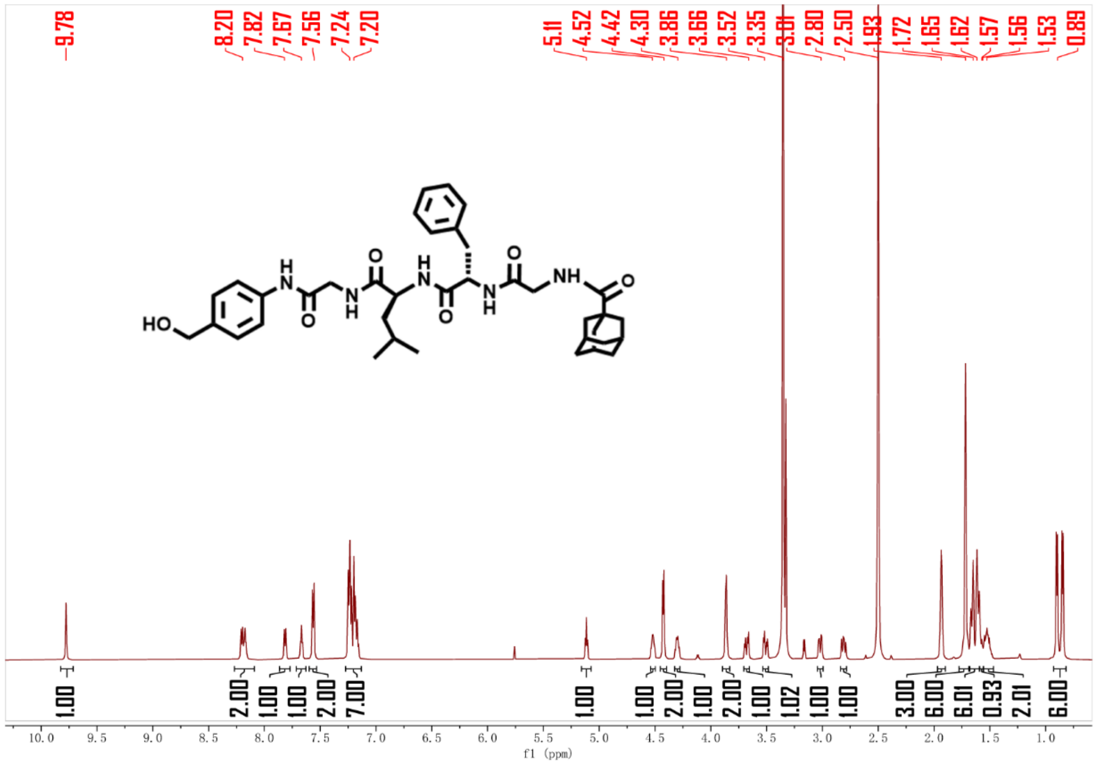

Fig. S23. ^{1}H NMR spectrum of compound 15 in DMSO-d$_6$.

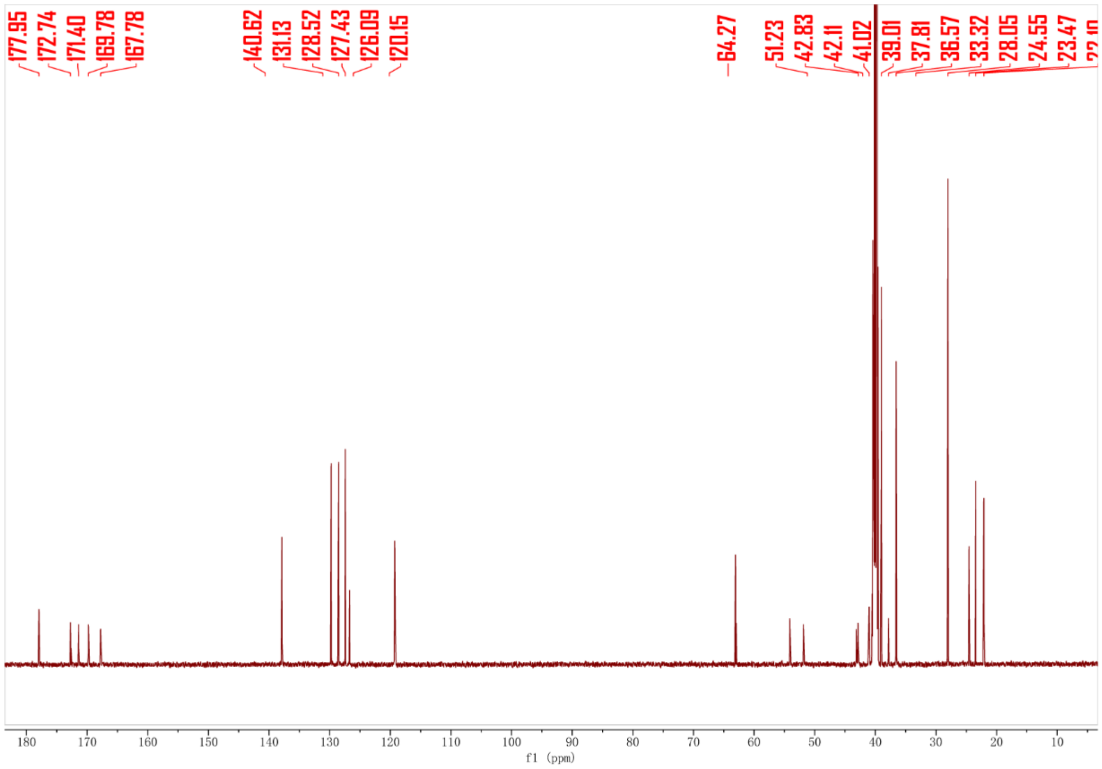

Fig. S24.^{13}C NMR spectrum of compound 15 in DMSO-d$_6$.

Elmt	Val.	Min	Max	Use Adduct
H	1	49	49	H
C	4	37	37	Na
N	3	5	5	K
O	2	6	6	NH4

Error Margin (ppm):	20	DBE Range:	-100.0 - 200.0	Electron Ions:	odd
HC Ratio:	0.0 - 100.0	Apply N Rule:	yes	Use MSn Info:	no
Max Isotopes:	all	Isotope RI (%):	1.00	Isotope Res:	10000
MSn Iso RI (%):	75.00	MSn Logic Mode:	OR	Max Results:	1000

Event#: 1 MS(E+) Ret. Time : 0.823 Scan# : 123

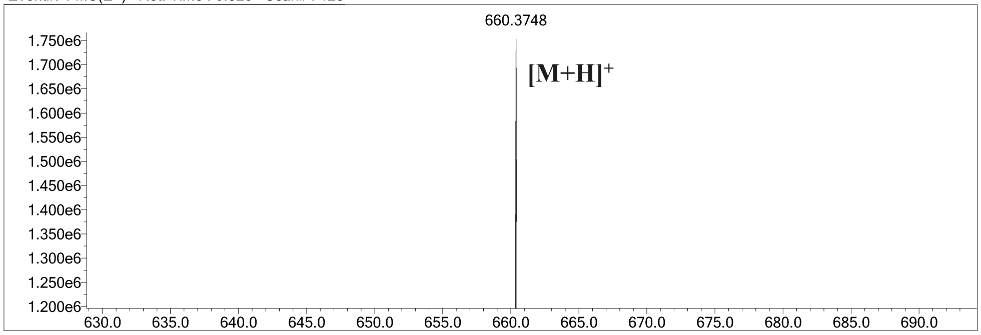

Measured region for 660.3748 m/z

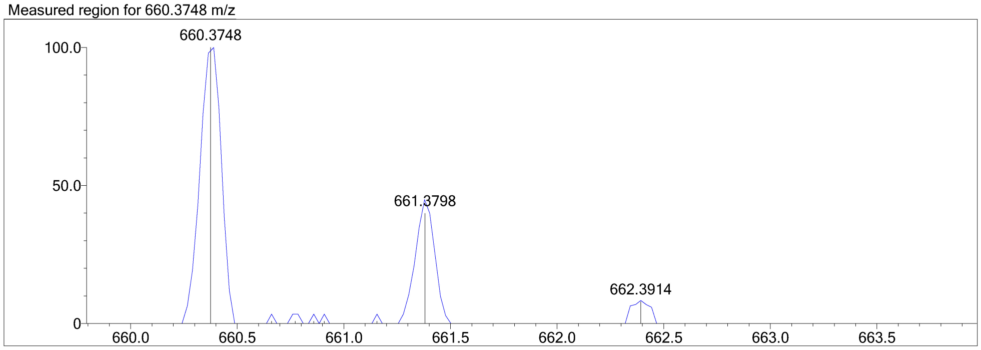

C37 H49 N5 O6 [M+H]+ : Predicted region for 660.3756 m/z

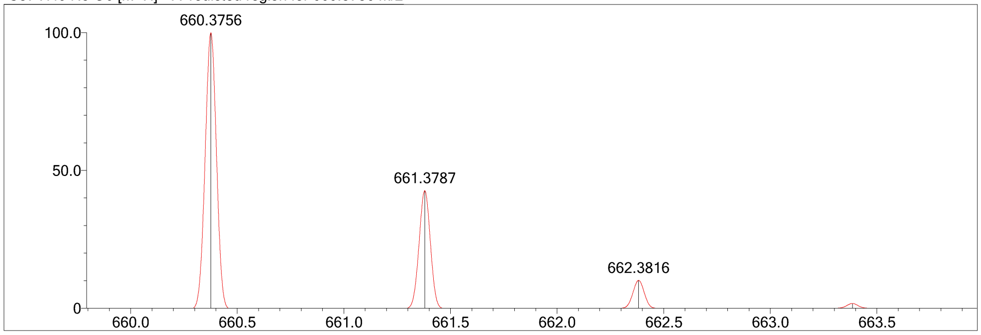

Rank	Score	Formula (M)	Ion	Meas. m/z	Pred. m/z	Df. (mDa)	Df. (ppm)	Iso	DBE
1	84.03	C37 H49 N5 O6	[M+H]+	660.3748	660.3756	-0.8	-1.21	84.48	16.0

Fig. S25. HRMS spectrum of compound 15.

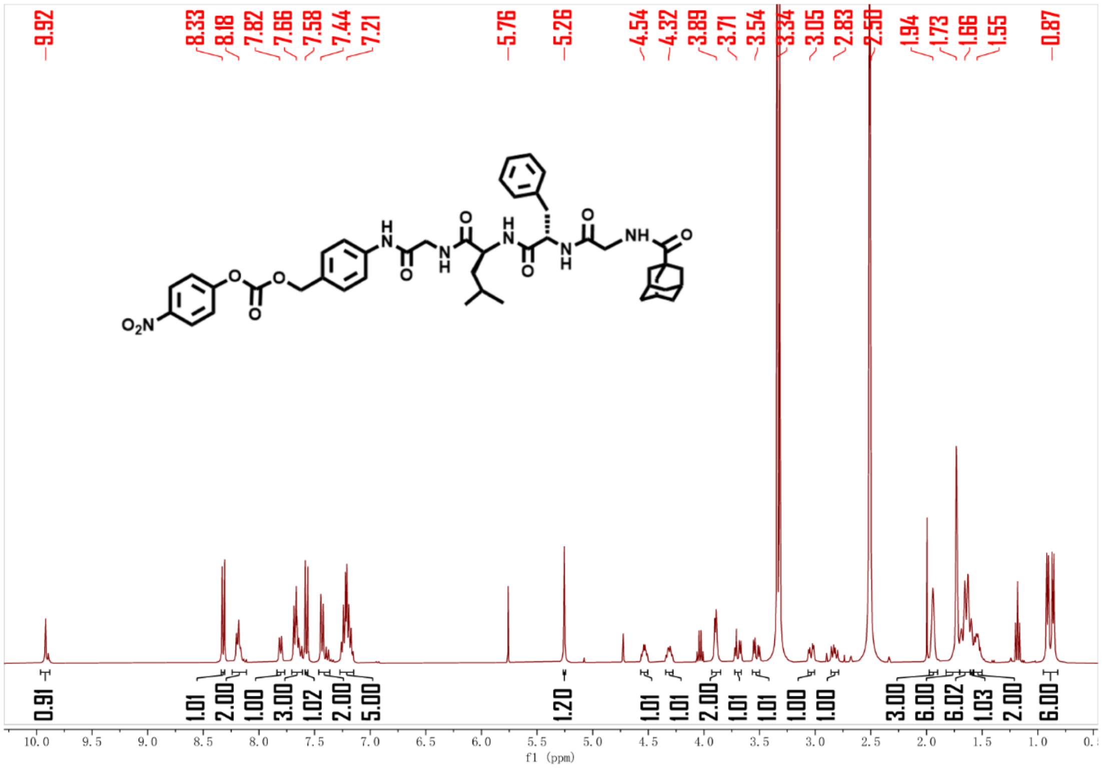

Fig. S26. ^{1}H NMR spectrum of compound 16 in DMSO-d_6.

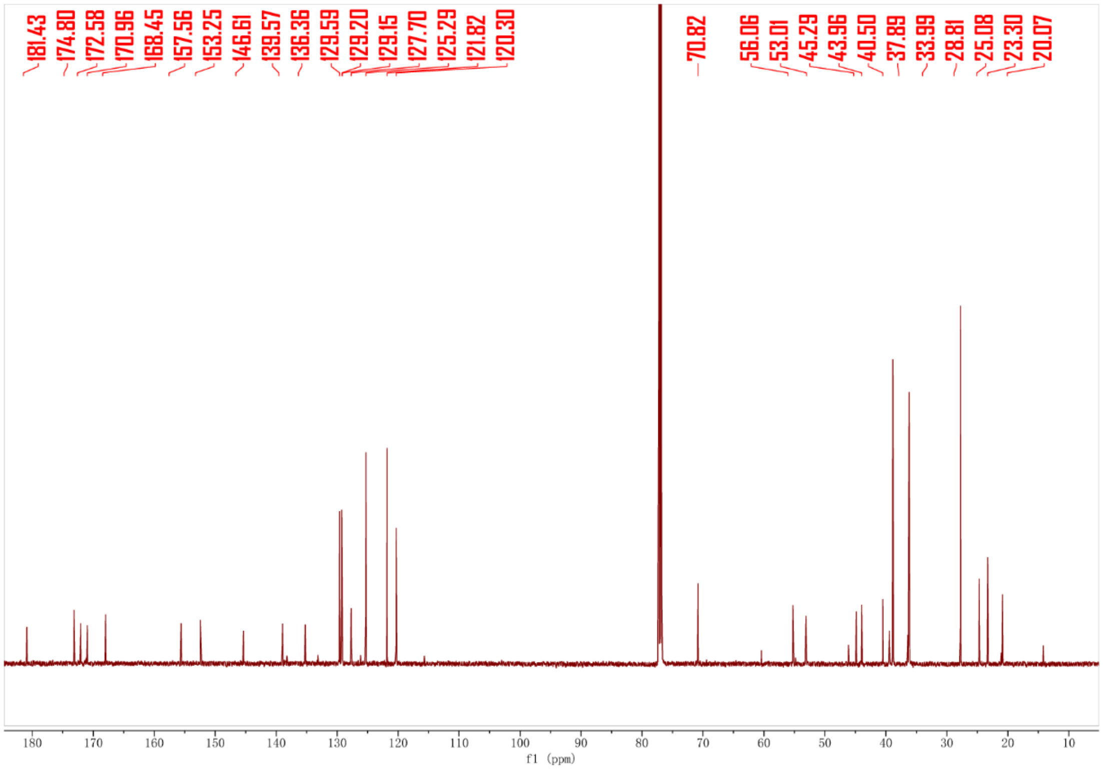

Fig. S27. ^{13}C NMR spectrum of compound 16 in $CDCl_3$.

Elmt	Val.	Min	Max
H	1	52	52
C	4	44	44
N	3	6	6
O	2	10	10

Use Adduct
H
Na
K
NH4

Error Margin (ppm):	20	DBE Range:	-100.0 - 200.0	Electron Ions:	odd
HC Ratio:	0.0 - 100.0	Apply N Rule:	yes	Use MSn Info:	no
Max Isotopes:	all	Isotope RI (%):	1.00	Isotope Res:	10000
MSn Iso RI (%):	75.00	MSn Logic Mode:	OR	Max Results:	1000

Event#: 1 MS(E+) Ret. Time : 0.983 Scan# : 147

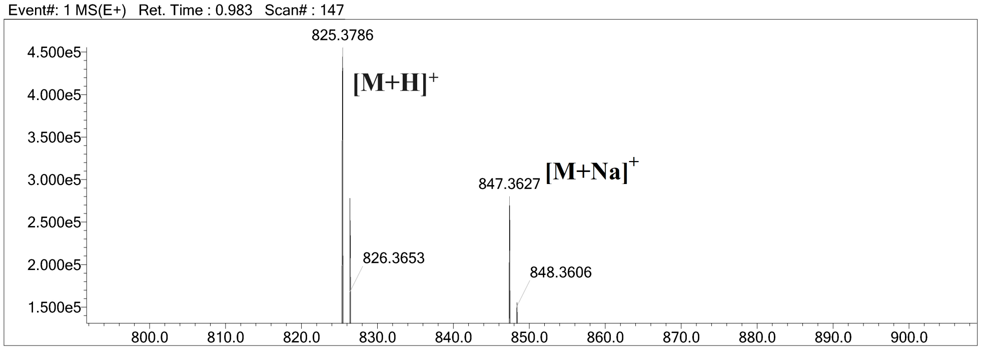

Measured region for 825.3786 m/z

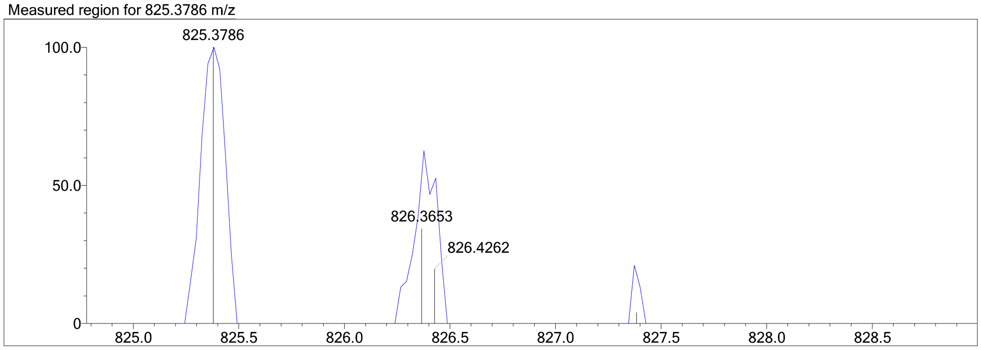

C44 H52 N6 O10 [M+H]+ : Predicted region for 825.3818 m/z

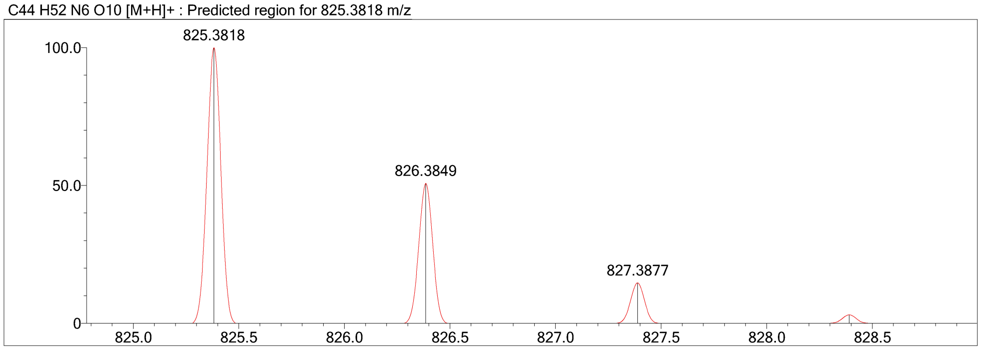

Rank	Score	Formula (M)	Ion	Meas. m/z	Pred. m/z	Df. (mDa)	Df. (ppm)	Iso	DBE
1	59.60	C44 H52 N6 O10	[M+H]+	825.3786	825.3818	-3.2	-3.88	64.22	22.0

Fig. S28. HRMS spectrum of compound 16.

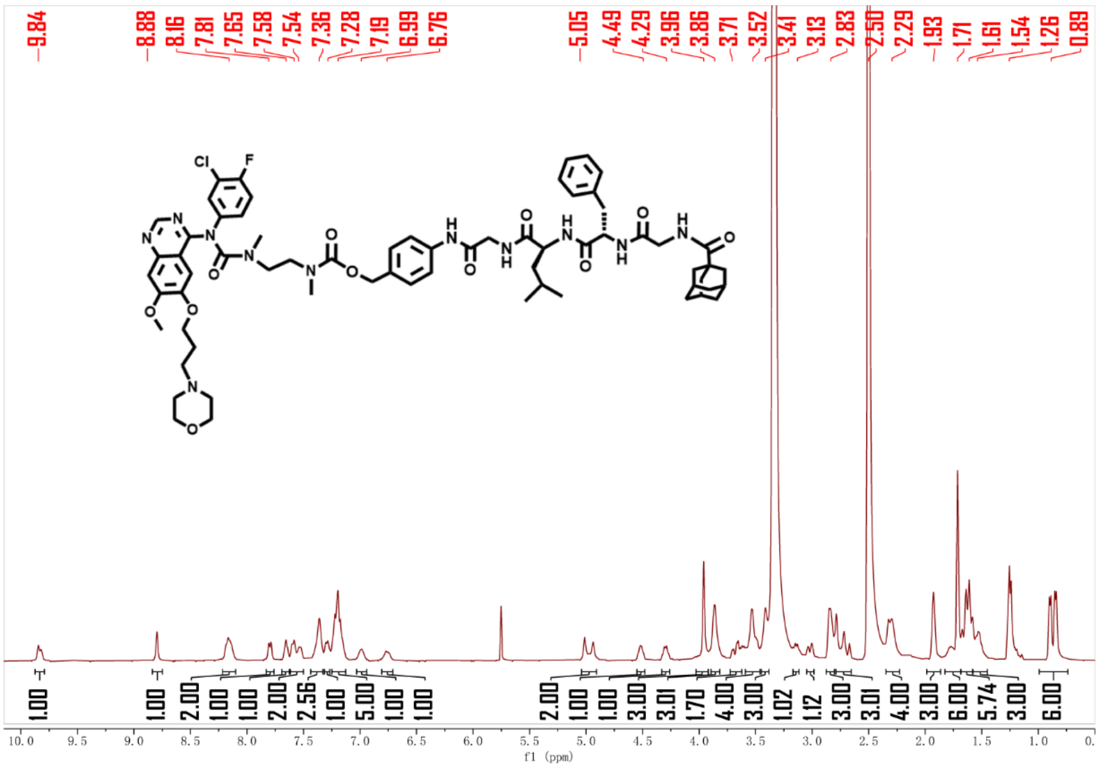

Fig. S29. ^{1}H NMR spectrum of Ada-GFLG-GEF in DMSO-d_6.

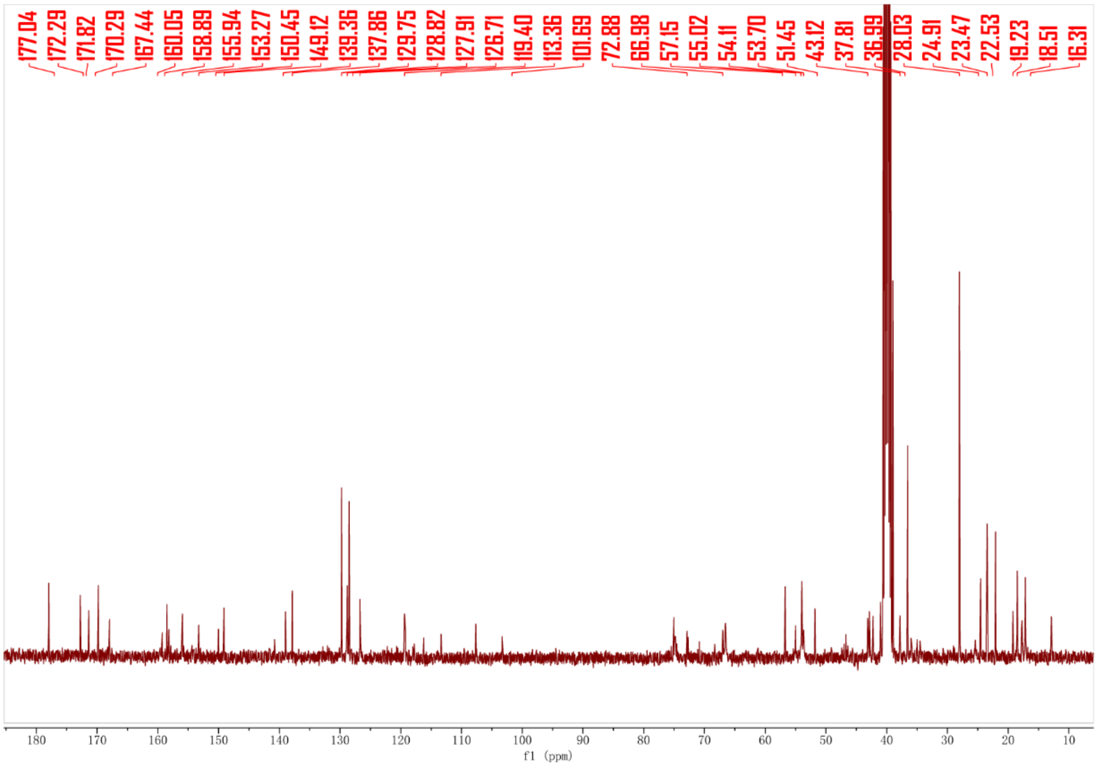

Fig. S30. ^{13}C NMR spectrum of Ada-GFLG-GEF in DMSO-d_6.

Elmt	Val.	Min	Max
H	1	81	81
C	4	65	65
N	3	11	11
O	2	11	11

Elmt	Val.	Min	Max
F	1	1	1
Cl	1	1	1

Use Adduct
H
Na
K
NH4

Error Margin (ppm): 20
HC Ratio: 0.0 - 100.0
Max Isotopes: all
MSn Iso RI (%): 75.00

DBE Range: -100.0 - 200.0
Apply N Rule: yes
Isotope RI (%): 1.00
MSn Logic Mode: OR

Electron Ions: odd
Use MSn Info: no
Isotope Res: 10000
Max Results: 1000

Event#: 1 MS(E+) Ret. Time : 0.837 Scan# : 125

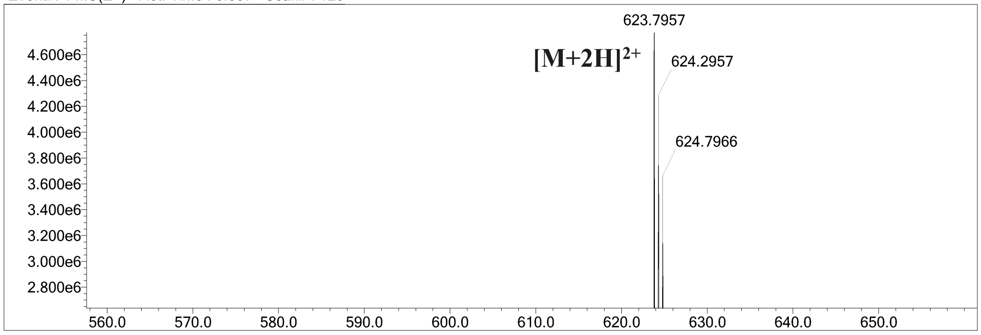

Measured region for 623.7957 m/z

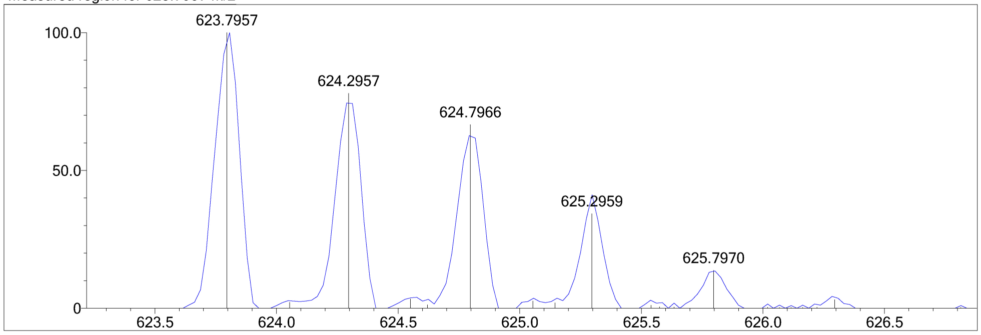

C65 H81 N11 O11 F Cl [M+2H]2+ : Predicted region for 623.7968 m/z

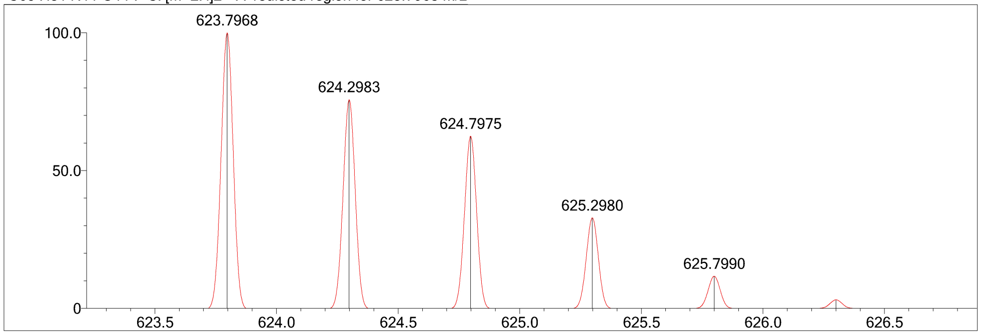

Rank	Score	Formula (M)	Ion	Meas. m/z	Pred. m/z	Df. (mDa)	Df. (ppm)	Iso	DBE
1	85.05	C65 H81 N11 O11 F Cl	[M+2H]2+	623.7957	623.7968	-1.1	-1.76	86.70	30.0

Fig. S31. HRMS spectrum of Ada-GFLG-GEF.

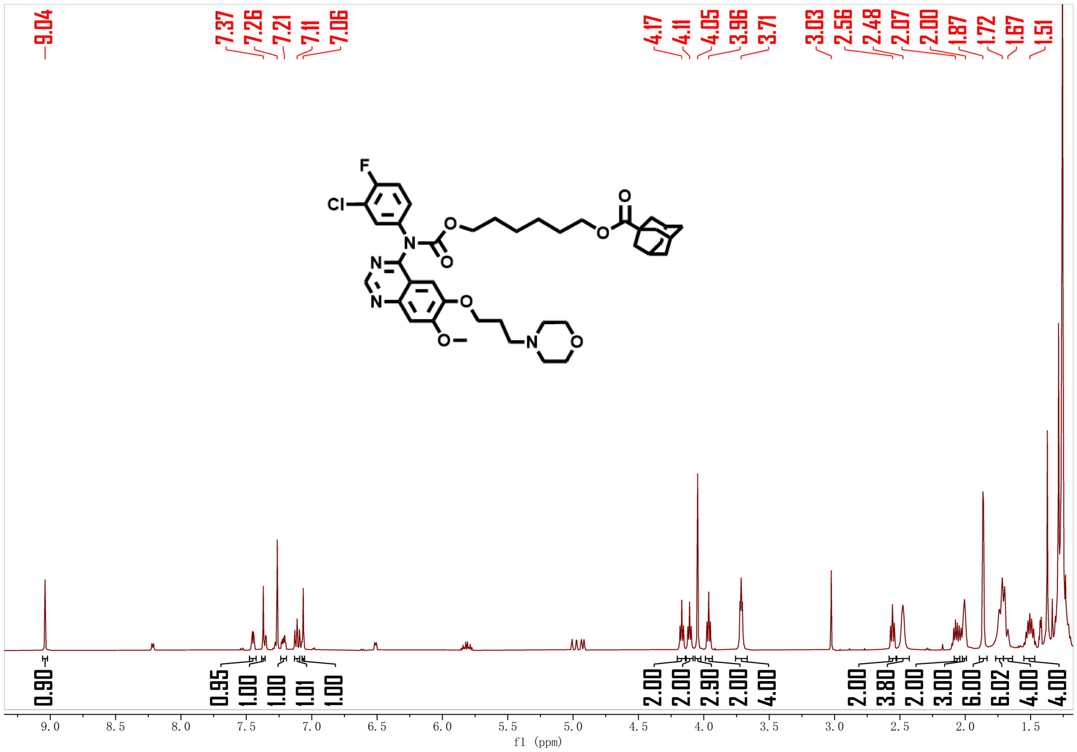

Fig. S32. ^{1}H NMR spectrum of Ada-HE-GEF in $CDCl_3$.

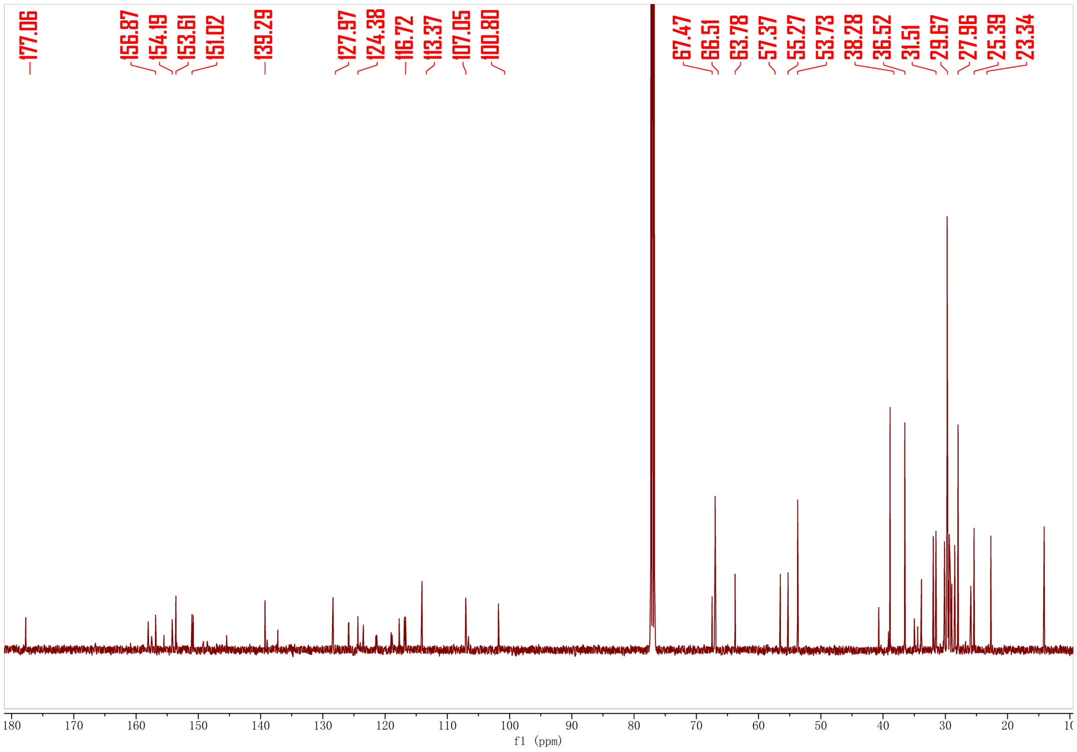

Fig. S33. ^{13}C NMR spectrum of Ada-HE-GEF in $CDCl_3$.

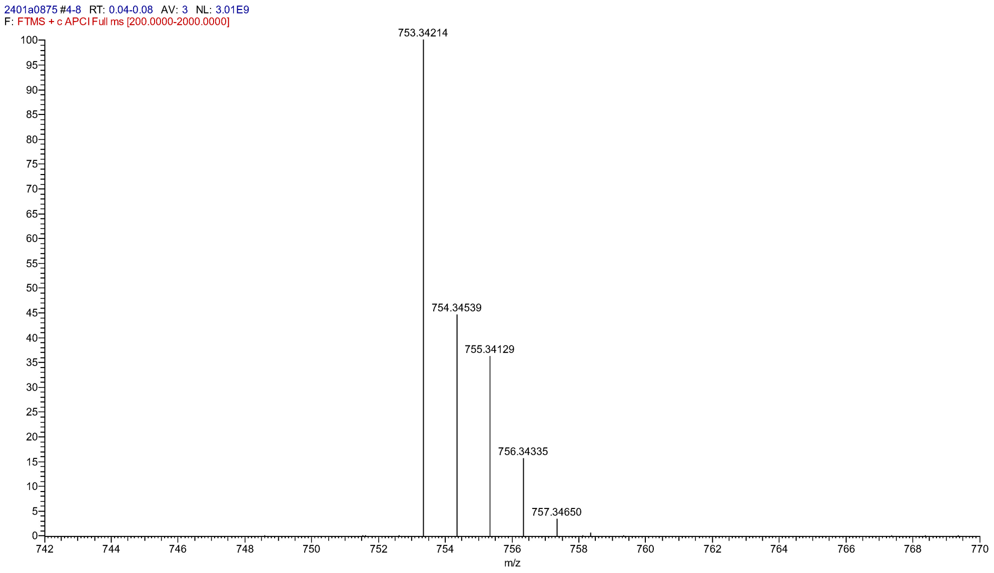

SPECTRUM - simulation :

m/z	Theo. Mass	Delta (ppm)	RDB equiv.	Composition
753.34214	753.34248	-0.45	16.5	C40 H51 O7 N4 Cl F

Fig. S34. HRMS spectrum of Ada-HE-GEF.

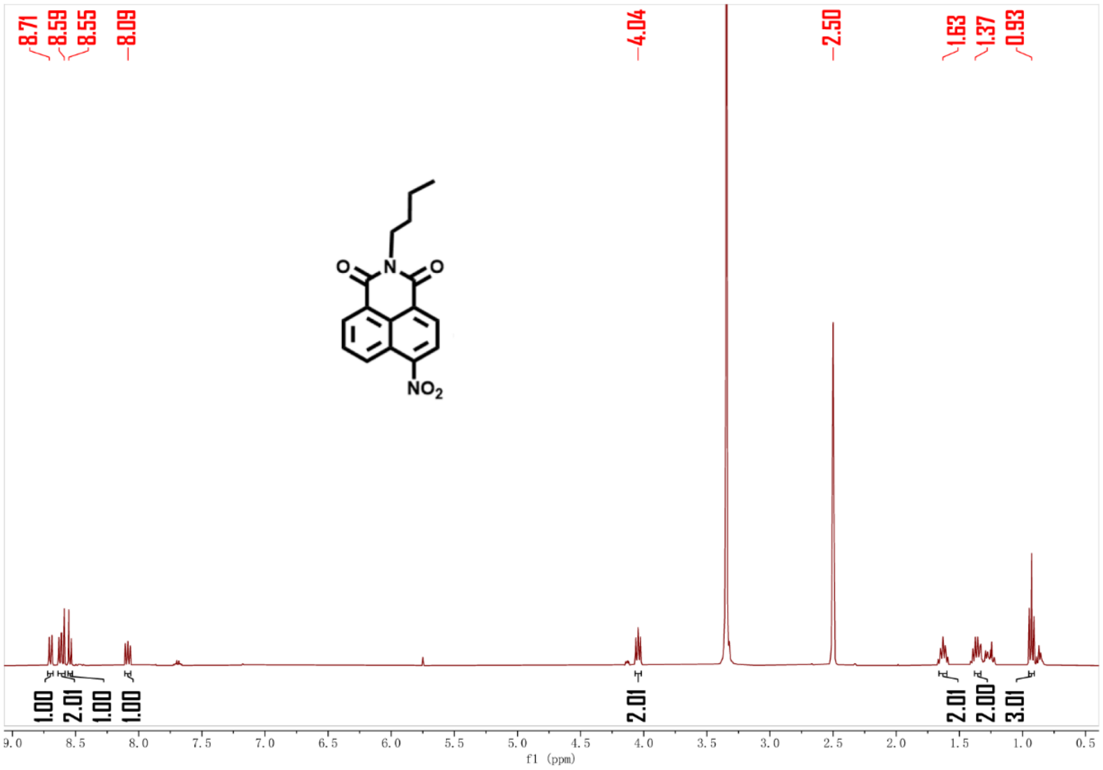

Fig. S35. ^{1}H NMR spectrum of compound 21 in DMSO-d_6.

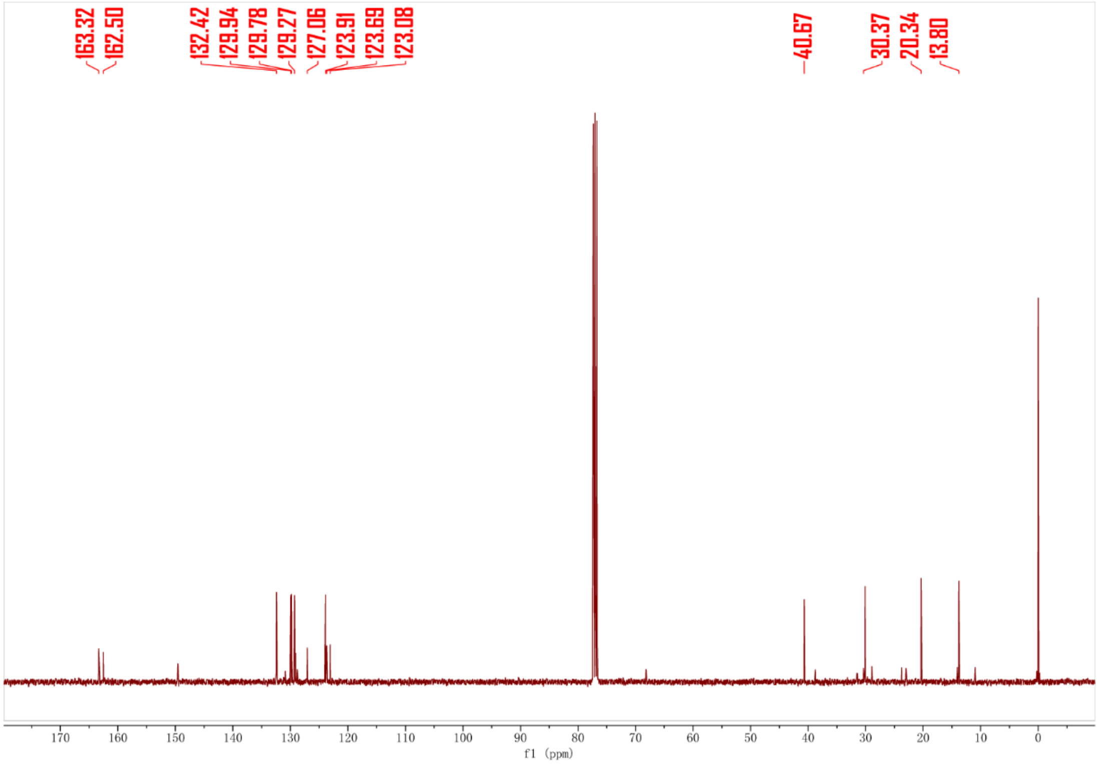

Fig. S36. ^{13}C NMR spectrum of compound 21 in $CDCl_3$.

Elmt	Val.	Min	Max
H	1	14	14
C	4	16	16
N	3	0	2
O	2	3	4

Elmt	Val.	Min	Max
S	2	0	0

Use Adduct
H
Na
K
NH4

Error Margin (ppm):	10	DBE Range:	not fixed	Electron Ions:	both
HC Ratio:	unlimited	Apply N Rule:	yes	Use MSn Info:	no
Max Isotopes:	all	Isotope RI (%):	1.00	Isotope Res:	10000
MSn Iso RI (%):	75.00	MSn Logic Mode:	AND	Max Results:	1000

Event#: 1 MS(E+) Ret. Time : 2.637 Scan# : 395

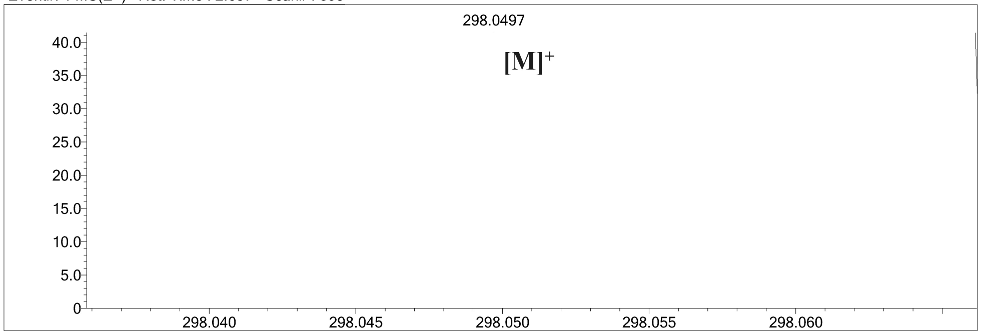

Measured region for 298.0938 m/z

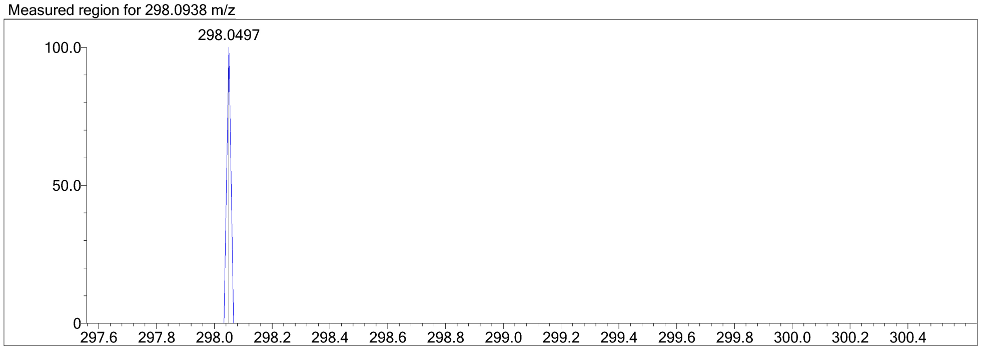

C16 H14 N2 O4 M+ : Predicted region for 298.0948 m/z

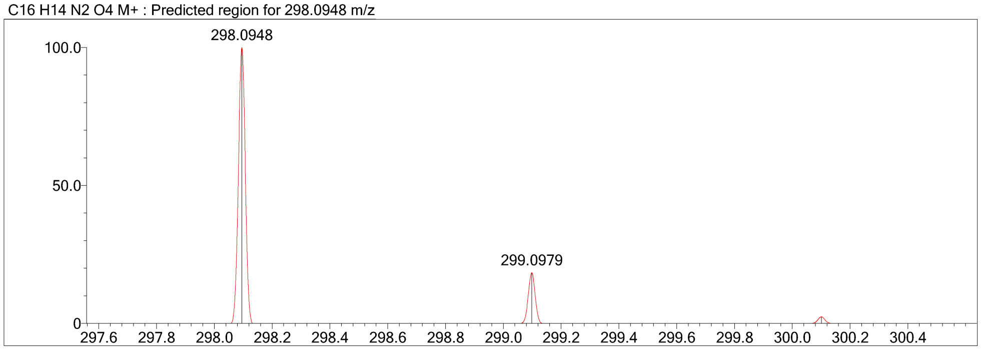

Rank	Score	Formula (M)	Ion	Meas. m/z	Pred. m/z	Df. (mDa)	Df. (ppm)	Iso	DBE
1	0.00	C16 H14 N2 O4	M+	298.0938	298.0948	-1.0	-3.35	0.00	11.0

Fig. S37. HRMS spectrum of compound 21.

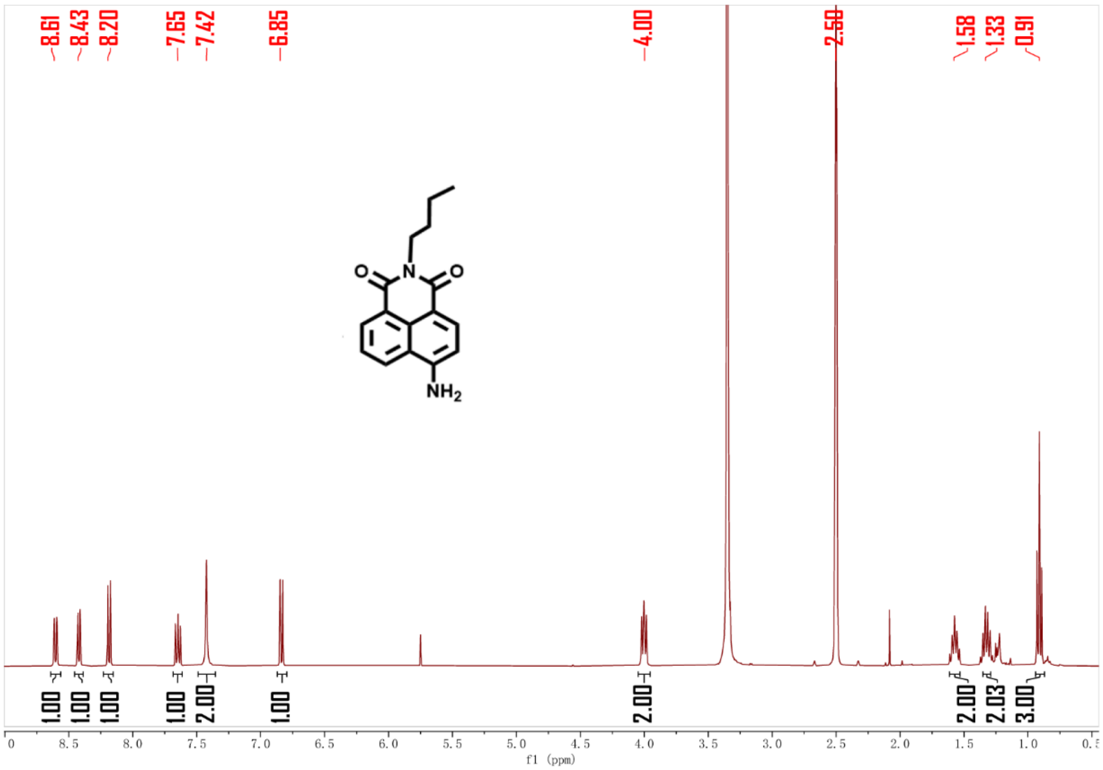

Fig. S38. ^{1}H NMR spectrum of compound 22 in DMSO-d_6.

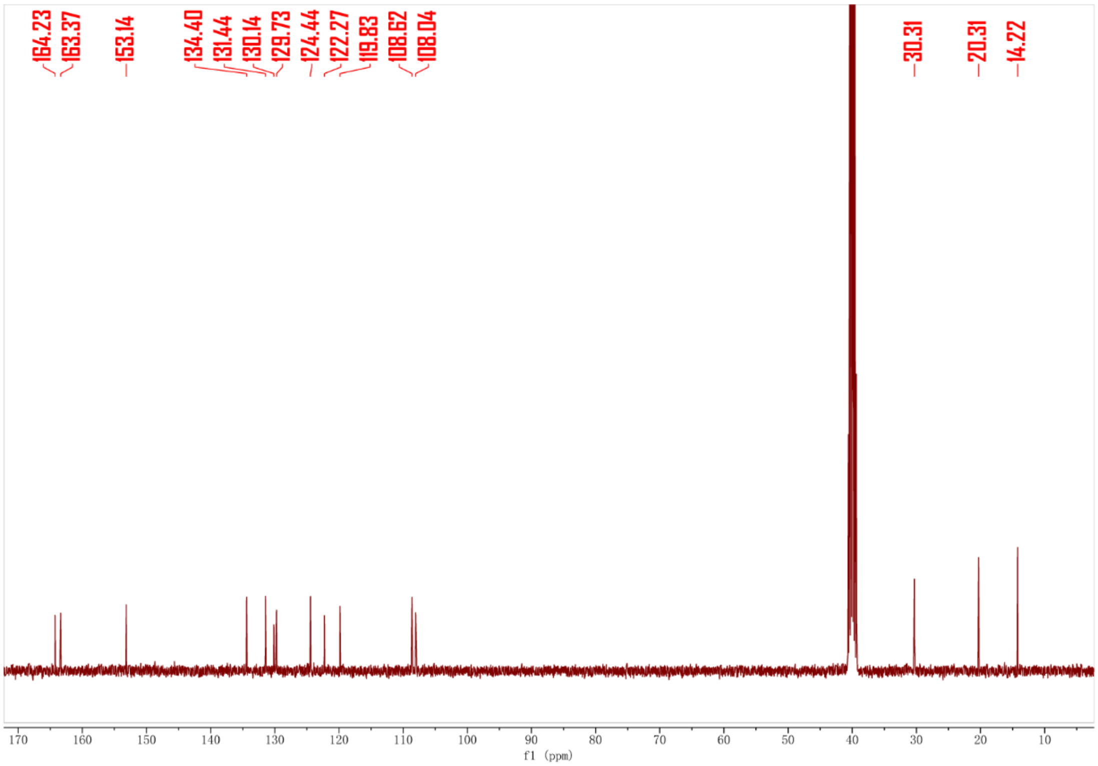

Fig. S39. ^{13}C NMR spectrum of compound 22 in DMSO-d_6.

Elmt	Val.	Min	Max
H	1	16	16
C	4	16	16
N	3	2	2
O	2	2	4

Elmt	Val.	Min	Max
S	2	0	0

Use Adduct
H
Na
K
NH4

Error Margin (ppm):	20	DBE Range:	not fixed	Electron Ions:	both
HC Ratio:	unlimited	Apply N Rule:	yes	Use MSn Info:	no
Max Isotopes:	all	Isotope RI (%):	1.00	Isotope Res:	10000
MSn Iso RI (%):	75.00	MSn Logic Mode:	AND	Max Results:	1000

Event#: 1 MS(E+) Ret. Time : 0.690 Scan# : 103

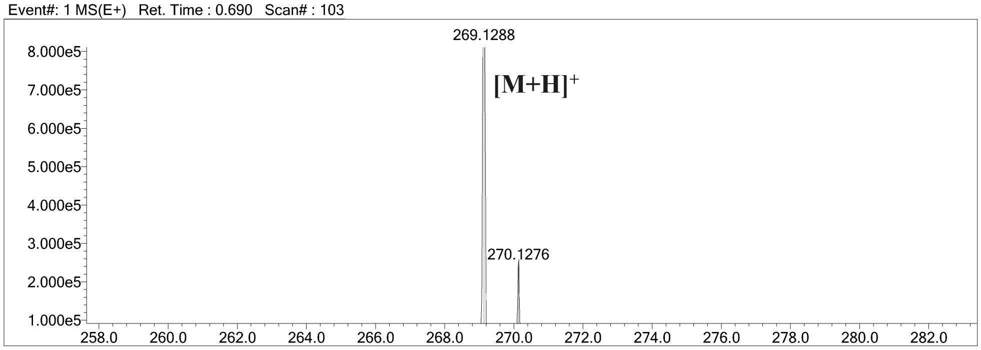

Measured region for 269.1288 m/z

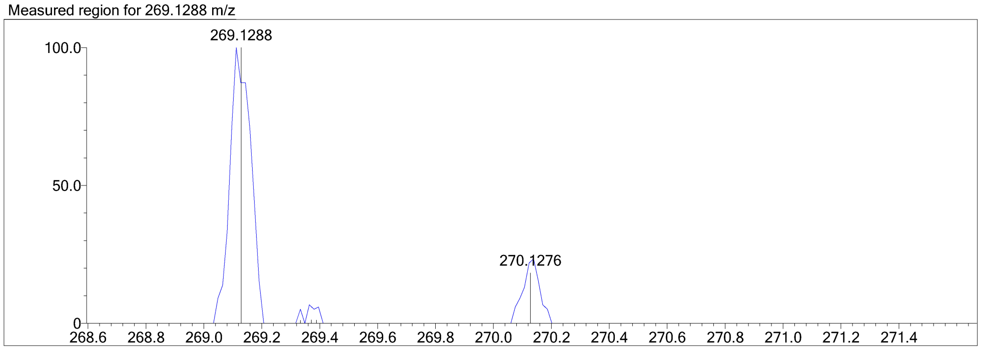

C16 H16 N2 O2 [M+H]+ : Predicted region for 269.1285 m/z

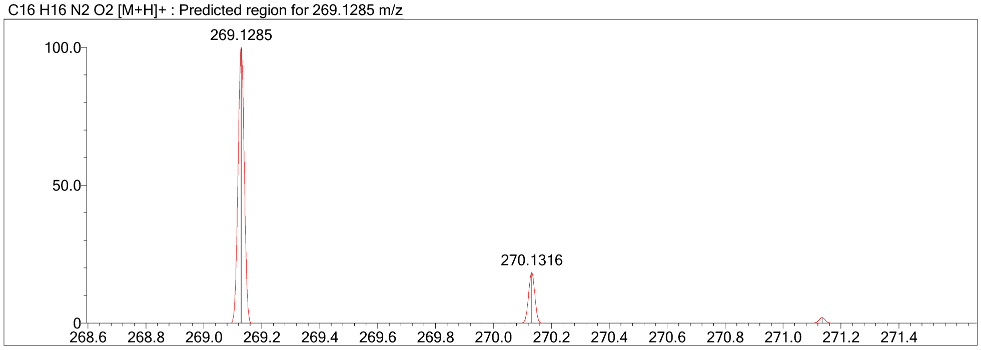

Rank	Score	Formula (M)	Ion	Meas. m/z	Pred. m/z	Df. (mDa)	Df. (ppm)	Iso	DBE
1	35.27	C16 H16 N2 O2	[M+H]+	269.1288	269.1285	0.3	1.11	35.37	10.0

Fig. S40. HRMS spectrum of compound 22.

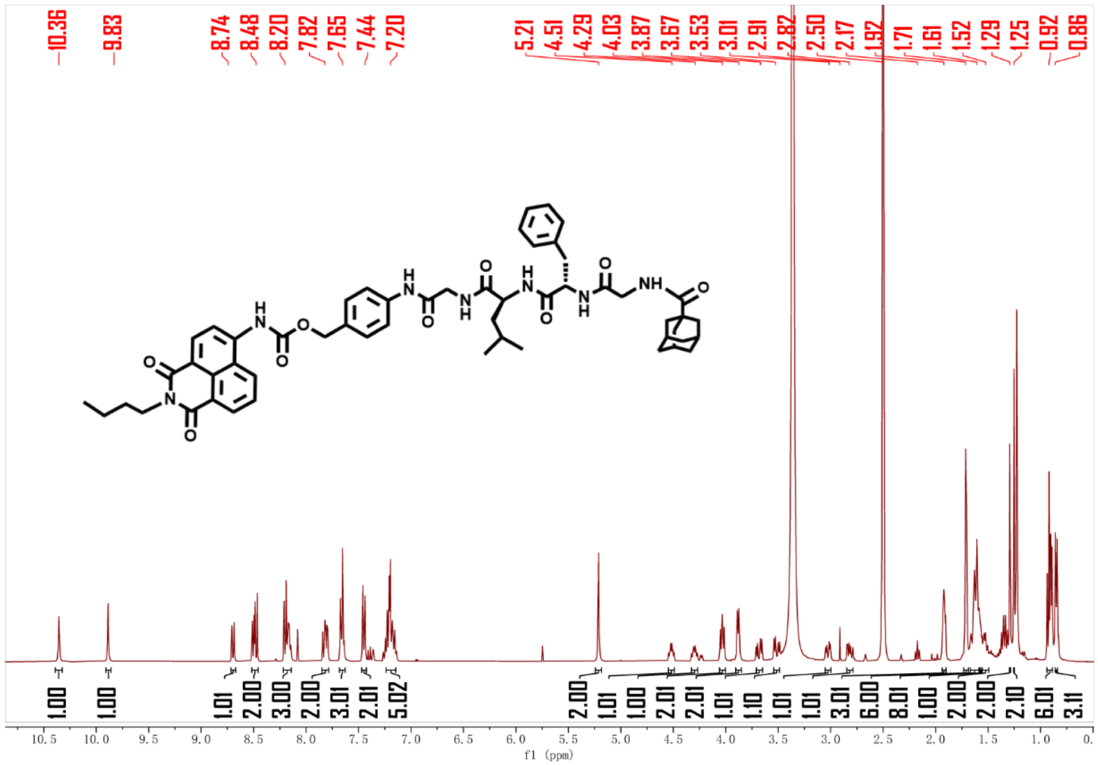

Fig. S41. ^{1}H NMR spectrum of Ada-GFLG-NAP in DMSO-d_6.

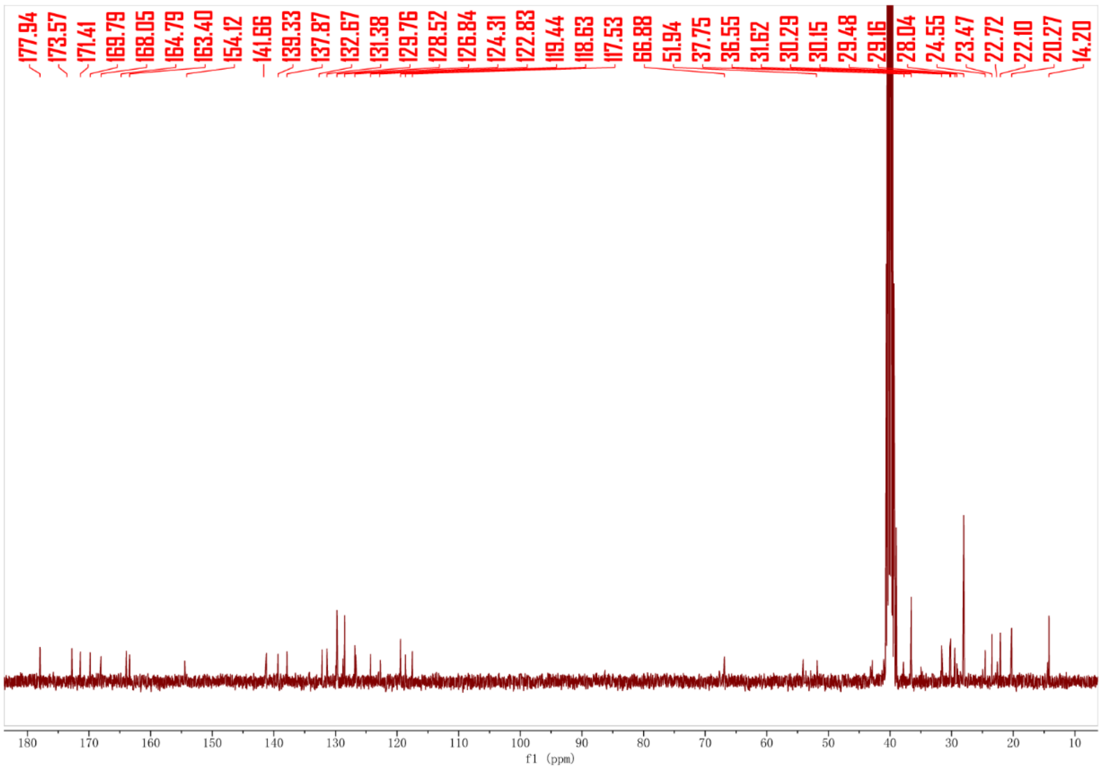

Fig. S42. ^{13}C NMR spectrum of Ada-GFLG-NAP in DMSO-d_6.

Elmt	Val.	Min	Max	Elmt	Val.	Min	Max	Use Adduct
H	1	63	63	S	2	0	0	H
C	4	54	54					Na
N	3	7	7					K
O	2	9	9					NH4

Error Margin (ppm):	20	DBE Range:	not fixed	Electron Ions:	both
HC Ratio:	unlimited	Apply N Rule:	yes	Use MSn Info:	no
Max Isotopes:	all	Isotope RI (%):	1.00	Isotope Res:	10000
MSn Iso RI (%):	75.00	MSn Logic Mode:	AND	Max Results:	1000

Event#: 1 MS(E+) Ret. Time : 0.810 Scan# : 121

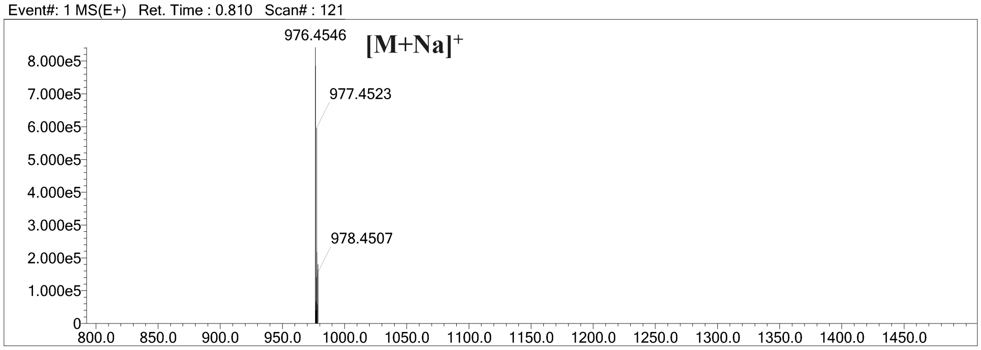

Measured region for 976.4546 m/z

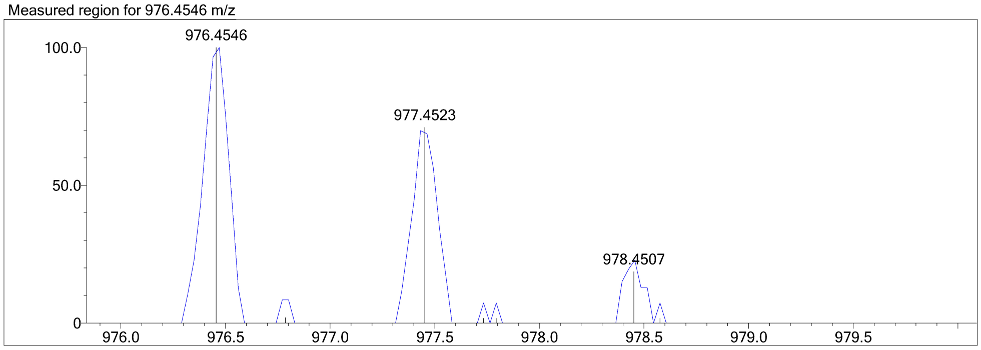

C54 H63 N7 O9 [M+Na]+ : Predicted region for 976.4579 m/z

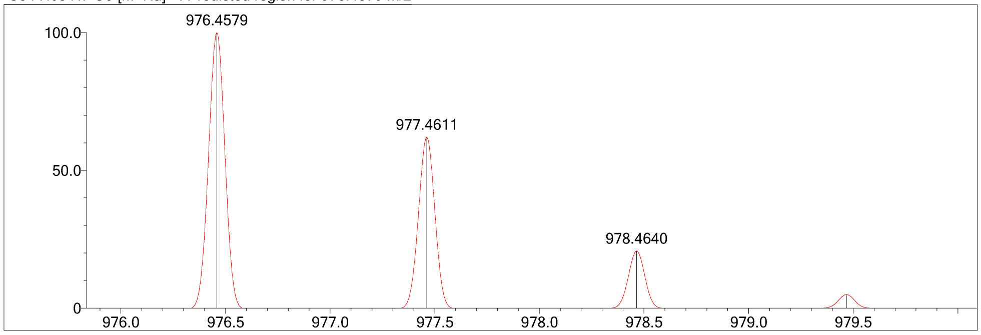

Rank	Score	Formula (M)	Ion	Meas. m/z	Pred. m/z	Df. (mDa)	Df. (ppm)	Iso	DBE
1	62.55	C54 H63 N7 O9	[M+Na]+	976.4546	976.4579	-3.3	-3.38	66.51	27.0

Fig. S43. HRMS spectrum of compound Ada-GFLG-NAP.

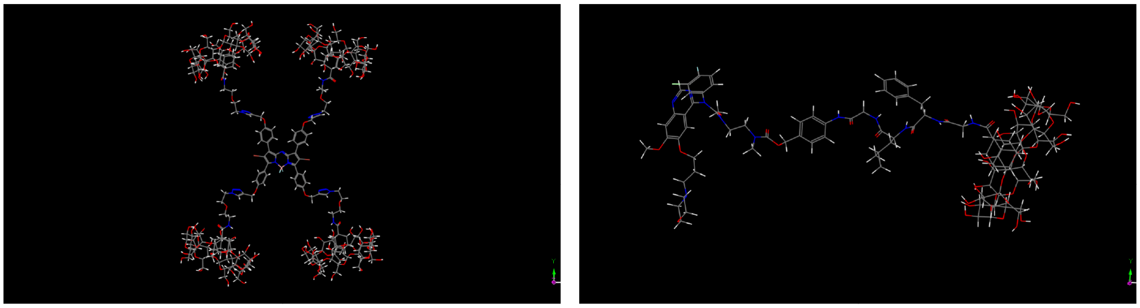

Fig. S44. Molecular simulation results. Complexation between β-CD and **a** Ada-BPY or **b** Ada-GFLG-GEF. To clarify the host-guest interaction between β-CD and adamantane, the complexes were detected by molecular modeling studies. The starting structures of host molecule, guest molecules and the inclusion compounds were built by using Materials Studio 8.0 and the energy was minimized with the same software. All geometry optimizations and energy calculation were performed by using a Universal forcefield. The molecular modeling studies were also performed by using Materials Studio 8.0 to give the computational minimum-energy structures between cyclodextrin and guest molecule. The calculated total energies of inclusion compounds were as follows: β-CD (210.34 kcal/mol); Ada-BPY (516.32 kcal/mol); Ada-GFLG-GEF (232.43 kcal/mol); β-CD⊂Ada-BPY (1221.58 kcal/mol); β-CD⊂Ada-GFLG-GEF (393.63 kcal/mol), indicating that β-CD could form stable complexes with adamantane modified guest molecules.

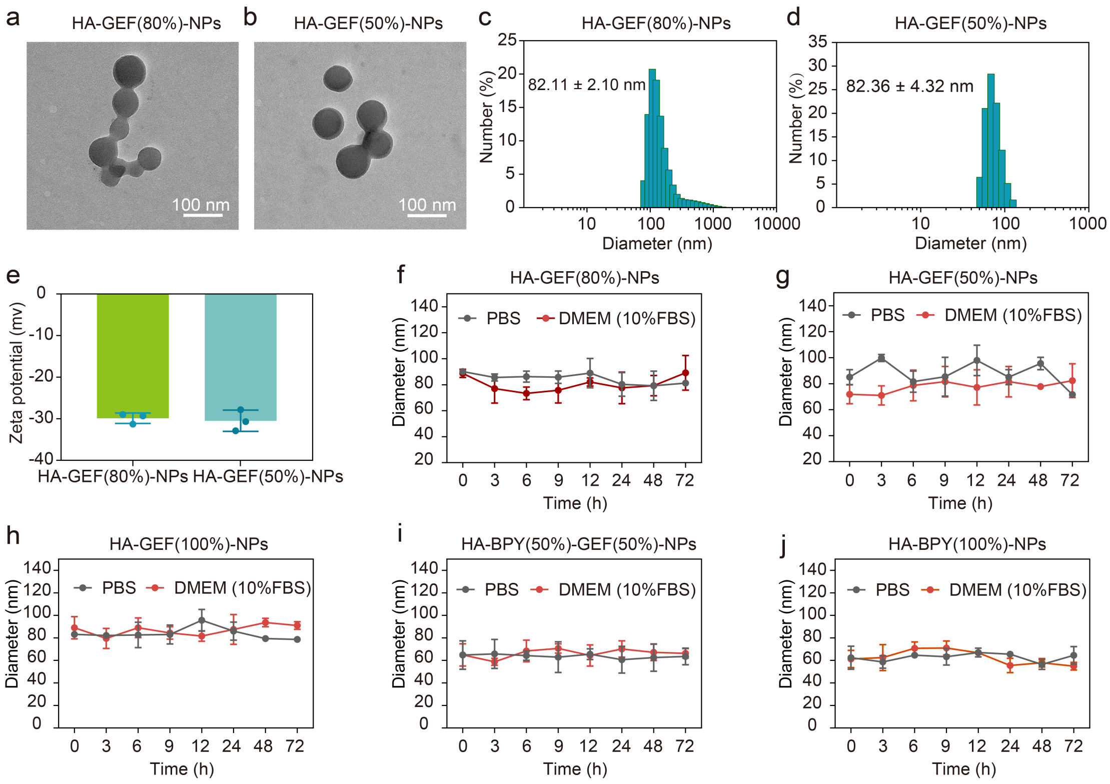

Fig. S45. Characterization of supramolecular prodrug nanoparticles. TEM images of **a** HA-GEF(80%)-NPs and **b** HA-GEF(50%)-NPs. DLS profiles of **c** HA-GEF(80%)-NPs and **d** HA-GEF(50%)-NPs. **e** Zeta potentials of HA-GEF(80%)-NPs and HA-GEF(50%)-NPs. **f-j** Changes in the hydrodynamic size of different nano-formulations in PBS or DMEM (10% FBS). Data are shown as mean ± SD (n = 3).

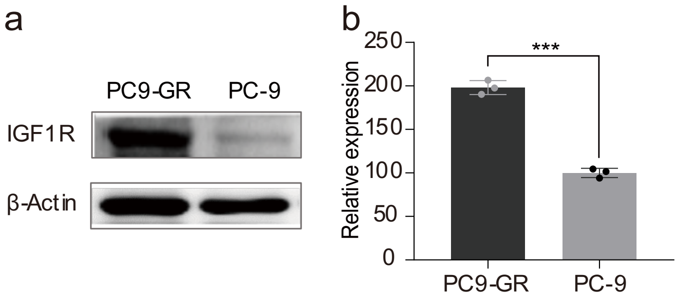

Fig. S46. Western blotting and quantitative analysis. **a** Western blotting images and **b** quantitative analysis of relative IGF1R protein expression in PC9-GR and PC-9 cells. Data are shown as mean ± SD (n = 3). *p<0.05, **p<0.01, and ***p<0.001.

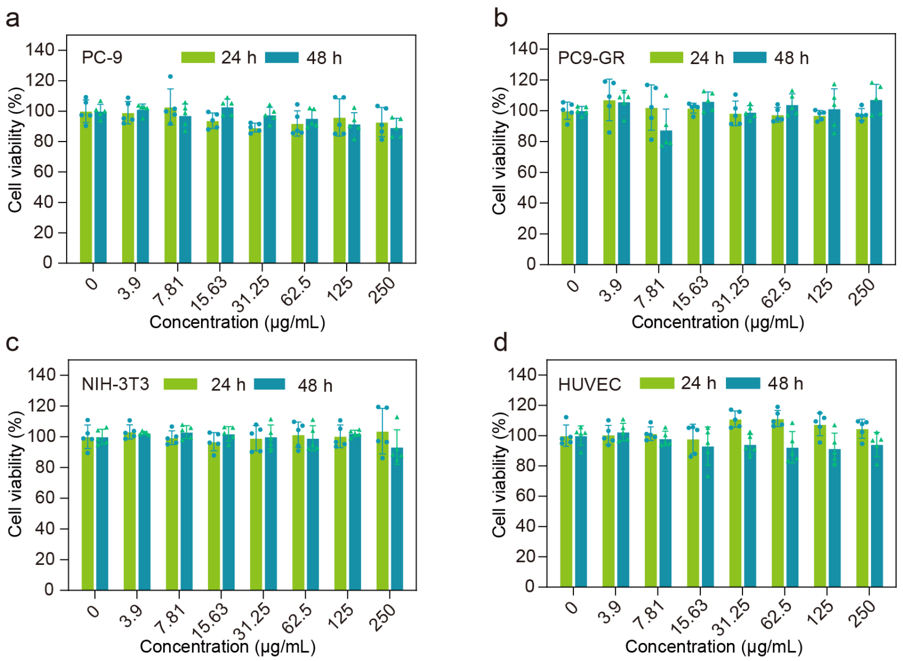

Fig. S47. Cytotoxicity. Cytotoxicity of HA-TK-CD at various concentrations treatment against both tumor cells **a** PC-9 and **b** PC9-GR cells and nontumor cells **c** NIH-3T3 and **d** HUVECs (n = 5).

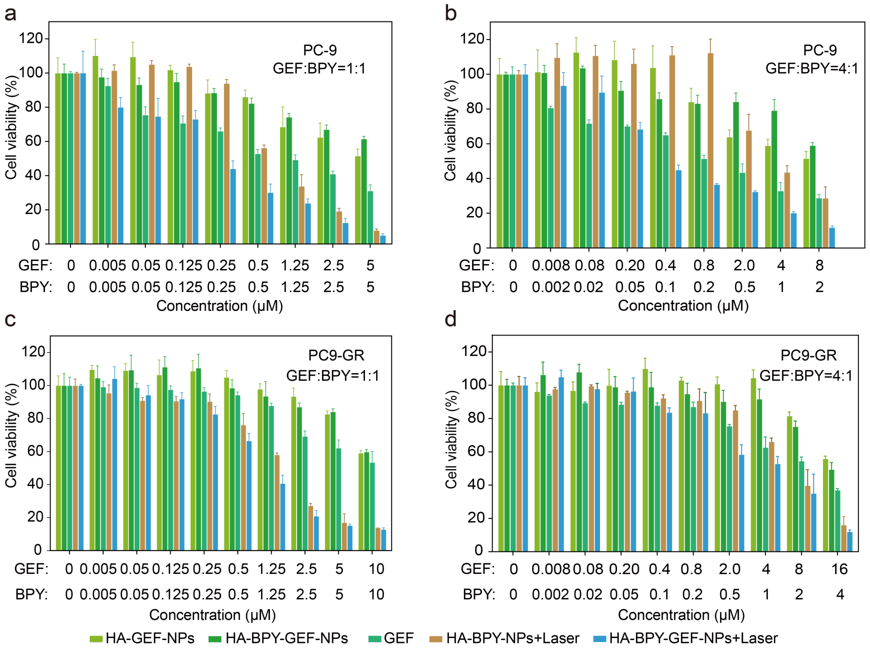

Fig. S48. Cytotoxicity. a-d Cytotoxicity of various formulations at different concentrations treatment on PC-9 and PC9-GR cells. For the laser treated group were irradiated with a 660 nm laser (100 mW/cm^2) for 10 min at 12 h post administration.

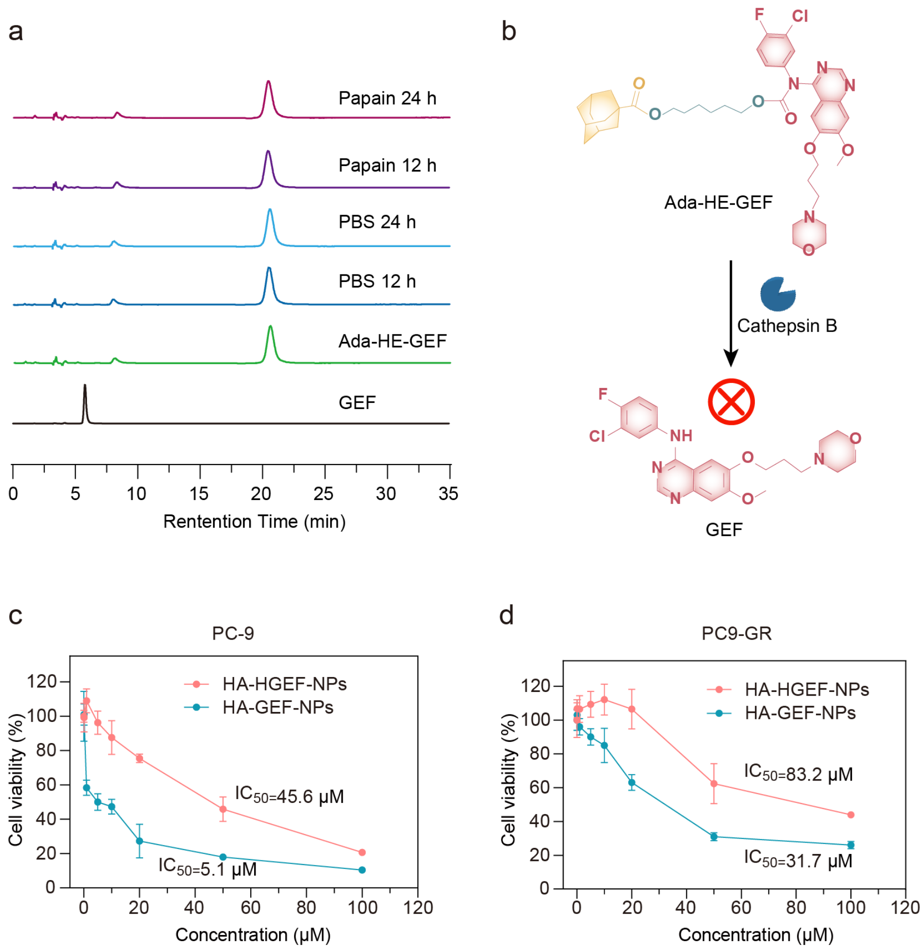

Fig. S49. HPLC profiles and cytotoxicity. **a** HPLC profiles of HA-HGEF-NPs after incubation under different conditions with free GEF and non-cleavable Ada-HE-GEF prodrug used as control. **b** Schematic illustration of non-responsive prodrug activation. **c,d** Cytotoxicity of various formulations at different concentrations upon treatment on PC-9 and PC9-GR cells.

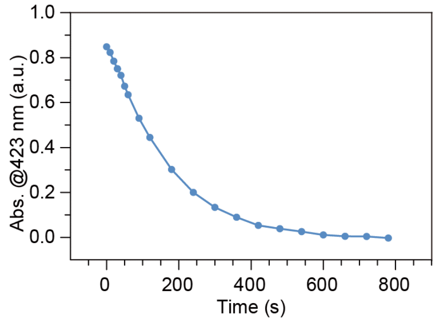

Fig. S50. ROS generation ability of HA-BPY-NPs. DPBF absorbance at 423 nm gradually quenched in HA-BPY-NPs solutions under laser irradiation (660 nm, 20 mW/cm^2).

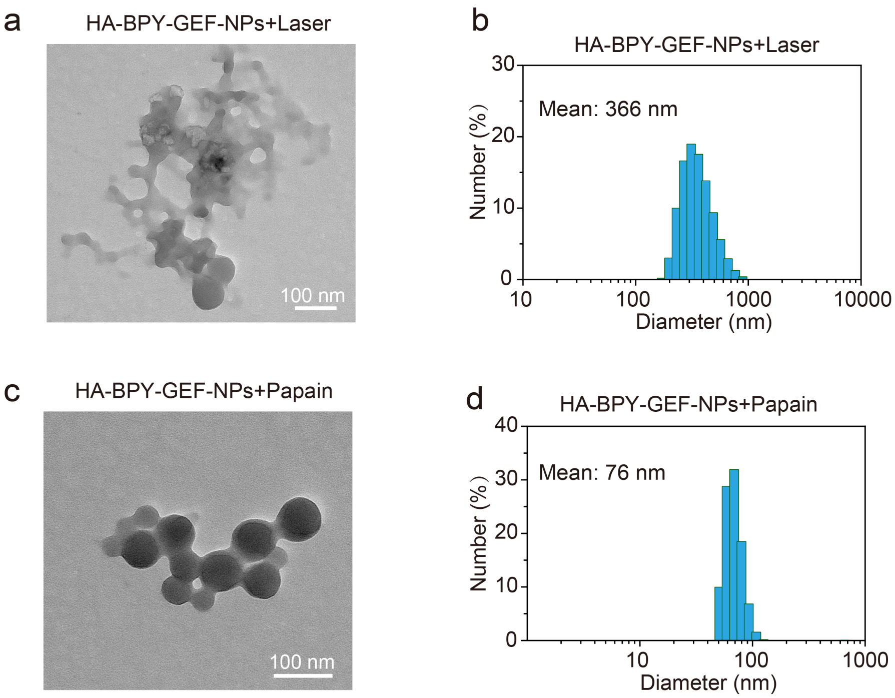

Fig. S51. Characterization of HA-BPY-GEF-NPs in different conditions. a,b TEM image and DLS profile of HA-BPY-GEF-NPs under 660 nm laser irradiation (100 mW/cm^2, 10 min). **c,d** TEM image and DLS profile of HA-BPY-GEF-NPs incubated with papain (10 μg/mL) for 12 h.

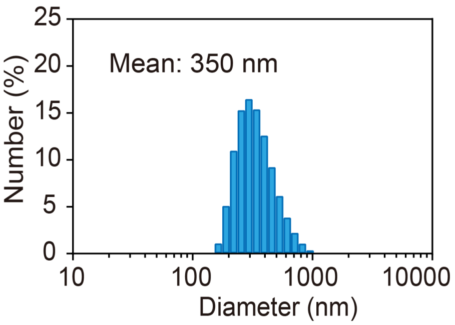

Fig. S52. DLS testing result of HA-BPY-GEF-NPs after NIR irradiation (660 nm, 100 mW/cm^2) for 10 min followed by further incubation with papain (10 μg/mL) for 12 h.

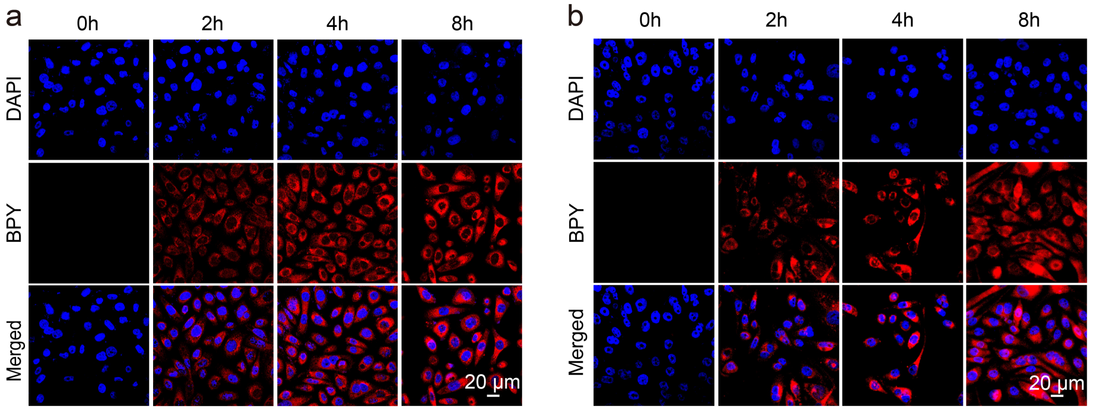

Fig. S53. Cellular uptake of HA-BPY-GEF-NPs. CLSM images of **a** PC-9 and **b** PC9-GR cells incubated with HA-BPY-GEF-NPs (10 μM) for different time, respectively. Red fluorescence indicated Ada-BPY in HA-BPY-GEF-NPs, and blue fluorescence indicated DAPI that was used to stain the nucleus. Red channel: λ_{ex} = 650 nm, and blue channel: λ_{ex} = 405 nm. Scale bars: 20 μm.

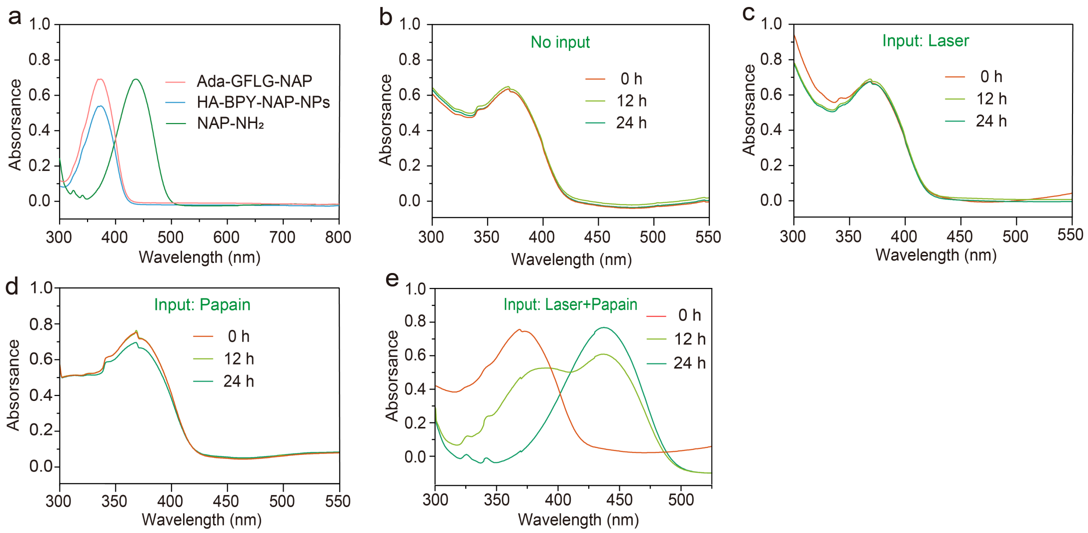

Fig. S54. Response mechanism of probe. a Absorption spectrum of Ada-GFLG-NAP, HA-BPY-NAP-NPs, and $NAP-NH_2$. **b-e** Absorption spectrum of HA-BPY-NAP-NPs nanoprobe in different conditions with incubation for 0 h, 12 h, and 24 h, respectively.

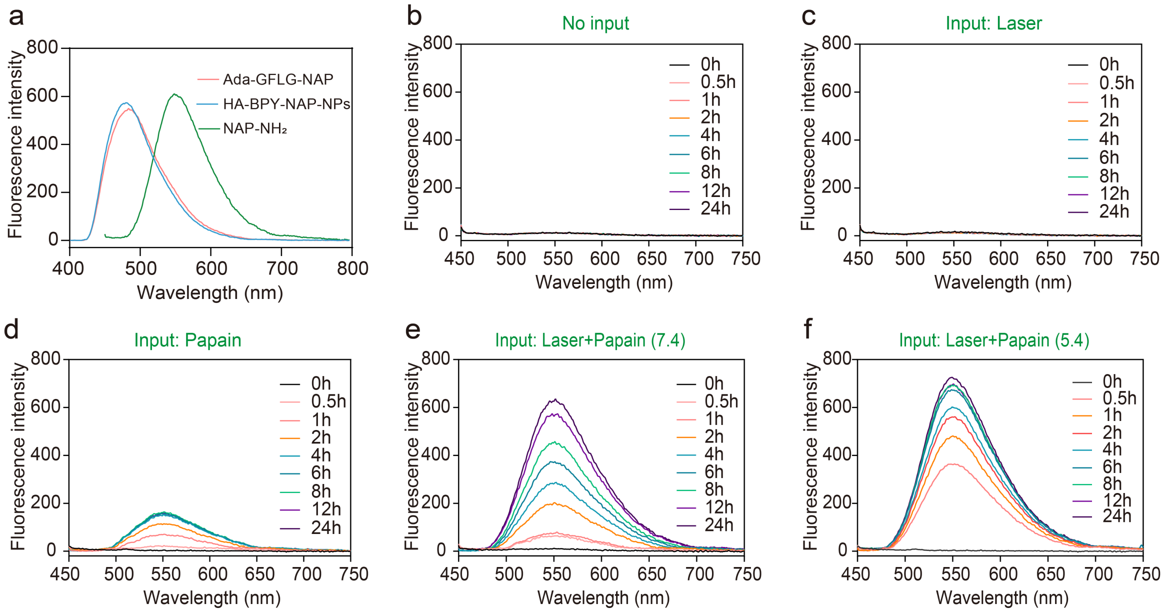

Fig. S55. Responsiveness of probe. a Fluorescence spectrum of Ada-GFLG-NAP (λ_{ex} = 375 nm), HA-BPY-GEF-NPs (λ_{ex} = 375 nm), and $NAP\text{-}NH_2$ (λ_{ex} = 435 nm). **b-f** Fluorescence spectra of HA-BPY-NAP-NPs nanoprobe with NIR photoirradiation (660 nm, 100 mW/cm^2) for 10 min followed by further incubation with papain (10 μg/mL) for different time in different conditions (λ_{ex} = 435 nm).

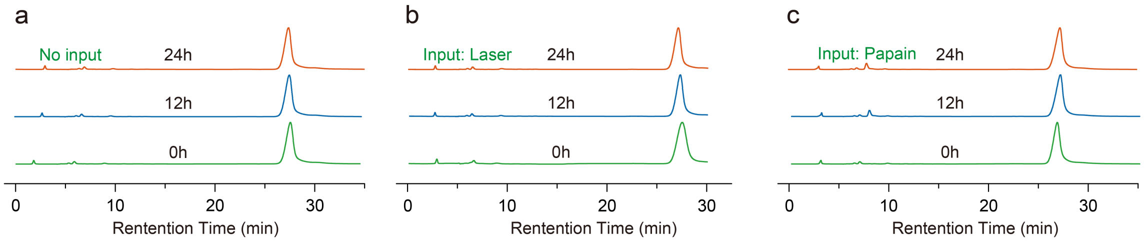

Fig. S56. HPLC profiles. a-c HPLC profiles of HA-BPY-NAP-NPs nanoprobe under different conditions at 0 h, 12 h, and 24 h, respectively.

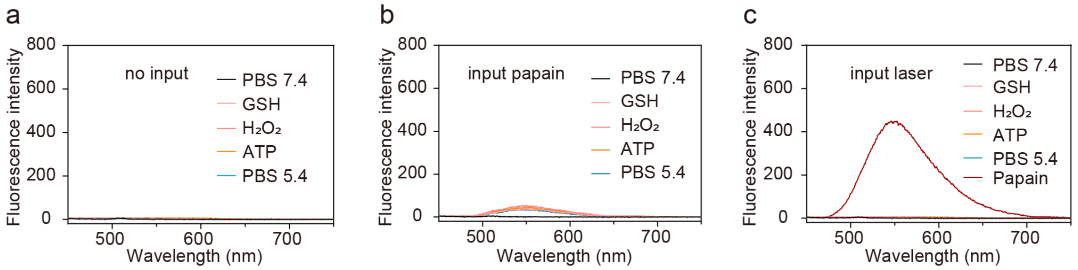

Fig. S57. Selectivity of nanoprobe. a Fluorescence spectrum of HA-BPY-NAP-NPs nanoprobe toward PBS (pH 7.4), 5 mM GSH, 100 µM H_2O_2, 100 µM ATP, and PBS (pH 5.4). **b** Fluorescence spectrum of HA-BPY-NAP-NPs nanoprobe toward PBS (pH 7.4), 5 mM GSH, 100 µM H_2O_2, 100 µM ATP, and PBS (pH 5.4) under papain (10 µg/mL) in PBS. **c** Fluorescence spectrum of HA-BPY-NAP-NPs nanoprobe toward PBS (pH 7.4), 5 mM GSH, 100 µM H_2O_2, 100 µM ATP, PBS (pH 5.4), and Papain (10 µg/mL) in PBS under laser photoirradiation (660 nm, 100 mW/cm^2). (λex = 435 nm).

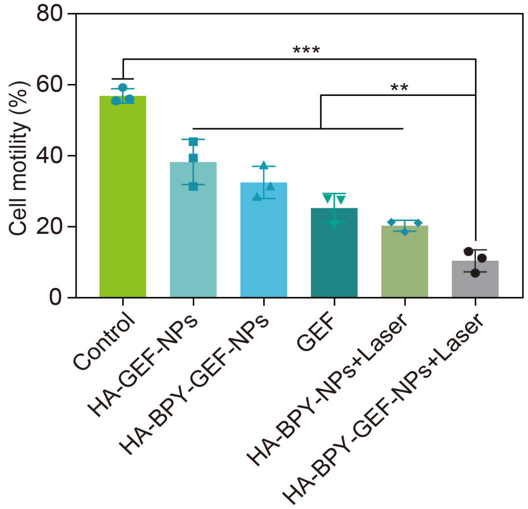

Fig. S58. Quantitative analysis of the cell motility. Data are shown as mean ± SD (n = 3). *p<0.05, **p<0.01, and ***p<0.001.

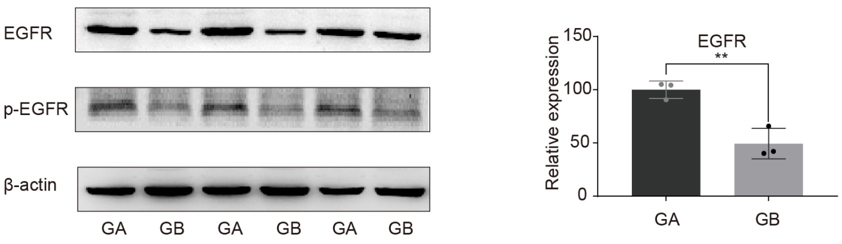

Fig. S59. Western blotting images of proteins in PC9-GR cells after different treatments. Quantification data. GA: HA-BPY-GEF-NPs, GB: HA-BPY-GEF-NPs+Laser. Data are shown as mean ± SD (n = 3). *p<0.05, **p<0.01, and ***p<0.001.

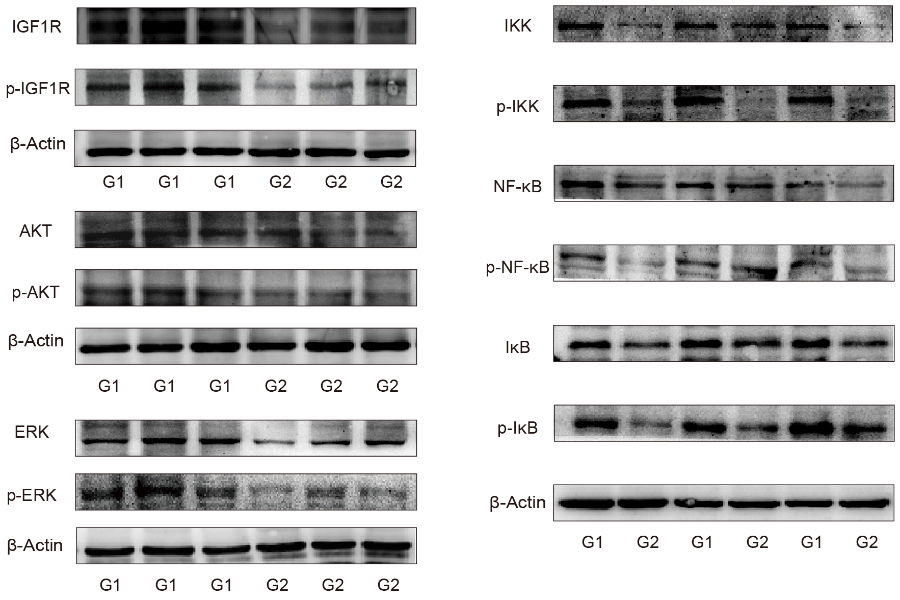

Fig. S60. Western blotting images of proteins expression in PC9-GR cells after different treatments. G1: Control, G2: HA-BPY-GEF-NPs+Laser.

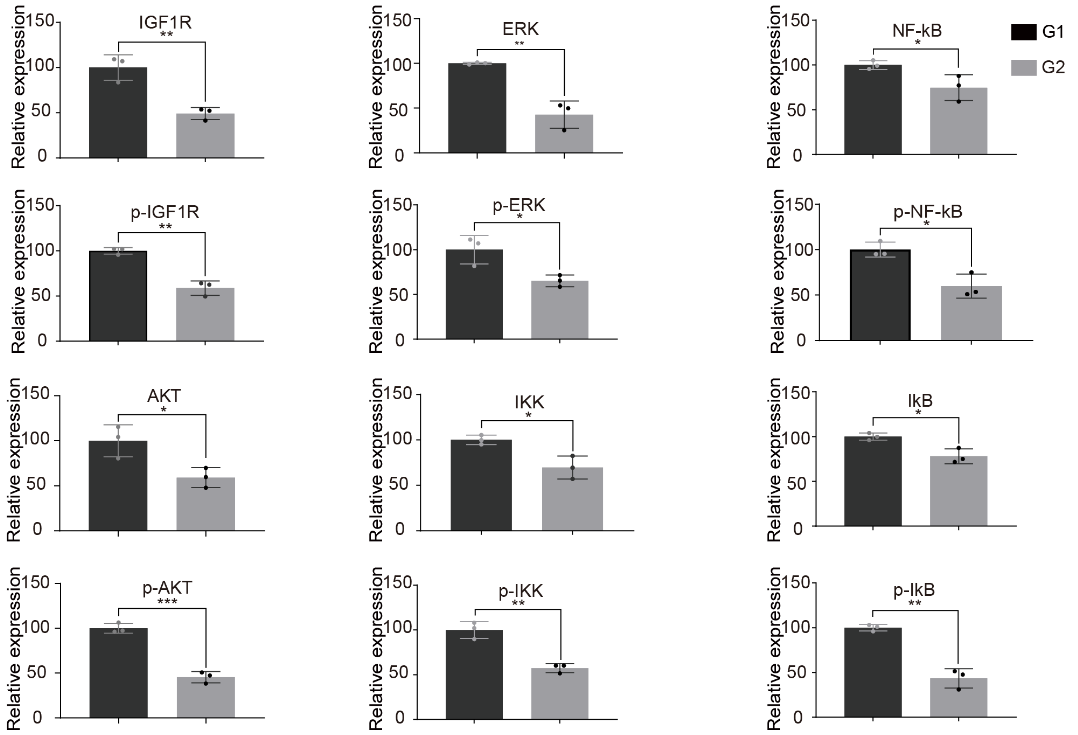

Fig. S61. Quantitative analysis of relative IGF1R, p-IGF1R, AKT, p-AKT, ERK, p-ERK, IKK, p-IKK, NF-κB, p-NF-κB, IκB and p-IκB protein expression from the western blotting images in Supplementary Fig. S60. G1: Control, G2: HA-BPY-GEF-NPs+Laser. Data are shown as mean ± SD (n = 3). *p<0.05, **p<0.01, and ***p<0.001.

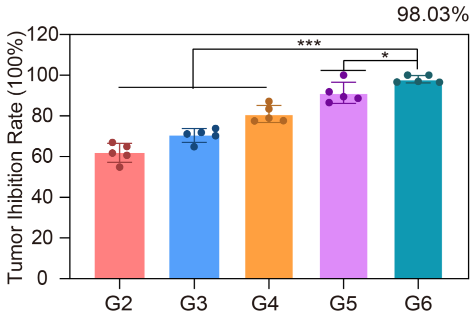

Fig. S62. Tumor growth inhibition of PC-9 tumor bearing tumor mice after different treatment groups. G2: free GEF, G3: HA-BPY-GEF-NPs, G4: HA-GEF-NPs, G5: HA-BPY-NPs+Laser, G6: HA-BPY-GEF-NPs+Laser. Data are shown as mean ± SD (n = 5). *p<0.05, **p<0.01, and ***p<0.001.

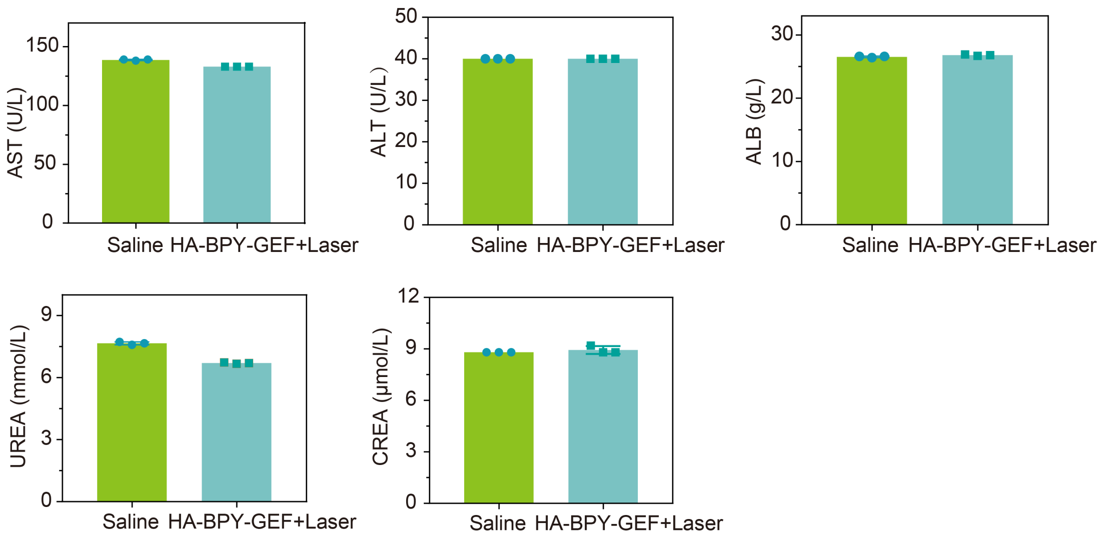

Fig. S63. Blood biochemistry analysis of PC-9 tumor bearing tumor mice after treatment with saline or HA-BPY-GEF-NPs+Laser (n = 3). (ALT: alanine aminotransferase, AST: aspartate aminotransferase, ALB: albumin, UREA: urea, CREA: creatinine)

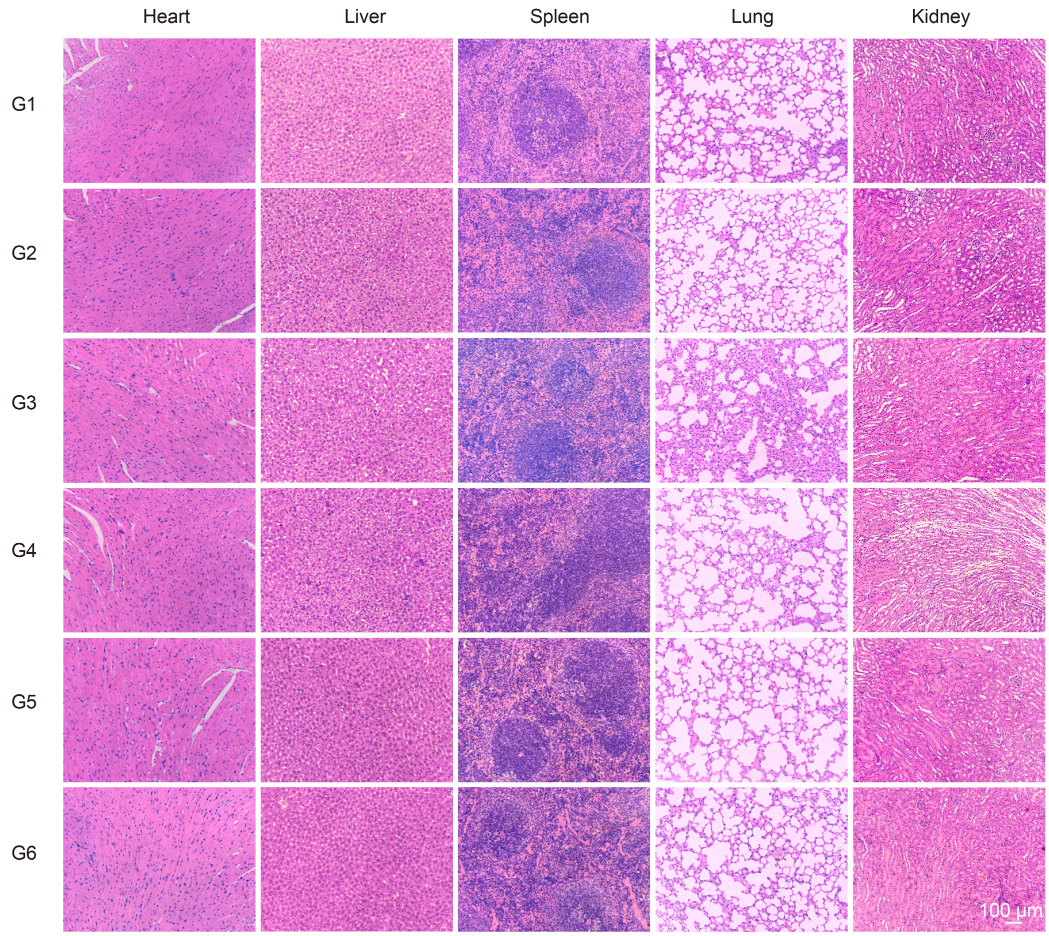

Fig. S64. Representative H&E staining slides of major organs harvested from PC-9 tumor bearing tumor mice after different treatments. No obvious histological damages were observed in each treatment group. G1: Saline, G2: free GEF, G3: HA-BPY-GEF-NPs, G4: HA-GEF-NPs, G5: HA-BPY-NPs+Laser, G6: HA-BPY-GEF-NPs+Laser. Scale bar: 100 μm.

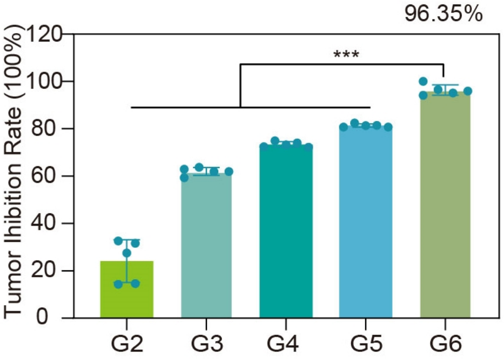

Fig. S65. Tumor growth inhibition rate of different treatment groups in PC9-GR tumor bearing tumor mice. G2: free GEF, G3: HA-BPY-GEF-NPs, G4: HA-GEF-NPs, G5: HA-BPY-NPs+Laser, G6: HA-BPY-GEF-NPs+Laser. Data are shown as mean ± SD (n = 5). *p<0.05, **p<0.01, and ***p<0.001.

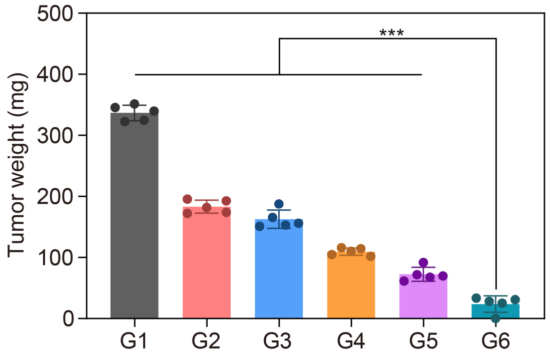

Fig. S66. Tumor tissue weights in different groups at the end of the experiment. G1: Saline, G2: free GEF, G3: HA-BPY-GEF-NPs, G4: HA-GEF-NPs, G5: HA-BPY-NPs+Laser, G6: HA-BPY-GEF-NPs+Laser. Data are shown as mean ± SD ($n = 5$). *p<0.05, **p<0.01, and ***p<0.001.

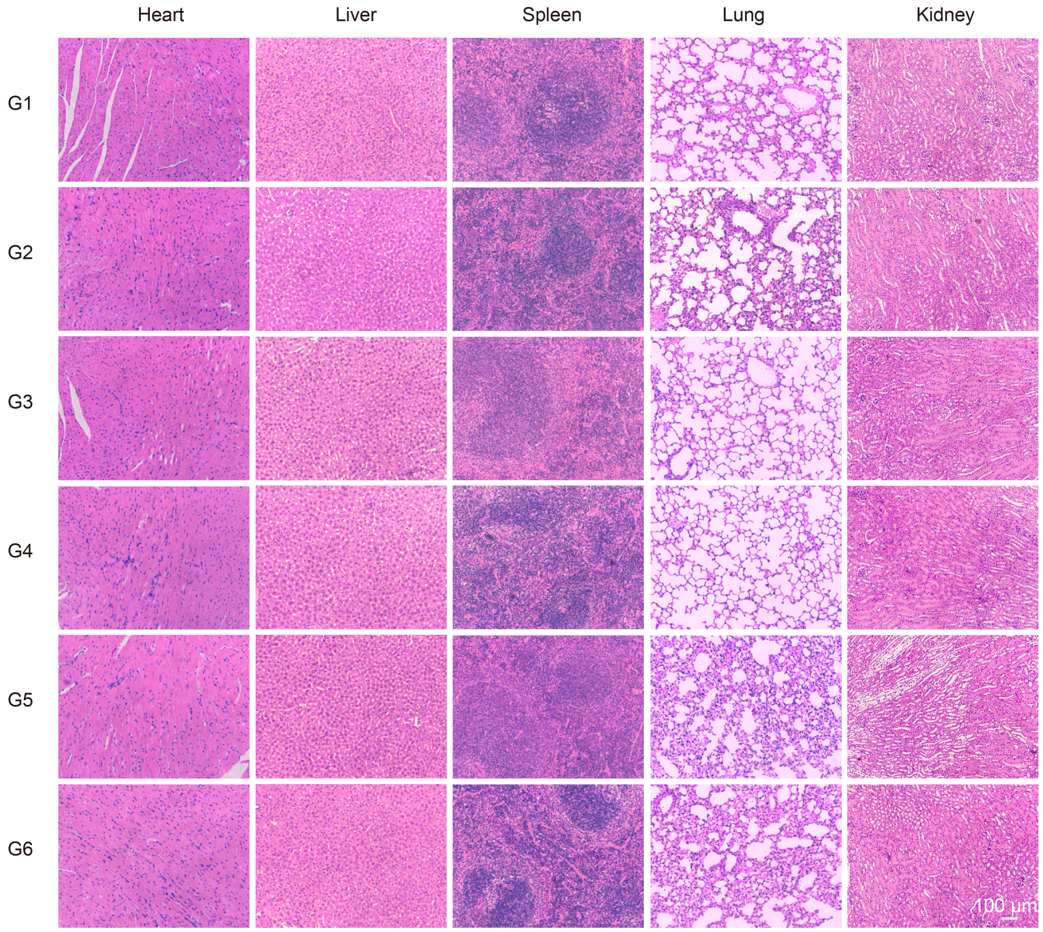

Fig. S67. Representative H&E staining slides of major organs harvested from PC9-GR tumor bearing tumor mice after different treatments. No obvious histological damages were observed in each treatment group. G1: Saline, G2: free GEF, G3: HA-BPY-GEF-NPs, G4: HA-GEF-NPs, G5: HA-BPY-NPs+Laser, G6: HA-BPY-GEF-NPs+Laser. Scale bar: 100 μm.

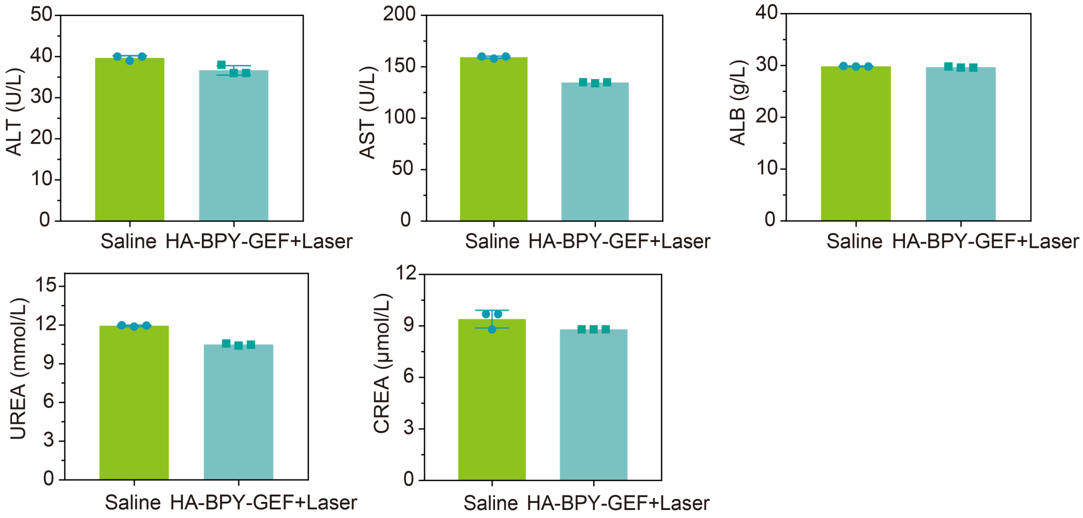

Fig. S68. Blood biochemistry analysis of PC9-GR tumor bearing tumor mice after treatment with saline or HA-BPY-GEF-NPs+Laser (n = 3). (ALT: alanine aminotransferase, AST: aspartate aminotransferase, ALB: albumin, UREA: urea, CREA: creatinine)

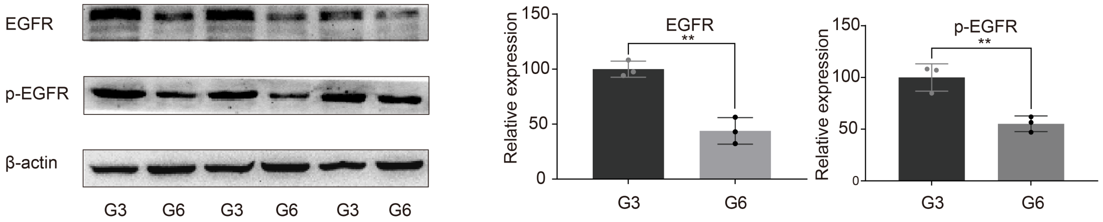

Fig. S69. Western blotting images of proteins expression in PC9-GR tumor tissues after different treatments. Quantification data. G3: HA-BPY-GEF-NPs, G6: HA-BPY-GEF-NPs+Laser. Data are shown as mean ± SD (n = 3). *p<0.05, **p<0.01, and ***p<0.001.

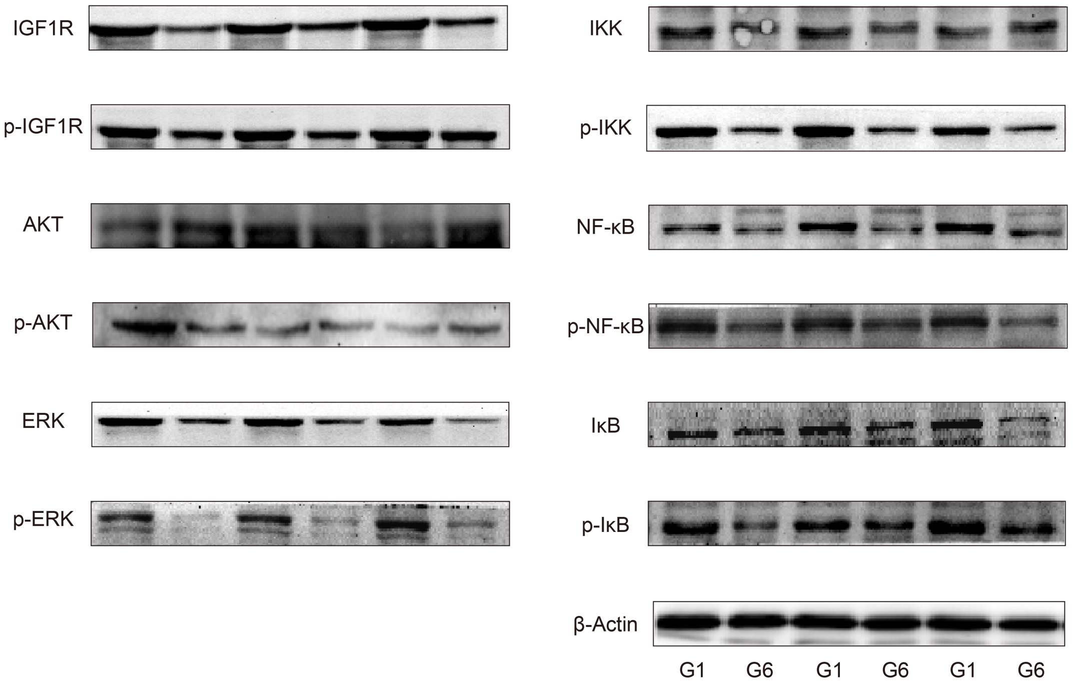

Fig. S70. Western blotting images of proteins expression in PC9-GR tumor tissues after different treatments. G1: Saline, G6: HA-BPY-GEF-NPs+Laser.

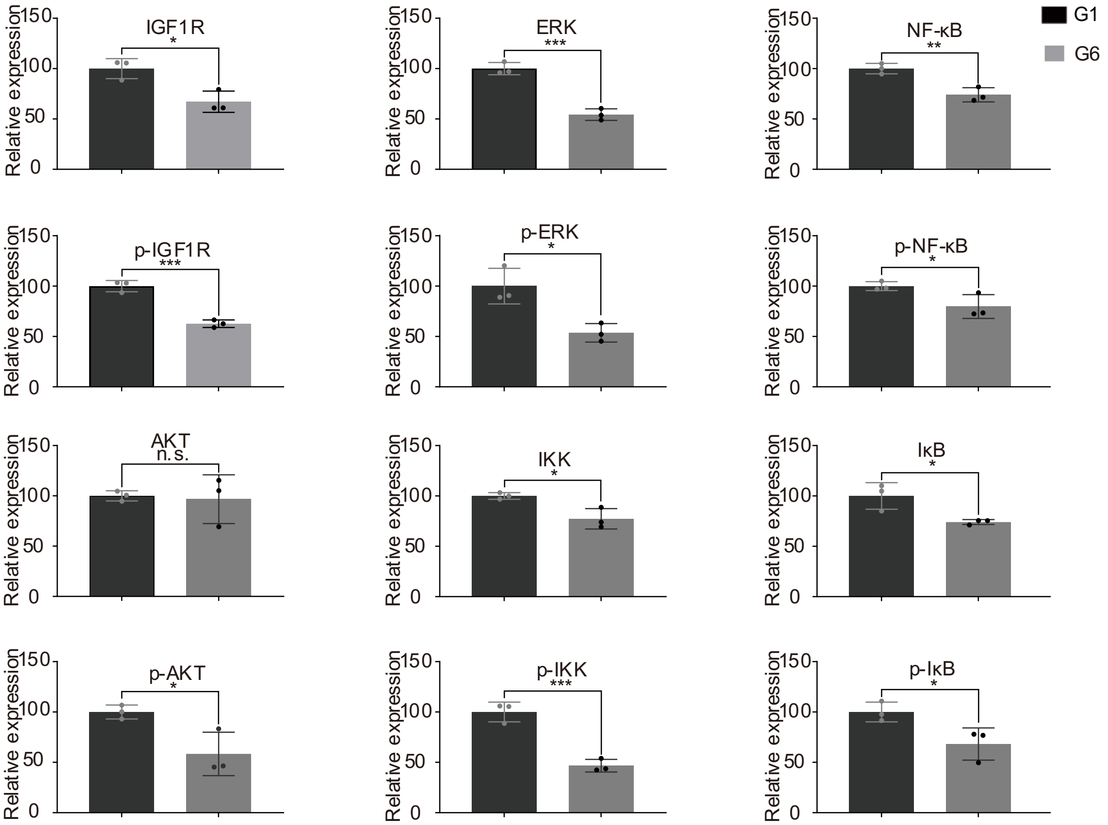

Fig. S71. Quantitative analysis of relative IGF1R, p-IGF1R, AKT, p-AKT, ERK, p-ERK, IKK, p-IKK, NF-κB, p-NF-κB, IκB and p-IκB protein expression from the western blotting images in Supplementary Fig. S70. G1: Saline, G6: HA-BPY-GEF-NPs+Laser. Data are shown as mean ± SD (*n* = 3). *p<0.05, **p<0.01, and ***p<0.001.

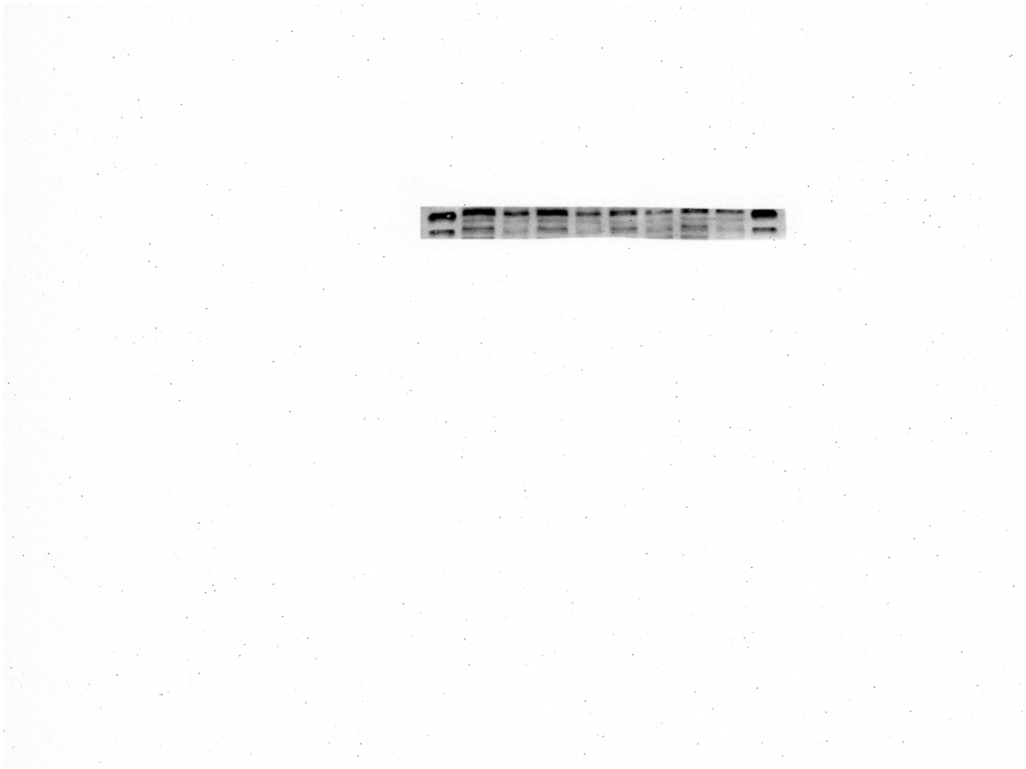
Original blot for Fig. 7J EGFR.

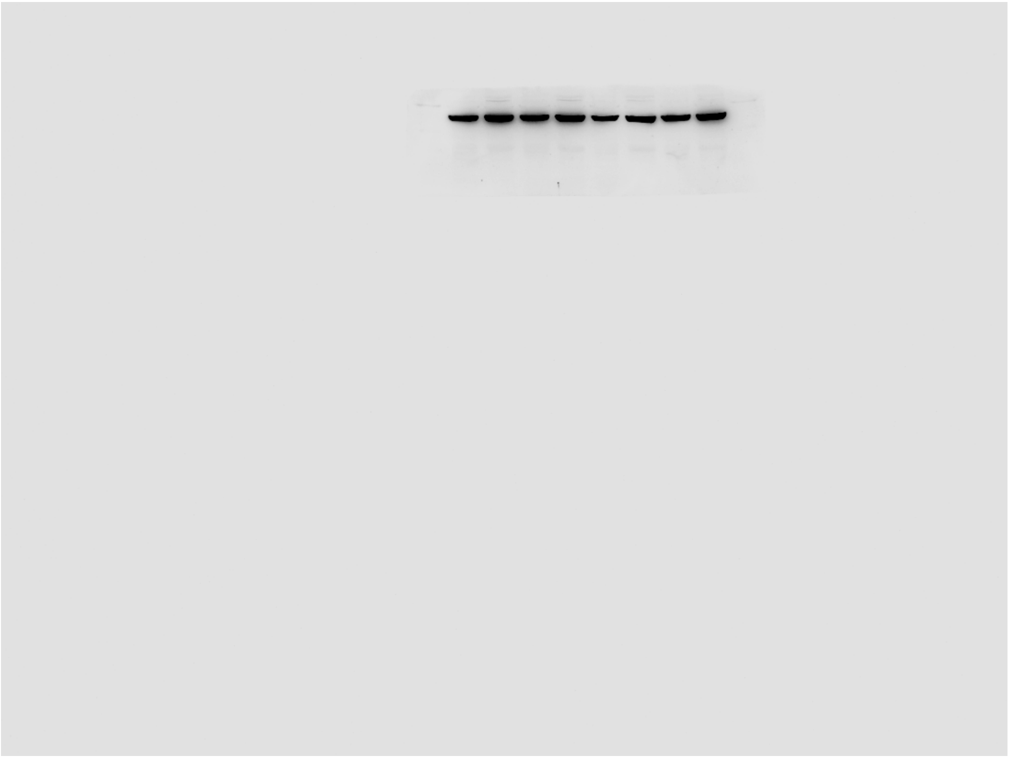
Original blot for Fig. S59 β-Actin.

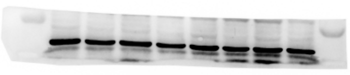

Original blot for Fig. S60 β-Actin.

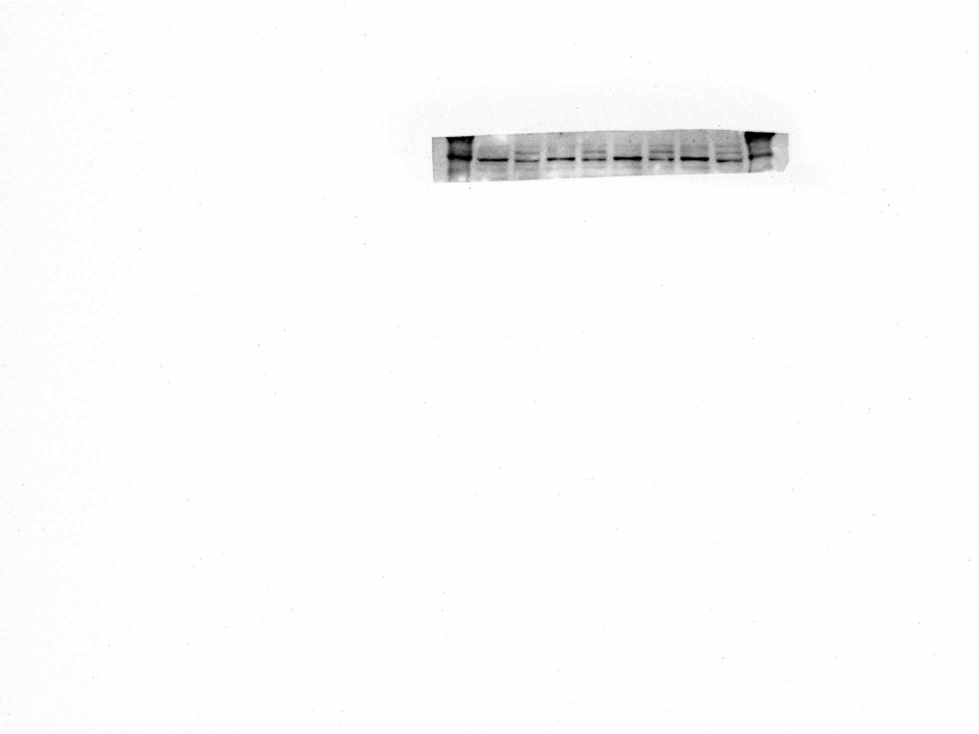

Original blot for Fig. S70 NF-KB.

REFERENCES AND NOTES

1. E. Sugiyama, Y. Togashi, Y. Takeuchi, S. Shinya, Y. Tada, K. Kataoka, K. Tane, E. Sato, G. Ishii, K. Goto, Y. Shintani, M. Okumura, M. Tsuboi, H. Nishikawa, Blockade of EGFR improves responsiveness to PD-1 blockade in EGFR-mutated non-small cell lung cancer. *Sci. Immunol.* **5**, eaav3937 (2020).

2. M. Wang, R. S. Herbst, C. Boshoff, Toward personalized treatment approaches for non-small-cell lung cancer. *Nat. Med.* **27**, 1345–1356 (2021).

3. P. Ö. Eser, R. M. Paranal, J. Son, E. Ivanova, Y. Kuang, H. M. Haikala, C. To, J. J. Okoro, K. H. Dholakia, J. Choi, Y. Eum, A. Ogino, P. Missios, D. Ercan, M. Xu, M. J. Poitras, S. Wang, K. Ngo, M. Dills, M. Yanagita, T. Lopez, M. Lin, J. Tsai, N. Floch, E. S. Chambers, J. Heng, R. Anjum, A. D. Santucci, K. Michael, A. G. Schuller, D. Cross, P. D. Smith, G. R. Oxnard, D. A. Barbie, L. M. Sholl, M. Bahcall, S. Palakurthi, P. C. Gokhale, C. P. Paweletz, G. Q. Daley, P. A. Jänne, Oncogenic switch and single-agent MET inhibitor sensitivity in a subset of EGFR-mutant lung cancer. *Sci. Transl. Med.* **13**, eabb3738 (2021).

4. J. J. Lin, S. V. Liu, C. E. McCoach, V. W. Zhu, A. C. Tan, S. Yoda, J. Peterson, A. Do, K. Prutisto-Chang, I. Dagogo-Jack, L. V. Sequist, L. J. Wirth, J. K. Lennerz, A. N. Hata, M. Mino-Kenudson, V. Nardi, S. H. I. Ou, D. S. W. Tan, J. F. Gainor, Mechanisms of resistance to selective RET tyrosine kinase inhibitors in RET fusion-positive non-small-cell lung cancer. *Ann. Oncol.* **31**, 1725–1733 (2020).

5. A. Passaro, P. A. Jänne, T. Mok, S. Peters, Overcoming therapy resistance in EGFR-mutant lung cancer. *Nat. Cancer* **2**, 377–391 (2021).

6. D. J. Konieczkowski, C. M. Johannessen, L. A. Garraway, A convergence-based framework for cancer drug resistance. *Cancer Cell* **33**, 801–815 (2018).

7. M. J. Niederst, J. A. Engelman, Bypass mechanisms of resistance to receptor tyrosine kinase inhibition in lung cancer. *Sci. Signal.* **6**, re6 (2013).

8. J. Rotow, T. G. Bivona, Understanding and targeting resistance mechanisms in NSCLC. *Nat. Rev. Cancer* **17**, 637–658 (2017).

9. S. Kummar, H. X. Chen, J. Wright, S. Holbeck, M. D. Millin, J. Tomaszewski, J. Zweibel, J. Collins, J. H. Doroshow, Utilizing targeted cancer therapeutic agents in combination: Novel approaches and urgent requirements. *Nat. Rev. Drug Discov.* **9**, 843–856 (2010).

10. X. Zhang, T. K. Maity, K. E. Ross, Y. Qi, C. M. Cultraro, M. Bahta, S. Pitts, M. Keswani, S. Gao, K. D. P. Nguyen, J. Cowart, F. Kirkali, C. Wu, U. Guha, Alterations in the global proteome and phosphoproteome in third generation EGFR TKI resistance reveal drug targets to circumvent resistance. *Cancer Res.* **81**, 3051–3066 (2021).

11. C. Gu, J. Ramos, U. Begley, P. C. Dedon, D. Fu, T. J. Begley, Phosphorylation of human TRM9L integrates multiple stress-signaling pathways for tumor growth suppression. *Sci. Adv.* **4**, eaas9184 (2018).

12. I. S. Harris, G. M. DeNicola, The complex interplay between antioxidants and ROS in cancer. *Trends Cell Biol.* **30**, 440–451 (2020).

13. X. Gu, Y. Qiu, M. Lin, K. Cui, G. Chen, Y. Chen, C. Fan, Y. Zhang, L. Xu, H. Chen, J.-B. Wan, W. Lu, Z. Xiao, CuS nanoparticles as a photodynamic nanoswitch for abrogating bypass signaling to overcome gefitinib resistance. *Nano Lett.* **19**, 3344–3352 (2019).

14. L. Zhao, J. Li, Y. Su, L. Yang, L. Chen, L. Qiang, Y. Wang, H. Xiang, H. P. Tham, J. Peng, Y. Zhao, MTH1 inhibitor amplifies the lethality of reactive oxygen species to tumor in photodynamic therapy. *Sci. Adv.* **6**, eaaz0575 (2020).

15. J. Wan, X. Zhang, D. Tang, T. Liu, H. Xiao, Biodegradable NIR-II pseudo conjugate polymeric nanoparticles amplify photodynamic immunotherapy via alleviation of tumor hypoxia and tumor-associated macrophage reprogramming. *Adv. Mater.* **35**, 2209799 (2023).

16. J. Xiong, J. C. H. Chu, W.-P. Fong, C. T. T. Wong, D. K. P. Ng, Specific activation of photosensitizer with extrinsic enzyme for precise photodynamic therapy. *J. Am. Chem. Soc.* **144**, 10647–10658 (2022).

17. P. Zhang, Y. Xiao, X. Sun, X. Lin, S. Koo, A. V. Yaremenko, D. Qin, N. Kong, O. C. Farokhzad, W. Tao, Cancer nanomedicine toward clinical translation: Obstacles, opportunities, and future prospects. *Med* **4**, 147–167 (2023).

18. J. Ouyang, A. Xie, J. Zhou, R. Liu, L. Wang, H. Liu, N. Kong, W. Tao, Minimally invasive nanomedicine: Nanotechnology in photo-/ultrasound-/radiation-/magnetism-mediated therapy and imaging. *Chem. Soc. Rev.* **51**, 4996–5041 (2022).

19. H. Chen, X. Zeng, H. P. Tham, S. Z. F. Phua, W. Cheng, W. Zeng, H. Shi, L. Mei, Y. Zhao, NIR-light-activated combination therapy with a precise ratio of photosensitizer and prodrug using a host-guest strategy. *Angew. Chem. Int. Ed. Engl.* **58**, 7641–7646 (2019).

20. S. Z. F. Phua, G. Yang, W. Q. Lim, A. Verma, H. Chen, T. Thanabalu, Y. Zhao, Catalase-integrated hyaluronic acid as nanocarriers for enhanced photodynamic therapy in solid tumor. *ACS Nano* **13**, 4742–4751 (2019).

21. Y. Feng, S. Qi, X. Yu, X. Zhang, H. Zhu, G. Yu, Supramolecular modulation of tumor microenvironment through pillar[5]arene-based host-guest recognition to synergize cancer immunotherapy. *J. Am. Chem. Soc.* **145**, 18789–18799 (2023).

22. T.-X. Zhang, Z.-Z. Zhang, Y.-X. Yue, X.-Y. Hu, F. Huang, L. Shi, Y. Liu, D.-S. Guo, A general hypoxia-responsive molecular container for tumor-targeted therapy. *Adv. Mater.* **32**, 1908435 (2020).

23. K. Yang, G. Yu, Z. Yang, L. Yue, X. Zhang, C. Sun, J. Wei, L. Rao, X. Chen, R. Wang, Supramolecular polymerization-induced nanoassemblies for self-augmented cascade chemotherapy and chemodynamic therapy of tumor. *Angew. Chem. Int. Ed. Engl.* **60**, 17570–17578 (2021).

24. Y. Wu, L. Sun, X. Chen, J. Liu, J. Ouyang, X. Zhang, Y. Guo, Y. Chen, W. Yuan, D. Wang, T. He, F. Zeng, H. Chen, S. Wu, Y. Zhao, Cucurbit[8]uril-based water-dispersible assemblies with enhanced optoacoustic performance for multispectral optoacoustic imaging. *Nat. Commun.* **14**, 3918 (2023).

25. H. Wang, S. Wang, H. Su, K.-J. Chen, A. L. Armijo, W.-Y. Lin, Y. Wang, J. Sun, K.-i. Kamei, J. Czernin, C. G. Radu, H.-R. Tseng, A supramolecular approach for preparation of size-controlled nanoparticles. *Angew. Chem. Int. Ed. Engl.*. **48**, 4344–4348 (2009).

26. R. Mejia-Ariza, L. Graña-Suárez, W. Verboom, J. Huskens, Cyclodextrin-based supramolecular nanoparticles for biomedical applications. *J. Mater. Chem. B* **5**, 36–52 (2017).

27. X. Tan, J. Huang, Y. Wang, S. He, L. Jia, Y. Zhu, K. Pu, Y. Zhang, X. Yang, Transformable nanosensitizer with tumor microenvironment-activated sonodynamic process and calcium release for enhanced cancer immunotherapy. *Angew. Chem. Int. Ed. Engl.* **60**, 14051–14059 (2021).

28. M. Tang, B. Chen, H. Xia, M. Pan, R. Zhao, J. Zhou, Q. Yin, F. Wan, Y. Yan, C. Fu, L. Zhong, Q. Zhang, Y. Wang, pH-gated nanoparticles selectively regulate lysosomal function of tumor-associated macrophages for cancer immunotherapy. *Nat. Commun.* **14**, 5888 (2023).

29. Y. Wang, Q. Tang, R. Wu, S. Sun, J. Zhang, J. Chen, M. Gong, C. Chen, X. Liang, Ultrasound-triggered piezocatalysis for selectively controlled NO gas and chemodrug release to enhance drug penetration in pancreatic cancer. *ACS Nano* **17**, 3557–3573 (2023).

30. L. Huang, J. Wan, H. Wu, X. Chen, Q. Bian, L. Shi, X. Jiang, A. Yuan, J. Gao, H. Wang, Quantitative self-assembly of photoactivatable small molecular prodrug cocktails for safe and potent cancer chemo-photodynamic therapy. *Nano Today* **36**, 101030 (2021).

31. Q. Luo, Z. Duan, X. Li, L. Gu, L. Ren, H. Zhu, X. Tian, R. Chen, H. Zhang, Q. Gong, Z. Gu, K. Luo, Branched polymer-based redox/enzyme-activatable photodynamic nanoagent to trigger STING-dependent immune responses for enhanced therapeutic effect. *Adv. Funct. Mater.* **32**, 2110408 (2022).

32. L. Gu, Z. Duan, X. Li, X. Li, Y. Li, X. Li, G. Xu, P. Gao, H. Zhang, Z. Gu, J. Chen, Q. Gong, K. Luo, Enzyme-triggered deep tumor penetration of a dual-drug nanomedicine enables an enhanced cancer combination therapy. *Bioact. Mater.* **26**, 102–115 (2023).

33. Z. Li, S. Li, Y. Guo, C. Yuan, X. Yan, K. S. Schanze, Metal-free nanoassemblies of water-soluble photosensitizer and adenosine triphosphate for efficient and precise photodynamic cancer therapy. *ACS Nano* **15**, 4979–4988 (2021).

34. L. Sun, F. Shen, L. Tian, H. Tao, Z. Xiong, J. Xu, Z. Liu, ATP-responsive smart hydrogel releasing immune adjuvant synchronized with repeated chemotherapy or radiotherapy to boost antitumor immunity. *Adv. Mater.* **33**, 2007910 (2021).

35. D. Hao, Q. Meng, B. Jiang, S. Lu, X. Xiang, Q. Pei, H. Yu, X. Jing, Z. Xie, Hypoxia-activated PEGylated paclitaxel prodrug nanoparticles for potentiated chemotherapy. *ACS Nano* **16**, 14693–14702 (2022).

36. L. Ge, C. Qiao, Y. Tang, X. Zhang, X. Jiang, Light-activated hypoxia-sensitive covalent organic framework for tandem-responsive drug delivery. *Nano Lett.* **21**, 3218–3224 (2021).

37. Y. Wu, X. Chang, G. Yang, L. Chen, Q. Wu, J. Gao, R. Tian, W. Mu, J. J. Gooding, X. Chen, S. Sun, A physiologically responsive nanocomposite hydrogel for treatment of head and neck squamous cell carcinoma via proteolysis-targeting chimeras enhanced immunotherapy. *Adv. Mater.* **35**, 2210787 (2023).

38. C. Ding, C. Chen, X. Zeng, H. Chen, Y. Zhao, Emerging strategies in stimuli-responsive prodrug nanosystems for cancer therapy. *ACS Nano* **16**, 13513–13553 (2022).

39. M. M. Zegota, M. A. Müller, B. Lantzberg, G. Kizilsavas, J. A. S. Coelho, P. Moscariello, M. Martínez-Negro, S. Morsbach, P. M. P. Gois, M. Wagner, D. Y. W. Ng, S. L. Kuan, T. Weil, Dual stimuli-responsive dynamic covalent peptide tags: Toward sequence-controlled release in tumor-like microenvironments. *J. Am. Chem. Soc.* **143**, 17047–17058 (2021).

40. L. K. B. Tam, J. C. H. Chu, L. He, C. Yang, K.-C. Han, P. C. K. Cheung, D. K. P. Ng, P.-C. Lo, Enzyme-responsive double-locked photodynamic molecular beacon for targeted photodynamic anticancer therapy. *J. Am. Chem. Soc.* **145**, 7361–7375 (2023).

41. X. An, A. Zhu, H. Luo, H. Ke, H. Chen, Y. Zhao, Rational design of multi-stimuli-responsive nanoparticles for precise cancer therapy. *ACS Nano* **10**, 5947–5958 (2016).

42. S. Dolui, B. Sahu, S. A. Mohammad, S. Banerjee, Multi-stimuli responsive sequence defined multi-arm star diblock copolymers for controlled drug release. *JACS Au* **3**, 2117–2122 (2023).

43. R. Yue, C. Zhang, L. Xu, Y. Wang, G. Guan, L. Lei, X. Zhang, G. Song, Dual key co-activated nanoplatform for switchable MRI monitoring accurate ferroptosis-based synergistic therapy. *Chem* **8**, 1956–1981 (2022).

44. T. Zhao, L. Chen, M. Liu, R. Lin, W. Cai, C.-T. Hung, S. Wang, L. Duan, F. Zhang, A. Elzatahry, X. Li, D. Zhao, Emulsion-oriented assembly for Janus double-spherical mesoporous nanoparticles as biological logic gates. *Nat. Chem.* **15**, 832–840 (2023).

45. Y. Shi, P. Wang, M. Li, T. Zhang, G. Han, C. Duan, A diode-like dye-Cu anisotropic junction in a coordination polymer as a logic state ratchet for intra-tumoral redox photomodulation. *Angew. Chem. Int. Ed. Engl.* **62**, e202219172 (2023).

46. B. Hou, L. Zhou, H. Wang, M. Saeed, D. Wang, Z. Xu, Y. Li, H. Yu, Engineering stimuli-activatable Boolean logic prodrug nanoparticles for combination cancer immunotherapy. *Adv. Mater.* **32**, 1907210 (2020).

47. C. Lu, Z. Li, N. Wu, D. Lu, X.-B. Zhang, G. Song, Tumor microenvironment-tailored nanoplatform for companion diagnostic applications of precise cancer therapy. *Chem* **9**, 3185–3211 (2023).

48. S. Erbas-Cakmak, S. Kolemen, A. C. Sedgwick, T. Gunnlaugsson, T. D. James, J. Yoon, E. U. Akkaya, Molecular logic gates: The past, present, and future. *Chem. Soc. Rev.* **47**, 2228–2248 (2018).

49. M. Sang, Y. Huang, L. Wang, L. Chen, Nawsherwan, G. Li, Y. Wang, X. Yu, C. Dai, J. Zheng, An "AND" molecular logic gate as a super-enhancers for de novo designing activatable probe and its application in atherosclerosis imaging. *Adv. Sci.* **10**, 2207066 (2023).

50. Y. Ma, J. Shang, L. Liu, M. Li, X. Xu, H. Cao, L. Xu, W. Sun, G. Song, X.-B. Zhang, Rational design of a double-locked photoacoustic probe for precise in vivo imaging of cathepsin B in atherosclerotic plaques. *J. Am. Chem. Soc.* **145**, 17881–17891 (2023).

51. Q. Fan, W. Xiong, H. Zhou, J. Yang, J. Feng, Z. Li, L. Wu, F. Hu, X. Duan, B. Li, J. Fan, Y. Xu, X. Chen, Z. Shen, An AND logic gate for magnetic-resonance-imaging-guided ferroptosis therapy of tumors. *Adv. Mater.* **35**, 2305932 (2023).

52. L. Jiang, X. Huang, D. Chen, H. Yan, X. Li, X. Du, Supramolecular vesicles coassembled from disulfide-linked benzimidazolium amphiphiles and carboxylate-substituted pillar[6]arenes that are responsive to five stimuli. *Angew. Chem. Int. Ed. Engl.* **56**, 2655–2659 (2017).

53. X. Fu, L. Hosta-Rigau, R. Chandrawati, J. Cui, Multi-stimuli-responsive polymer particles, films, and hydrogels for drug delivery. *Chem* **4**, 2084–2107 (2018).

54. L. Wu, J. Huang, K. Pu, T. D. James, Dual-locked spectroscopic probes for sensing and therapy. *Nat. Rev. Chem.* **5**, 406–421 (2021).

55. W.-C. Geng, J. L. Sessler, D.-S. Guo, Supramolecular prodrugs based on host-guest interactions. *Chem. Soc. Rev.* **49**, 2303–2315 (2020).

56. H. Du, S. Zhao, Y. Wang, Z. Wang, B. Chen, Y. Yan, Q. Yin, D. Liu, F. Wan, Q. Zhang, Y. Wang, pH/cathepsin B hierarchical-responsive nanoconjugates for enhanced tumor penetration and chemo-immunotherapy. *Adv. Funct. Mater.* **30**, 2003757 (2020).

57. X. Song, H. Cai, Z. Shi, Z. Li, X. Zheng, K. Yang, Q. Gong, Z. Gu, J. Hu, K. Luo, Enzyme-responsive branched glycopolymer-based nanoassembly for co-delivery of paclitaxel and Akt inhibitor toward synergistic therapy of gastric cancer. *Adv. Sci.* **11**, 2306230 (2024).

58. H. Cai, P. Tan, X. Chen, M. Kopytynski, D. Pan, X. Zheng, L. Gu, Q. Gong, X. Tian, Z. Gu, H. Zhang, R. Chen, K. Luo, Stimuli-sensitive linear-dendritic block copolymer-drug prodrug as a nanoplatform for tumor combination therapy. *Adv. Mater.* **34**, 2108049 (2022).

59. H. Xia, M. Qin, Z. Wang, Y. Wang, B. Chen, F. Wan, M. Tang, X. Pan, Y. Yang, J. Liu, R. Zhao, Q. Zhang, Y. Wang, A pH-/enzyme-responsive nanoparticle selectively targets endosomal Toll-like receptors to potentiate robust cancer vaccination. *Nano Lett.* **22**, 2978–2987 (2022).

60. I. P. Claver, H. Zhou, Enzymatic hydrolysis of defatted wheat germ by proteases and the effect on the functional properties of resulting protein hydrolysates. *J. Food Biochem.* **29**, 13–26 (2005).

61. M. Chen, C. Wang, X. Wang, Z. Tu, Z. Ding, Z. Liu, An "AND" logic-gated prodrug micelle locally stimulates antitumor immunity. *Adv. Mater.* **36**, 2307818 (2024).

62. X. Wang, S. He, P. Cheng, K. Pu, A dual-locked tandem fluorescent probe for imaging of pyroptosis in cancer chemo-immunotherapy. *Adv. Mater.* **35**, 2206510 (2023).

63. F. Sun, Q. Zhu, T. Li, M. Saeed, Z. Xu, F. Zhong, R. Song, M. Huai, M. Zheng, C. Xie, L. Xu, H. Yu, Regulating glucose metabolism with prodrug nanoparticles for promoting photoimmunotherapy of pancreatic cancer. *Adv. Sci.* **8**, 2002746 (2021).

64. M. Yu, X. Xu, Y. Cai, L. Zou, X. Shuai, Perfluorohexane-cored nanodroplets for stimulations-responsive ultrasonography and O_2-potentiated photodynamic therapy. *Biomaterials* **175**, 61–71 (2018).

65. S. Banerjee, E. B. Veale, C. M. Phelan, S. A. Murphy, G. M. Tocci, L. J. Gillespie, D. O. Frimannsson, J. M. Kelly, T. Gunnlaugsson, Recent advances in the development of 1,8-naphthalimide based DNA targeting binders, anticancer and fluorescent cellular imaging agents. *Chem. Soc. Rev.* **42**, 1601–1618 (2013).

66. M. Guix, A. C. Faber, S. E. Wang, M. G. Olivares, Y. Song, S. Qu, C. Rinehart, B. Seidel, D. Yee, C. L. Arteaga, J. A. Engelman, Acquired resistance to EGFR tyrosine kinase inhibitors in cancer cells is mediated by loss of IGF-binding proteins. *J. Clin. Invest.* **118**, 2609–2619 (2008).

67. J. Li, Y. Anraku, K. Kataoka, Self-boosting catalytic nanoreactors integrated with triggerable crosslinking membrane networks for initiation of immunogenic cell death by pyroptosis. *Angew. Chem. Int. Ed. Engl.* **59**, 13526–13530 (2020).

68. M. Yuan, X. Yin, H. Zheng, C. Ouyang, Z. Zuo, H. Liu, Y. Li, Light harvesting and efficient energy transfer in dendritic systems: New strategy for functionalized near-infrared BF2-azadipyrromethenes. *Chem. Asian J.* **4**, 707–713 (2009).

69. T. Gollnest, T. D. de Oliveira, D. Schols, J. Balzarini, C. Meier, Lipophilic prodrugs of nucleoside triphosphates as biochemical probes and potential antivirals. *Nat. Commun.* **6**, 8716 (2015).

70. P. López Rivas, I. Ranđelović, A. R. M. Dias, A. Pina, D. Arosio, J. Tóvári, G. Mező, A. Dal Corso, L. Pignataro, C. Gennari, Synthesis and biological evaluation of paclitaxel conjugates involving linkers cleavable by lysosomal enzymes and αVβ3-integrin ligands for tumor targeting. *Europea. J. Org. Chem.* **2018**, 2902–2909 (2018).

71. L. Zhang, D. Duan, Y. Liu, C. Ge, X. Cui, J. Sun, J. Fang, Highly selective off–on fluorescent probe for imaging thioredoxin reductase in living cells. *J. Am. Chem. Soc.* **136**, 226–233 (2014).
